# Agent-based modelling of the early stages of actin polymerisation required to drive endocytosis in *Saccharomyces cerevisiae*

**DOI:** 10.1038/s41598-025-14248-w

**Published:** 2025-08-07

**Authors:** Lewis P. Hancock, Ellen G. Allwood, John S. Palmer, Kathryn R. Ayscough, Mike P. Williamson

**Affiliations:** School of Biosciences, University of Sheffield, Sheffield, S10 2TN UK

**Keywords:** Endocytosis, Actin nucleation, Agent-based model, Yeast, SH3 domain, Endocytosis, Computational models

## Abstract

Endocytosis is critical. Its complexity means that many aspects remain poorly understood. We have developed an agent-based model covering key components of actin filament generation in endocytosis in *Saccharomyces cerevisiae.* The model incorporates realistic values for rates, affinities, concentrations, and mobilities, and reproduces essential features of endocytosis, from the arrival of WASp/Las17 and its inhibitor Sla1 at the membrane up to the burst of actin polymerisation. The model yields relative rates and affinities for interactions that cannot be measured experimentally, and places limitations on plausible scenarios. Specifically, it reveals three novel findings. First, Las17 must form multimeric complexes. Second, *de novo* F-actin nucleation occurs in two stages, involving the slow formation of linear trimers, followed by rapid polymerisation once an additional actin monomer is positioned at the side of the aligned monomers. Third, competition between SH3 domains and other factors, including actin, is critical to ensure on/off switching. This requires: (1) tandem domains binding to adjacent polyproline sites outcompeting single domains; (2) these tandem domains being weakened in overall affinity through a reduction in avidity by competition with single SH3 domains. We conclude with a pathway that proposes how controlled actin polymerisation occurs, and raises implications for further testing.

**Supplementary Information:**

The online version contains supplementary material available at 10.1038/s41598-025-14248-w.

## Introduction

Endocytosis is the process by which the cell membrane invaginates and forms an endocytic vesicle, facilitating internalisation of material at the cell surface. In *Saccharomyces cerevisiae*, it follows a highly reproducible pathway^1,2^. Early coat components localise to the membrane for between 40 s and several minutes^3^ followed by cargo binding and recruitment of late coat proteins, including Las17 and its inhibitor Sla1 in the SLAC complex^4^. Subsequent timings are highly predictable. Proteins arrive at the endocytic patch in a defined order: Sla1 binds to membrane, clathrin and cargo, while Las17 remains in the plane of the membrane. Separation of Las17 from Sla1 is proposed to expose actin binding sites^5^. In mammalian cells there is a pre-existing cortical F-actin cytoskeleton, but yeast does not have this network^6^ so G-actin monomers must be recruited for *de novo* nucleation and filament polymerisation. Las17 nucleates short linear F-actin filaments^7^ which recruit and activate Arp2/3 to nucleate further polymerisation of filaments from the side of the mother filaments, leading to formation of a branched network, which supports membrane invagination^8^. Other proteins are then recruited. Our focus here is on the early stages of actin nucleation and polymerisation, particularly its regulation.

Las17 is the yeast homolog of mammalian WASp^9^ and contains an N-terminal WH1 domain that binds membranes^7^, eight pentaproline sequences, and a C-terminal VCA region, which binds monomeric actin and Arp2/3. Much of Las17 is natively unstructured. Las17 is both an activator of the Arp2/3 complex, facilitating formation of a branched network, and can polymerise actin in the absence of Arp2/3^10^. Formation of these initial linear filaments is highly regulated. The most N-terminal polyproline sequences in Las17 (residues 300–422) bind to SH3 domains, and we have recently shown that these sites also bind three G-actin monomers^5^. We recently showed^5^ that a key event in actin nucleation is that Sla1 is displaced from its complex with Las17, exposing the N-terminal polyproline sequences in Las17 and allowing them to bind to actin, causing polymerisation.

The events described here are based on many observations, but do not *explain* how these proteins organise themselves to produce such a well-orchestrated sequence. Key components involved early in the process are actin, Las17 and Sla1. Many other proteins come and go at the endocytic patch in tightly regulated sequence; 11 of these contain SH3 domains, constituting about half of all yeast SH3s^11–13^. Rather than consider these individually, we modelled a generalised ”cloud” of SH3 domains.

We aimed to construct a realistic simple model of early events in endocytosis, up to the arrival of Arp2/3. Even if deficient in some respects, such a model has important benefits. If it reproduces actin polymerisation with realistic rates, concentrations, and outcomes, then we can have some confidence that it contains the essential elements. A model can be easily modified, allowing us rapidly to model the values of unknown parameters, and investigate the relative importance of different features or components. Importantly, a model allows us to test different hypotheses and scenarios, and thus to manipulate the system in ways that are not possible experimentally. This ability to test hypotheses has allowed us to construct a self-organising mechanism that fits known experimental details, reveals unexpected emergent phenomena^14^ and predicts many testable outcomes. We note that although it is parametrised to model in vitro events, it can be extended to model the in vivo situation.

## An agent-based model of the initiation of endocytosis

Models based on ordinary differential equations (ODEs) are time-efficient but require well-defined kinetic parameters, which means that they are not suitable for this application, because many parameters are not known. Molecular dynamics (MD) models are time-intensive, and modelling a 20-second timecourse by MD is not feasible. We therefore used agent-based modelling (ABM), specifically Flame GPU 1^15^. In ABM, all the components of the system (here, protein domains or polyproline sequences) are autonomous agents. In each step they follow rules: they can diffuse, rotate, bind, or unbind. In the simulations described here, we typically used a timestep of 1 µs, with thousands of protein molecules. This meant that statistically meaningful calculations could be obtained from a single run, and that a simulated period of one second could be calculated within approximately 6 h computational time on a home-built workstation. Our code is available on Github, and is described in Supplementary Material. Related models have been used previously, mainly Cytosim^16,17^ which combines a Langevin dynamics calculation to calculate the mechanics of a filamentous network, with a stochastic engine to simulate binding, unbinding and diffusion, and has been applied to a range of cytoskeletal modelling problems, including to the analysis of actin polymerisation^18,19^. A related stochastic growth model has also been described^20,21^. Compared to these models, our model focuses more on protein interactions, and less on geometry.

**Fig. 1:**
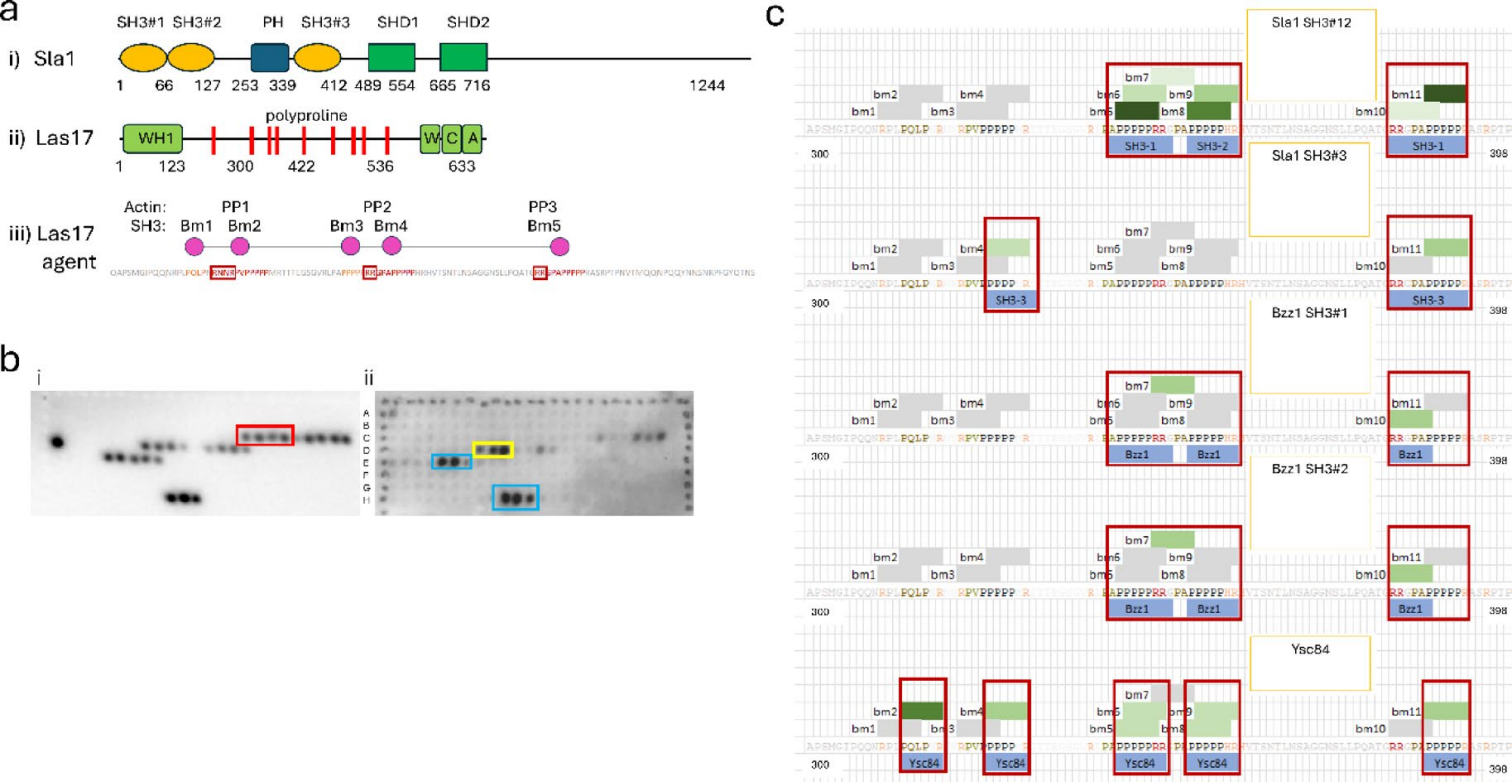
Agents and their binding sites. (**a**) Structures of agents. (i) Sla1. (ii) Las17. In the C-terminal VCA region, W is a WH2 domain, which binds monomeric actin, and C is a central Arp2/3 binding region. (iii) A Las17 agent. PP1-3 denote the three actin-binding sites, and Bm1-5 the five PxxP SH3-binding motifs. The 300–422 peptide sequence which this represents is also shown, with red denoting the actin-binding sites, orange representing non-actin binding sequences that contain a PxxP motif, and red boxes framing the arginine pairs that increase actin interactions. (**b**) Spot arrays. Each spot is a different synthetic peptide of 12 residues long (except for the group of three intense spots on row H, which are longer). More intense spots indicate tighter binding. Probes are (i) the SH3 domain from Ysc84 and (ii) the SH3 domain from Rvs167, representing a typical cloud SH3. Red box in A contains the sequence PQLP in Bm1 (Fig. 1aiii), showing that Ysc84 binds to Bm1; yellow box in B contains the PPPPP sequence from Bm3 (PP2a); cyan box in B contains the PPPPP sequence from Bm5 (PP3). (**c**) Predicted SH3 binding locations for computational modelling. The 300–422 residue sequence of Las17 was annotated with black font representing polyproline tracts, gold representing core PxxP motifs that are located outside of the tracts, salmon for positive residues within three residues of a proline and red for the two pairs of arginines known to interact with actin. Each PxxP site was then annotated as bm*x* to establish an SH3 binding site. These sites were extended to an arginine if they fit either the class I (+ xxPxxP) or class II (PxxPx+) definition^22^. Ultimately, 11 SH3 binding motifs were identified (bm1-bm11). Each SH3 domain was mapped to the binding motifs indicated from spot array data (Fig. 1b). This is shown under the appropriate sequence and surrounded with a red box. All motifs not expected to bind the SH3 domain (either due to being located outside of the red boxes, or being of the wrong classification) are shown in grey. Binding motifs likely to bind the indicated SH3 domains are then coloured green with progressively darker shades indicating a higher score. See Methods for more detail.

The key component that must be recruited to the plasma membrane is Las17. In vitro experiments using truncated versions indicated that the most important region for actin nucleation is the N-terminal polyproline repeats (Fig. 1a)^7,10^. We carried out spot-array experiments using overlapping peptides (Fig. 1b), which showed that polyproline sequences 1, 2 and 3 display the tightest binding to SH3 domains. Pairs of arginine residues separated by three residues from the pentaproline sequences strengthen the SH3 binding affinity^12^. There are two such pairs, at residues 349–350 and 382–383, the first of which is between the closely spaced polyprolines 2a and 2b, and the second is adjacent to 3 (Fig. 1a, red boxes). There are two other arginines in an RXXR motif upstream of polyproline 1, which is also important^5^. Therefore, our model has three polyproline binding sites on Las17, with spacings corresponding to polyprolines (PP) 1, 2b and 3. We recently showed^5^ that these three sites also bind to actin, and that binding of actin and SH3s is competitive. A detailed analysis of these data allowed us to identify the most likely binding interactions (Fig. 1c), described in detail in Supplementary Material.

The agents used in the model were Las17, Sla1, actin, Ysc84, Bzz1, and the SH3 cloud (Lsb3, Myo3, Myo5, Bbc1, Rvs167, Abp1). Sla1 and Bzz1 are interesting SH3 proteins because they contain multiple SH3 domains (three in Sla1 and two in Bzz1), while Bzz1 and Ysc84 were selected because they arrive at the endocytic patch after Las17 but before Arp2/3^12,23^.

### Parametrisation of the model

Monomeric G-actin is typically studied in G-buffer, to prevent polymerisation. Some affinities were therefore measured in this buffer. However, protein affinities in G-buffer are markedly stronger than those measured in more physiological solutions like PBS. The modelling aimed to understand endocytosis in vivo, and so the PBS values were used (or estimated from G-buffer values). Thus, we model behaviour in vitro in PBS, which we then extend to in vivo. The parameters used in the model are listed in Table 1. Most of these values were reported in^5^ using either microscale thermophoresis (MST) or biolayer interferometry (BLI). When parameters were measured using different techniques or different protein preparations, the values usually agreed within a factor of 2, except that affinities measured in G-buffer were stronger by a factor of roughly 10. Our aim in this parameter set is to have correct relative rates and affinities: the absolute values will depend on solution conditions. In the model, protein concentrations are determined by the number of molecules. We used 1000 molecules of Las17, 1-3000 Sla1 and 16,667 G-actin, within a box of size 1767 × 1767 × 1767 nm^3^ corresponding to concentrations of 0.3 µM Las17, 0.3–0.9 µM Sla1 and 5 µM G-actin, which match estimates of concentrations at the membrane surface^13^ and our experimental conditions^5,7^. Further details are in Supplementary Material.

**Table 1:** Model parameters.

Peptide	1a affinity	1b affinity	2a affinity	2b affinity	3 affinity
Sla1 SH3#1	-	-	7.5 ^a^	-	7.5 ^a^
Sla1 SH3#2	-	-	-	20 ^b^	-
Sla1 SH3#3	-	30 ^b^	-	-	30 ^b^
Cloud SH3	-	-	1	1	-
Ysc84 SH3	2.2 ^a^	4.4 ^a^	4.4 ^a^	4.4 ^a^	4.4 ^a^
Bzz1 SH3#1				7.5 ^c^	
Bzz1 SH3#2					7.5 ^c^
Actin	-	11.5 ^d^	-	3.5 ^d^	6.5 ^d^
Default values^e^					
SH3 domain base rates			Actin base rates		
*k*_on_ (µM^−1^ s^−1^)	*k*_off_ (s^−1^)	*K*_d_ (µM)	*k*_on_ (µM^−1^ s^−1^)	*k*_off_ (s^−1^)	*K*_d_ (µM)
0.98 × 10^9^	63.7	0.065	7 × 10^6^	0.17	0.024
Other parameter values					
Binding radius	Barbed end *k*_on_	Barbed end *k*_off_	Pointed end *k*_on_	Pointed end *k*_off_	
7.346 nm^f^	11.6 M^−1^ s^−1^	1.4 s^−1^	1.3 M^−1^ s^−1^	0.8 s^−1^	
Las17 dimerisation affinity when cooperatively binding actin	Side actin *k*_on_	Side actin* k*_off_	Ysc83-YAB affinity for actin		
0.15 µM^g^	2.18 M^−1^ s^−1^	1.3 × 10^3^ s^−1^	0.15 µM		

Affinities are in µM. Where the table lists no value, it was modelled as having no interaction. ^a^Obtained using BLI^5^. ^b^Calculated from the model. ^c^Uses the Sla1-SH3#1 affinity obtained using BLI. ^d^Measured in G-buffer using MST and adjusted. ^e^For each interaction the affinity (*K*_d_) is determined by the ratio between *k*_on_ and *k*_off_. If the affinity is stronger than that obtained from default values, *k*_on_ is increased and *k*_off_ is decreased by the same ratio to give the correct affinity. ^f^This value is the maximum permitted separation for binding to be allowed and is given by $\:r=\sqrt[3]{\frac{3{k}_{on}\varDelta\:t}{4\pi\:{10}^{3}{N}_{a}}}\:$ where the on-rate *k*_on_ is the diffusion-controlled on-rate, assumed to be 10^8^ M^−1^ s^−1^^24^, *Δt* is the timestep (1 µs), and *N*_*a*_ is Avogadro’s number^25^. Each individual interaction was then assigned a binding probability *P*, determined by the on-rate defined for that interaction and listed in Table 1, given by *P* = 3 *k*_on_*Δt*/4000π*N*_*a*_*r*^3^, and an unbinding probability given by the timestep multiplied by *k*_off_. ^g^Taken from^26^.

### Two possible mechanisms of actin nucleation

**Fig. 2:**
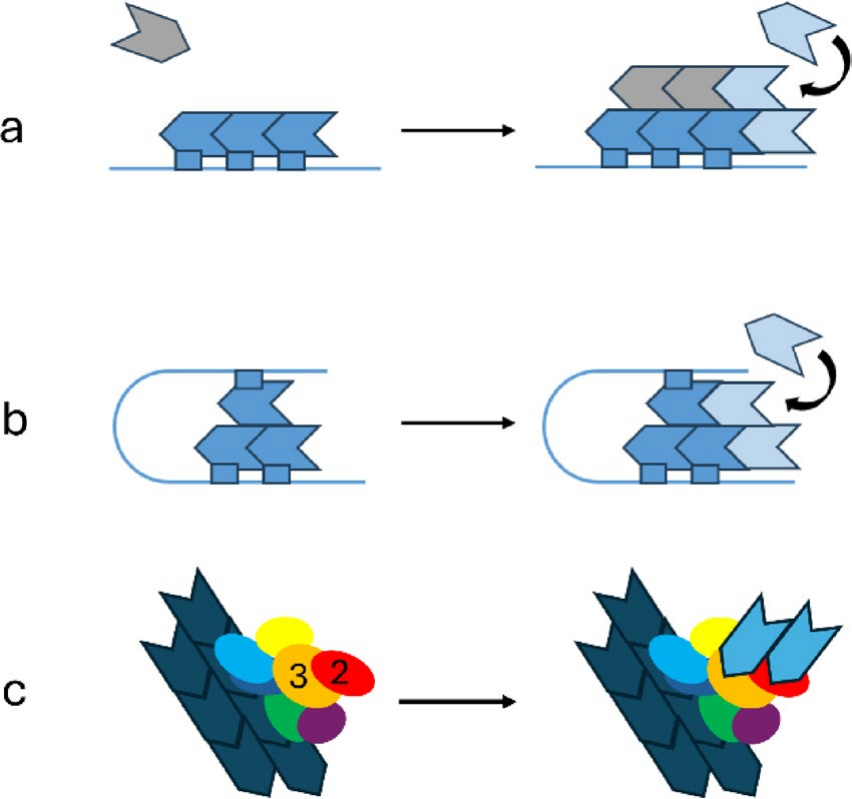
Linear and cross-filament nucleators. Complexes on the left represent early nucleation whilst complexes on the right show the subsequent seed elongation. Actin monomers are shown as arrow heads with the indent representing the barbed end (hydrophobic cleft). Actin monomers bound to each nucleator are shown in dark blue while actin subunits that are recruited post seed formation are shown in light blue. (**a**) Linear tandem nucleators require additional monomers (in grey) to bind laterally prior to completion of the seed, to form the start of the second F-actin filament: an inherently slow process because the off-rate for the first grey domain is fast compared to the rate of filament elongation. (**b**) Cross-filament nucleators hold the monomers for initiation of both filaments in place, shown here as an intramolecular arrangement. (**c**) Arp2/3 essentially acts as a cross-filament nucleator, with Arp2 (red) and Arp3 (orange) binding the pointed ends of a pair of new filaments. Based on^31^.

Las17 both nucleates actin and catalyses polymerisation. Two mechanisms of *de novo* nucleation have been suggested for Las17 (Fig. 2). First: it can nucleate actin by linear tandem nucleation, in which it binds three G-actin monomers in a row (Fig. 2a). Similar nucleation mechanisms have been described for Spire and JMY, which both possess multiple WH2 domains that have been shown to nucleate actin^27–30^. Support for this mechanism comes from examination of the number of amino acids separating the proline-rich regions (Supplementary Table S1), which suggests that linear nucleators have a spacing of about 30 residues, matching the spacing found in Las17.

An alternative Arp2/3-independent nucleation mechanism has also been described, namely cross-filament nucleation. Linear nucleation only forms one of the two F-actin strands; consequently, formation of F-actin from this nucleus is slow, because initiation of the second strand requires addition of G-actin at the side of the linear nucleus, and the off-rate for this monomer is very fast, implying a very low population of competent nuclei. The alternative, cross-filament, mechanism also requires three monomers, with the first two forming a linear tandem, but now the third is added laterally, forming the start of the second strand (Fig. 2b)^32^. Here we refer to such an entity as a seed. F-actin nucleation from the cross-filament seed is considerably faster than from the linear trimer (the nucleus)^33^. It is proposed that Cobl can nucleate F-actin cross filament, because its third WH2 domain has a long linker connecting it to the first two (Supplementary Table S1)^28,30^. Arp2/3 (Fig. 2c) also effectively forms a cross-filament seed, leading to the rapid formation of F-actin branches^31^and formins are also thought to form cross-filament seeds^34^. In support of this binary classification, in vitro assays using the tandem nucleator Spire require high concentrations of Spire (0.5-1 µM) to match the rates produced using 20 nM Arp2/3^27^. The activity of Cobl is roughly comparable to Arp2/3, with a maximum nucleation rate being reached at ~ 40 nM^28^.

It is not clear whether the actin polymerisation required for endocytosis is initiated by linear tandem nucleation or by cross-filament nucleation. In Sect. “Actin nucleation”, we use the model to investigate these two possibilities.

## Results from the model

### Determination of unknown affinities

Some affinities (labelled b in Table 1) could not be measured experimentally. In these cases, values were estimated using modelling. An example is the affinity of Las17 for the second Sla1 SH3 domain. The first and second domains (SH3#1 and SH3#2) are adjacent in the sequence. We were able to express and purify SH3#1, which is correctly folded in solution (Supplementary Figure S1), and measure binding affinities to Las17 using BLI^5^. However, SH3#2 could not be obtained alone and appears to be unfolded in the absence of SH3#1. The affinity of Las17(300–422) for SH3#1 was measured as 7.5 µM, and for the SH3#1-2 pair as 87 nM^5^. Because of the proximity of the two domains (and the short distance between Las17 polyproline sequences) one would expect a weak binding to SH3#2 alone, producing the (relatively small) 100-fold increase in affinity measured. Comparison of the model with data simulated using an ordinary differential equation gave an affinity of 20 µM (Supplementary Figure S2). Several other affinities were estimated using the model; others were modelled as being equal (for example, the affinities of Sla SH3#1 for sites PP2a and PP3), while some domains are assumed not to bind (Table 1).

The model was tested extensively, to check its reproducibility and sensitivity to variation in parameter values. Results of these tests are described in Supplementary Figs S3-4.

### Actin nucleation

As discussed in the section "Two possible mechanisms of actin nucleation", there are two proposed mechanisms for Arp2/3-independent actin nucleation: linear tandem and cross-filament. These two mechanisms are not mutually exclusive, because a linear nucleus can be converted to a cross-filament seed by the addition of one or two lateral G-actin monomers. These were both modelled using the parameters listed in Table 1, and compared to experimental measurements of actin polymerisation detected using a pyrene assay^35^.

**Fig. 3:**
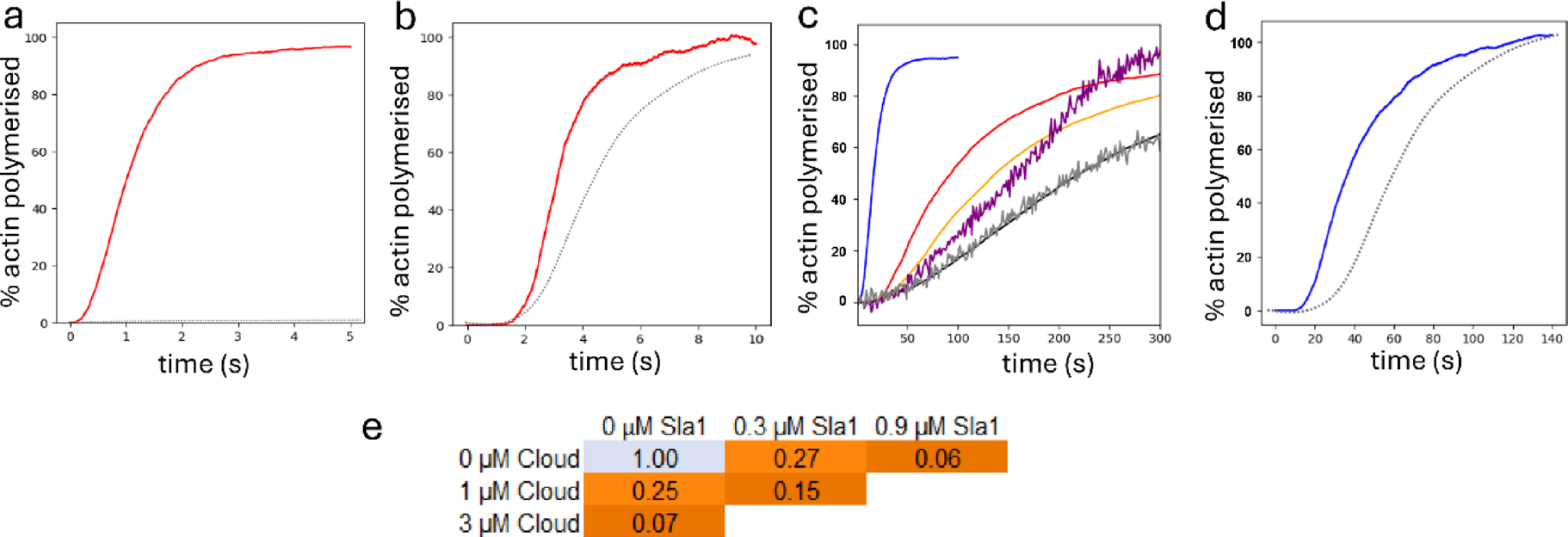
Comparison of modelled actin polymerisation curves to experimental data. (**a**) Cross-filament model (red) in comparison to experimental actin polymerisation rate (black dashed, close to the baseline), using 300 nM Las17 and 5 µM actin. (**b**) Cross-filament model (red) compared to experimental data (black dashed) for the cross-filament nucleator Cobl^28^ with 40 nM Las17 and 2 µM actin. (**c**) Experimental actin only control (gray) and nucleated by Las17 (magenta), for 300 nM Las17 and 5 µM actin, compared to: control actin-only model (black); cross-filament model (blue); longitudinal model (red); and longitudinal model with Las17 dimerization (orange). The experimental rates have been adjusted for the change in buffer from 1xKME to 0.5xKME, as discussed in Supplementary Material. (**d**) Longitudinal model (blue) compared to experimental data for Spire (black dashed), with 500 nM Las17 and 4 µM actin^27^. (**e**) Sensitivity analysis showing the effect of Sla1 concentration and SH3 cloud concentration on nucleation rate. Rate decreases of > 10% are in increasingly darker shades of orange.

Modelling using monomeric Las17 and the cross-filament model produces a calculated polymerisation rate that is many orders of magnitude too fast compared to the experimental pyrene assay (Fig. 3a). Interestingly, the rate matches well to that obtained using the known cross-filament nucleator Cobl (Fig. 3b). Adjustment of parameters (weakening of affinities, or reduced on- and off-rates) was unable to reproduce the experimental rates: it required an actin-Las17 association rate of 0.002 µM^−1^ s^−1^ and dissociation rate of 10^−9^ s^−1^, which are > 10^8^ times smaller than typically observed rates^36^.

By contrast, a longitudinal nucleation mechanism gave calculated polymerisation rates that matched well to experimental data (Fig. 3c), and to data for the longitudinal nucleator Spire (Fig. 3d). Inclusion of SH3 domains into the model (which slow down nucleation by competitive binding to actin binding sites) gave better agreement, demonstrating that SH3 domains can exert considerable control over the overall nucleation rate (Fig. 3e). Allowing self-association of Las17 produced even better agreement with experimental data (Fig. 3c), as discussed below.

The model thus confidently predicts that actin nucleation by Las17 proceeds via a longitudinal mechanism. Subsequent modelling showed that the initial slow longitudinal tandem nucleation can then proceed rapidly into a polymerisation phase by recruiting lateral G-actin monomers. This conversion requires two additional G-actin monomers. In other words, the rate-limiting process in the initiation of polymerisation in vivo is the seeding of the second actin sub-filament, which can be stimulated by a range of effectors, as discussed below. Our model stops at this point. Subsequently, once a short double-stranded actin mother filament has been produced, it can then polymerise to increase in length, and it can also bind Arp2/3 to nucleate branched filaments.

It has been proposed that an alternative source of mother filaments is by breakage of pre-existing strands (possibly by cofilin), and diffusion of these short fragments to sites of new endocytic patches^37^ (the sever, diffuse and trigger model). To test this, we repeated modelling calculations, adding 5 µM short actin filaments at the same time as the arrival of the SLAC complex. The result was a rapid and uncontrolled polymerisation of actin, such that almost all free G-actin was polymerised within 0.8 s (Supplementary Fig. S5). We can therefore be confident that this is not how actin polymerisation is initiated. We do not rule out other additional mechanisms of actin nucleation, although the model indicates that no other mechanisms are required.

### Las17 and the SLAC complex

Las17 arrives at the forming endocytic patch as a complex with its inhibitor Sla1. For *de novo* actin nucleation to begin, Sla1 must dissociate from Las17 and allow Las17’s polyprolines to bind G-actin. The model was used to investigate this.

**Fig. 4:**
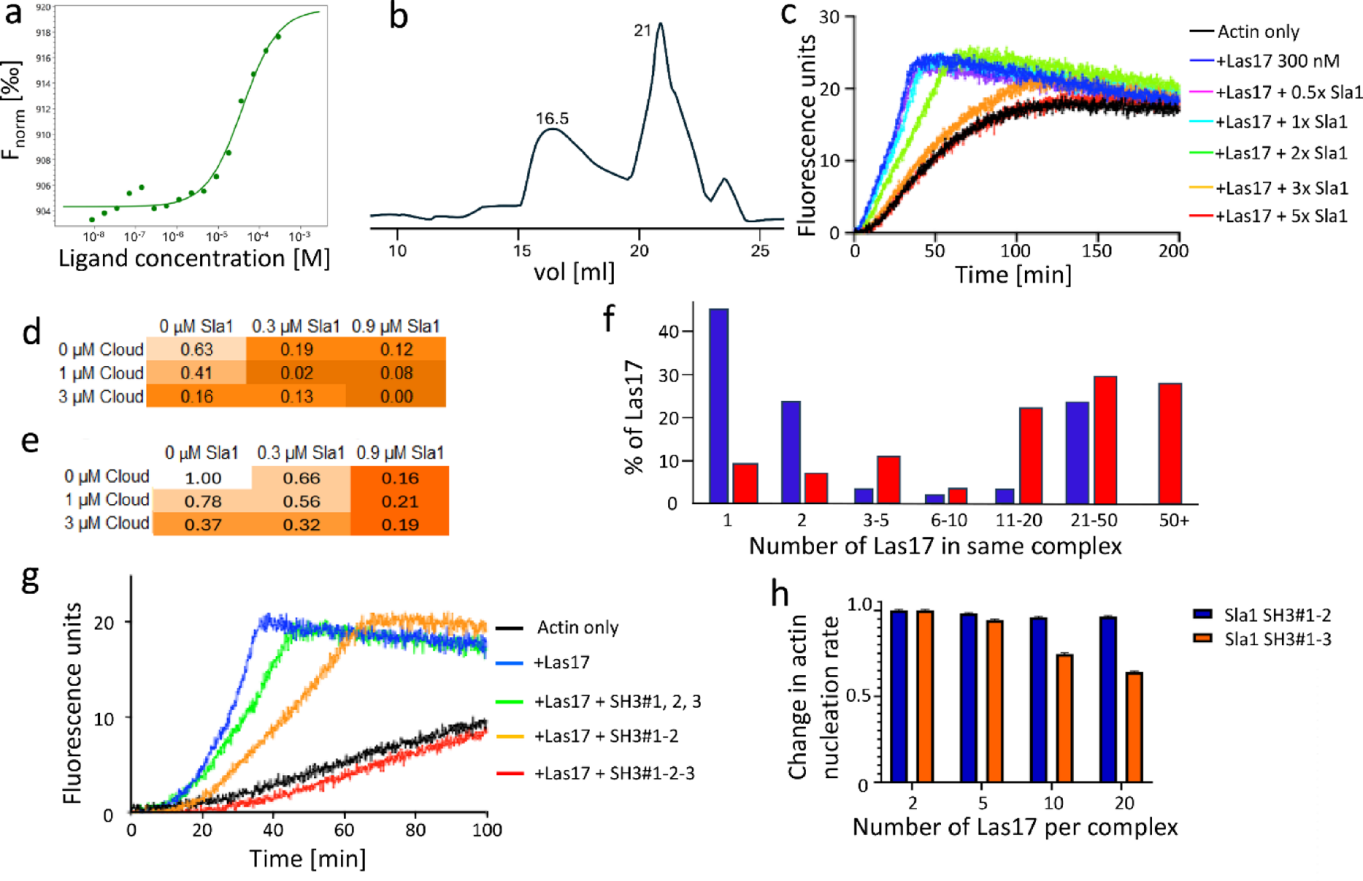
Self-association of Las17. (**a**) Las17 dimerization curve determined by MST using 25 nM SNAP-labelled Las17 (300–633)-His I555D and unlabelled GST-cleaved Las17 (300–422). The I555D mutation was used to prevent actin binding to the WCA region^40^. (**b**) Size exclusion chromatography profile for Las17. A_280_ from a Superdex 200 HR 10/30 column using Las17 (300-422-His) in PBS. The peaks at 16.5 and 21 mL are dimeric and monomeric Las17 respectively. (**c)** Pyrene assays of actin polymerisation. Increasing concentrations of Sla1 SH3 domains 1–3 inhibit Las17-induced actin polymerisation. (**d**) Modelled effect of Sla1 and SH3 cloud on Las17-catalysed actin nucleation rate. (**e**) Same as (d), except keeping Las17 in fixed positions and thus preventing self-association. (**f**) Clustering of Las17 and Sla1 in the model 0.5 s after equilibration. Blue: using a pre-equilibration step. Red: equilibration during the simulation. (**g**) Pyrene assays demonstrating the importance of the Sla1 SH3#3 domain for inhibition of Las17-catalysed actin polymerisation. (**h**) Sla1 SH3#3 inhibits actin nucleation, but only when the SLAC complex is allowed to become large. Error bars show the standard deviation.

There is experimental evidence that Las17 is self-associated, as are other members of the WASP family^38^. Purified Las17(300–422) forms dimers (Fig. 4ab). Sedimentation analysis of the SLAC complex suggested a molecular mass of 822 ± 28 kDa, implying multiple copies of Las17 and Sla1^4^.

In vitro assays show that Sla1 can almost completely inhibit Las17-induced actin polymerisation, at a ratio of Las17:Sla1 of 1:3 (Fig. 4c). The model was able to reproduce this inhibition, with additional cloud SH3 domains causing complete inhibition (Fig. 4d). However, when Las17 was prevented from self-associating, by fixing the monomers at distances too far apart to associate, the model was unable to replicate these observations (Fig. 4e). This shows that Las17 must be associated into a multimeric SLAC complex. The model showed no significant difference on pre-incubation of Las17 and Sla1 (Fig. 4f, Supplementary Fig. S6), suggesting that formation of the complex is rapid. We note similarities between this model and a previous suggestion from the Drubin group^39^.

The model provided further unexpected insights. First, it consistently generated large complexes of Las17 and Sla1, the largest of which contained at least 40 Las17 molecules (Fig. 4f). Second, Las17 contains three polyproline regions, each of which can bind to an SH3 domain, while Sla1 has three SH3 domains. In both molecules, two of these binding regions are very close together, while the third is well separated (Fig. 1). We therefore expected that the SLAC complexes would consist mainly of assemblies of 1:1 pairs. However, examination of the complexes in the model showed that the third SH3 domain of Sla1 often bound to a different Las17 molecule, thus creating a crosslinked complex, which we suggest is the major component of SLAC. The importance of this intermolecular crosslinking by the third Sla1 SH3 (SH3#3) can be seen from the observation that including SH3#3 only strengthens the overall affinity for Las17 2-fold, but greatly increases the ability of Sla1 to inhibit actin nucleation by Las17, both experimentally (Fig. 4g) and within the model (Fig. 4h), because it increases the effective concentration^41^ of Sla1 close to Las17.

### The importance of tandem SH3 domains

A striking feature of endocytosis in yeast is how many endocytic proteins contain SH3 domains, several with tandem domains. An important consequence of tandem binding is avidity. SH3 domains typically bind to polyproline (PP) sequences with affinities in the low µM range. However, if two SH3 domains are connected by a linker, and bind two polyprolines also connected by a linker, then once the first domain is bound, the second domain has a very high effective concentration at its PP target because of the linker, and therefore binds much more rapidly than the first, making the overall binding affinity of the tandem dimer much stronger^42^. This effect operates here: the Sla1 SH3#1-2 pair binds to the PP2/PP3 pair with a dissociation constant of 86 nM^5^.

The increased affinity derives from the increased on-rate of the second domain, as described above. Similar considerations affect the off-rate. The off-rate of one domain from its binding partner is fast, but the overall dissociation of the dimer remains slow, because it requires the unlikely dissociation of both SH3s simultaneously. There is however an important difference. If the system contains another SH3 domain that can compete with a monomer for binding to PP (i.e., with an affinity in the low µM range), then once one SH3 of the tandem dissociates, the competitor can bind instead, which will then lead to rapid loss of the other tandem SH3 (Supplementary Figure S7)^42^. In other words, a single weakly binding SH3 domain can counterintuitively compete off the binding of a much more strongly bound tandem. We therefore hypothesised that the effect of tandem binding would be particularly significant when looking at the displacement of tandems by single competitor domains, i.e. by the SH3 cloud.

**Fig. 5:**
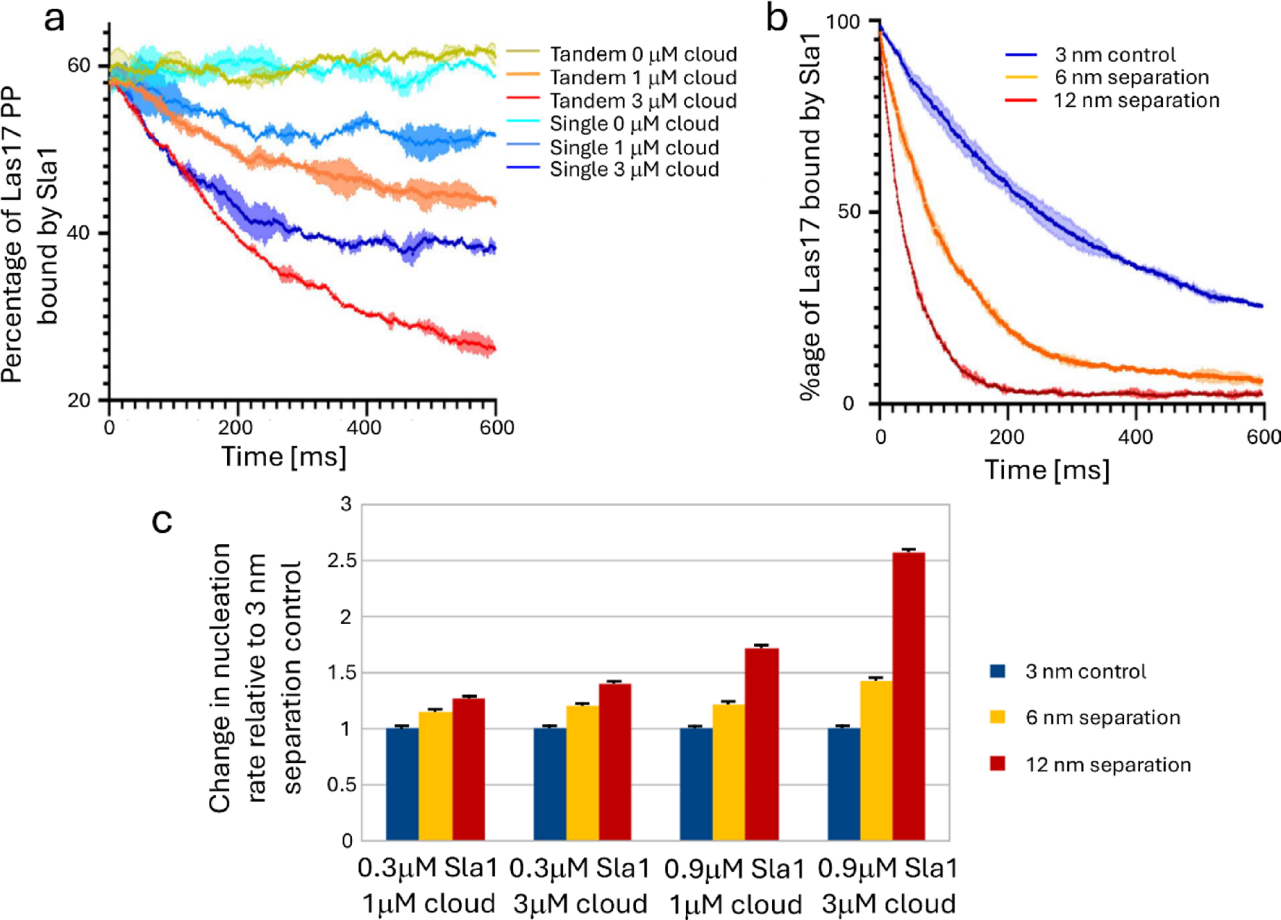
The importance of tandem domains. (**a**) Plotting the percentage of Las17 PP3 tracts bound by Sla1 reveals that the tandem construct (shades of yellow/red) is outcompeted by cloud domains more strongly than single construct Sla1 (shades of blue). (**b**) The degree of Sla1 binding (normalised to the highest binding percentage observed between the simulations) over time following exposure to the SH3 cloud. This value drops as Sla1 is outcompeted by the single SH3s and the rate of this departure is positively correlated to the length of the Sla1 linker separating the first and second SH3 domain. (**c**) Changing the maximal distance separating Sla1-SH3#1 and Sla1-SH3#2 affects the inhibition conferred by Sla1 relative to a 3 nm separation control. Increasing the distance both reduces inhibition of the tandem and increases its susceptibility to cloud SH3 competition. Error bars in the three panels denote standard deviation.

We analysed the effects of tandem binding using the model. When a tandem Sla1 SH3/Las17 PP interaction was replaced by a single interaction of the same overall affinity, the ability of cloud SH3s to help stimulate dissociation of Sla1 was weaker and slower (Fig. 5a). In a second simulation, the length of the linker connecting the two SH3 domains was varied. A longer linker makes the avidity effect weaker, and therefore allows cloud SH3s to displace bound Las17 faster and more effectively (Fig. 5b), and thus increases the overall actin nucleation rate, and makes it more sensitive to cloud SH3 concentration (Fig. 5c). Thus, the effect of cloud SH3s on actin nucleation can be tuned by varying the length of the linkers.

### The trigger for rapid actin polymerisation

Our analysis of actin mother filament nucleation (Sect. “Actin nucleation”) suggests that linear tandem nucleation is a slow and controlled process, creating a single-stranded trimeric F-actin nucleus. Formation of double-stranded F-actin from this nucleus is slow, because it requires an additional actin monomer to be stably incorporated into the second strand of the actin filament (Fig. 2). On the other hand, cross-filament nucleation is rapid, because it provides a mechanism to hold the nascent second strand in place, after which polymerisation can proceed rapidly. Our model suggests that *de novo* actin nucleation in endocytosis occurs by a linear tandem process: an appropriate mechanism because it is essential that actin polymerisation should only start under carefully controlled conditions.

After the SLAC complex arrives at an endocytic site, there is a tightly regulated period of about 10 s before rapid actin polymerisation. During this period, several proteins arrive in a regulated order. We propose that linear tandem nuclei are accumulating during this time, and then during the next 10 s, rapid polymerisation is triggered by a protein that locates a G-actin in place at the side of the initial strand. There are several candidates for such a protein. One is Las17 itself: the C-terminal WH2 domain of Las17 binds actin and stimulates polymerisation^7^. Another possibility is Ysc84 (and/or its paralogue Lsb3)^23^which is one of only of two SH3-containing proteins that is recruited to the endocytic patch between Las17 and Arp2/3, the other being Bzz1^2^. Our analysis of interactions (Fig. 1c) showed that Ysc84 is the only SH3-containing protein of those tested that binds significantly at polyproline site 1a, which consists of PxxP rather than PPPPP and thus is not a binding site for actin. Ysc84 is recruited shortly after the breakdown of the Sla1/Las17 complex, suggesting that the arrival of Ysc84 is stimulated by site 1a becoming available once actin is bound to 1b. Ysc84 also binds actin via its YAB domain, meaning that recruitment of Ysc84 at site 1a could stabilise a laterally bound actin, as previously hypothesised^23^.

**Fig. 6:**
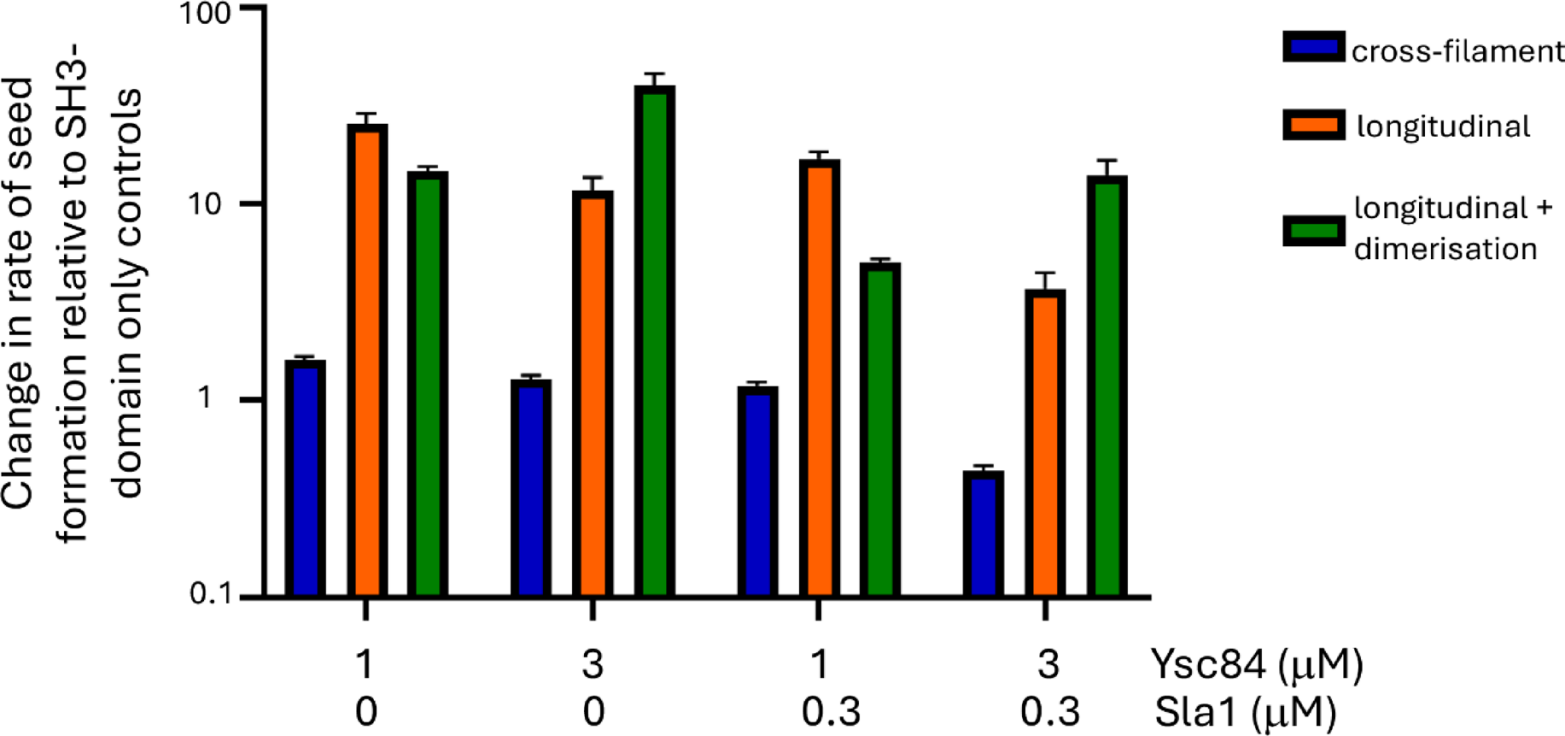
The rate of F-actin seed production compared to the appropriate controls which were simulated using the same protein concentrations, but with Ysc84 lacking the YAB domain. The model was run using three conditions: cross-filament nucleation; longitudinal nucleation with static (monomeric) Las17; and longitudinal nucleation allowing Las17 to move and dimerise. Las17 longitudinal simulations in the presence of full-length Ysc84 produced approximately a 10-fold increase in rate of seed formation (note the log scale for the rate axis: orange or green vs. blue bars). Inclusion of the YAB domain slowed cross-filament nucleation (blue bars), as any positive effects were offset by the reduction in nucleation because of G-actin sequestering by Ysc84. Error bars show standard deviations.

We therefore modelled the effect of inclusion of the YAB domain into the model, allowing actin to bind to Ysc84. The results are shown in Fig. 6, indicating that actin binding to Ysc84 causes approximately a 10-fold increase in the rate of nucleation compared to simulations lacking a YAB domain, either with or without Las17 dimerisation and Sla1. In the absence of Ysc84, nucleation is slow because any G-actin monomers that bind laterally to the tandem nucleus dissociate rapidly. In its presence, laterally bound actin is stabilised, thus converting nucleation into the much faster cross-filament mode. Similar results have been seen experimentally^23,26^. We note that the linker between the 1a binding site and the actin binding site is long, as required for cross-filament nucleation (Supplementary Table S1). Thus, arrival of Ysc84 could act as one possible trigger that begins actin polymerisation. We explore this idea further in the next section.

### Modelling membrane interactions

The calculations described thus far have been “in solution”. The model can be readily modified to simulate a membrane surface, and thus begin to model a realistic endocytic patch. Details are in Supplementary, but briefly: One surface of the box was replaced by an elastic membrane, such that an agent attempting to move beyond the membrane is reflected. Proteins known to bind to the membrane were restricted to within 80 nm of the surface, with Las17 restricted to within 10 nm. Consequently, their effective concentrations increased dramatically. A 200 × 200 nm square patch was defined on the membrane surface, with Las17 and Sla1 restricted to the area of the patch. Any Sla1 agents not bound to Las17 were removed from the simulation using a definable probability aimed at replicating the effect of Sla1 being sequestered by cargo^43,44^. Finally, to reproduce in vivo behaviour, the simulation was run for 10 s with only actin, Las17, Sla1 and the SH3 cloud present, after which Ysc84 was added, to reflect the observed arrival of Ysc84 at this point. Bzz1 was then added at 15 s, again to reflect observations.

Because the concentrations of Las17 and Sla1 close to the patch were greater than in the in vitro simulations, the actin nucleation rate was slower. The SH3 cloud had the expected effect of stimulating breakdown of the SLAC complex by competing with Sla1 for the Las17 PP sequences; it also slowed down filament nucleation by blocking actin binding (Supplementary Fig S8).

**Fig. 7:**
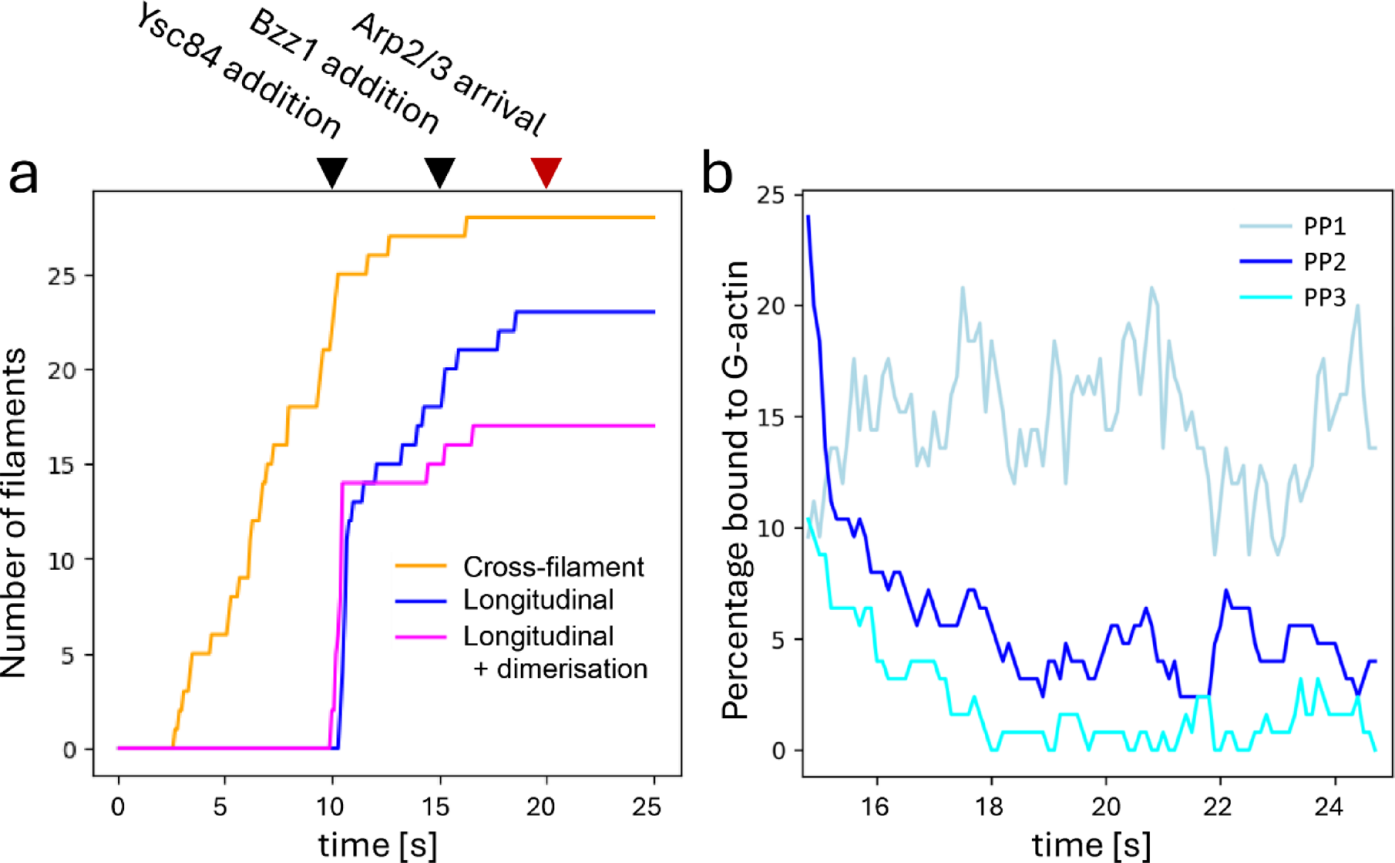
Impact of Ysc84 and Bzz1 on the endocytic patch. (**a**) Number of F-actin filaments after the arrival of SLAC complex. Ysc84 is added at 10 s, and Bzz1 at 15 s. For the three colours, see the legend to Fig. 6. (**b**) The percentage of Las17 tracts bound to actin following the recruitment of Bzz1. There is a significant decrease in the percentage of PP2 and PP3 tracts being bound to actin due to competition with the increasing number of SH3 domains.

Cross-filament nucleation produces a gradual increase in F-actin filaments, whereas longitudinal nucleation produces none, until Ysc84 is added at 10 s, when there is a sudden sharp burst in filament nucleation (Fig. 7a). This is the effect expected based on the earlier simulations. Addition of Bzz1 at 15 s shuts down production of new filaments, because its tandem SH3 domains bind strongly to Las17 and displace actin from the PP2 and PP3 sites (Fig. 7b). From this point on, actin polymerises onto existing filaments rather than forming new mother filaments.

The model thus suggests that Bzz1 has two important functions. It displaces actin nuclei from Las17 and therefore stimulates the growth of linear actin filaments; it also binds to Las17, and prevents Las17 generating new actin nuclei. It therefore directs G-actin monomers into elongating linear filaments, rather than initiating new mother filaments.

### Phosphorylation of Las17 helps to weaken binding to Sla1

The results presented here provide a compelling case that the model works. However, it is a minimalist representation of reality, and there must be much more involved than discussed here, in particular with regards to the regulation of endocytosis. As one example, we note that the third SH3 domain of Sla1 is known to bind to ubiquitin^45^. Binding of ubiquitin to SH3#3 may contribute to the dissociation of Sla1 from Las17, but is clearly not essential. Secondly, there is strong evidence supporting phosphorylation at a number of sites close to the three N-terminal polyproline sequences, including T332, T334 and S337 (between PP1 and PP2), T363 (downstream of PP2) and T380 (upstream of PP3)^46–48^. NMR titrations of phosphorylated peptides with Sla1 SH3#1-2 demonstrate a marked reduction in affinity, suggesting that dissociation of Sla1 from Las17 is enhanced by Las17 phosphorylation (Supplementary Figure S9). Thus, phosphorylation of Las17 is likely to contribute to regulation of the start of endocytosis.

## Discussion

### A proposed outline of initiation of actin polymerisation in early endocytosis

Endocytosis is initiated when cargo binding sites are revealed at the plasma membrane, which stimulates relocation of the SLAC complex to the membrane. SLAC is a large complex containing multiple copies of both Sla1 and Las17, most likely held together by the binding of Sla1 SH3#1 and SH3#2 to Las17, and SH3#3 either to the same Las17 or to a different Las17 molecule, thereby creating a multimeric crosslinked assembly. Las17 contains 8 PP stretches, each containing the sequence PPPPP. There is a further PxxP sequence upstream of PP1, which has significant binding only to Ysc84. PP1, PP2 and PP3 bind competitively to G-actin and SH3 domains. The binding of PP1, PP2 and PP3 to G-actin is strengthened by the presence of two arginines upstream of each PP^5, ^which are present only in these three sequences.

The binding of Sla1 to the cargo and/or membrane weakens its binding to Las17, allowing it to dissociate^5^. This is promoted by the SH3 cloud, which binds to transiently exposed PP sites on Las17 and reduces the avidity of the tandem SH3 domains of Sla1. This allows G-actin to bind to PP1-3 of Las17, forming a trimeric tandem linear nucleus. Polymerisation to F-actin from this nucleus is slow: the trimeric nucleus is effectively a “seed in waiting”. Rapid F-actin polymerisation requires the stable binding of a lateral G-actin monomer, to form a cross-filament seed. This can probably be provided in several ways – for example, binding of the C-terminal WH2 domain of Las17 to G-actin^7^or binding of Ysc84 to PP1a. We suggest that Ysc84 binding to PP1a is stimulated by the removal of Sla1 from PP1b, thereby opening up the binding site at PP1a. We note that this mechanism of nucleation provides a high level of control over the timing of the burst of actin polymerisation, as clearly occurs in vivo.

**Fig. 8:**
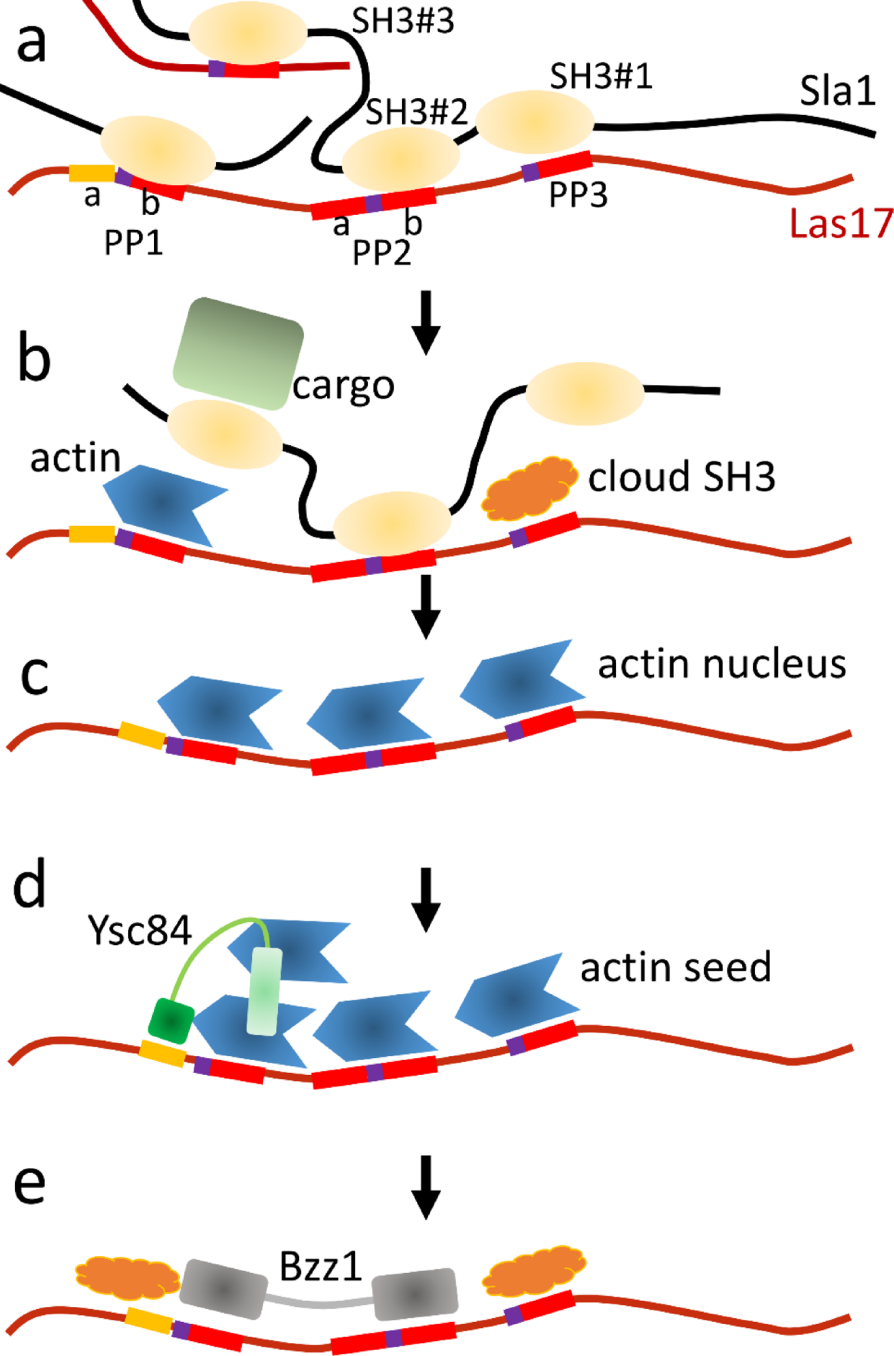
A model for early endocytosis. (**a**) The SLAC complex arrives at the developing endocytic patch. It contains multiple Sla1 and Las17, with most polyproline (PP) sites on Las17 being bound to SH3 domains of Sla1. (**b**) Sla1 binds to cargo. Its dissociation from Las17 is promoted by cloud SH3, which can bind to PP when Sla1 detaches, as can G-actin. (**c**) Once Sla1 has detached, the PP sites can bind G-actin, thus forming a trimeric longitudinal nucleus. (**d**) This exposes a PxxP site on Las17 (yellow), which binds to the SH3 domain of Ysc84. Ysc84 also has a YAB domain (light green) which binds G-actin, and so helps to create an F-actin cross-filament seed, which can then polymerise, detach from Las17, and start forming branches with Arp2/3. (**e**) Bzz1 (which has tandem SH3 domains) can then bind to Las17 and prevent further nucleation of new mother filaments.

The binding of the cross-filament G-actin stimulates a burst of actin polymerisation. Formation of F-actin filaments causes actin to dissociate from Las17, exposing PP sites 1 through 3, which are buried by SH3 proteins including the tandem domains of Bzz1. By this point, Sla1 is no longer available, because it has been recruited to the centre of the endocytic patch^18,43,49^. We propose that binding of Bzz1 to Las17 blocks further G-actin nuclei forming, and therefore shuts down further nucleation of new mother filaments (Fig. 7), encouraging the formation of longer F-actin filaments. These filaments can also bind to Arp2/3 and nucleate branched filaments. The lengths of the filaments, and thus the density of branches, are regulated by Ysc84 and Bzz1, which together create an “activity window” for Las17 nucleation (Fig. 8).

### Some implications

*De novo* actin nucleators fall into two classes: linear tandem nucleators and cross-filament nucleators (Fig. 2). Las17 is a linear nucleator, like Spire and JMY. Nucleation is therefore slow and controlled, with rapid polymerisation being stimulated by addition of a cross-filament G-actin. We contrast this to the cross-filament nucleator Cobl, which functions as a dominant nucleator (possibly with aid from the homologue Cobl-like) at dendritic branch induction sites^50^and is thus faster but less controlled.

Endocytosis requires many different SH3-containing proteins. Three of these (Las17, Ysc84 and Bzz1) have clearly defined roles. We have treated the others as an “SH3 cloud” and shown that they have an important function in regulating interactions between Las17, Sla1 and Bzz1. The cloud facilitates the breakdown of the high avidity interaction between Sla1 and Las17, controls the dissociation of Sla1, and regulates the binding of G-actin to Las17. Later in endocytosis, it regulates the binding of Bzz1 and the shutdown of nucleation of new mother filaments, with these roles requiring newly arrived SH3 domains such as those in the type I myosins Myo3 and Myo5^2^. The affinities of different cloud members do not seem to be critical.

The model may explain the enigmatic function of Ldb17 (Dip1 in *Schizosaccharomyces pombe*, SPIN90 in mammals). Dip1 arrives at the endocytic patch around 10 s after Sla1^51,52^ (and thus roughly the same time as Bzz1), and about 10 s before Arp2/3. Deletion of Dip1 makes actin polymerisation late and erratic^51^. In vitro, it binds to Arp2/3 and stimulates the growth of actin filaments in the absence of preformed filaments^53^. Ldb17 and SPIN90 (but not Dip1) have a C-terminal polyproline region, which binds to the SH3 domain of Bzz1^52^. It is therefore possible that Ldb17 shields the SH3 domain(s) of Bzz1 to prevent it from interacting too soon with Las17; and that Ldb17 is subsequently displaced from Bzz1, helping to organise the growth of linear filaments and the binding of Arp2/3.

This work has modelled endocytosis in *S. cerevisiae*. However, we anticipate that the events described here will be relevant elsewhere. For example, the yeast Ste20 kinase regulator Bem1 also interacts through a tandem pair of SH3 domains^54^. Furthermore, three proteins (Las17, leiomodin, and SCAR/WAVE) may all bind actin directly through a polyproline tract^55,56^. Nucleation via direct actin-binding polyproline regions occurs elsewhere: for example, actin nucleation via the WAVE polyproline binding region was recently described^56^. Las17 belongs to the WASP family of proteins that includes WASP and WAVE, which have been linked to both human hematopoietic malignancies and immune deficiencies^57,58^. Las17 shares a high degree of structural and functional similarity to its human WASp family paralogues^59^while the tandem SH3 domains of Nck bear a striking resemblance to Sla1. The proposals here could therefore be readily extended to animals.

## Materials and methods

*Dot blots* Membrane dot blots using 12-mer peptides from Las17 covering the sequence 300–536 were purchased from Intavis Celluspots, and were incubated with GST-fused SH3 domains as described^26^. Membranes were probed with HRP-tagged anti-GST and detected using ECL chemiluminescence (Thermo Fisher Scientific).

*Pyrene assay for actin polymerisation* Pyrene assays were carried out in polystyrene wells (ProxiPlate, PerkinElmer) and observed using a Cary Eclipse fluorimeter (emission 385 nm, slit 10 nm round; excitation 364 nm, slit 20 nm). A multichannel pipette was used to simultaneously transfer Las17 and KME (10 mM Tris-HCl, pH 8.0, 50 mM KCl, 1 mM MgCl_2,_ 1 mM EGTA, made up to 0.5x KME) to each assay well. The wells were mixed and checked for air bubbles before being placed into the fluorimeter. Each assay well (prior to the addition of Las17 and KME) contained 3 µM G-actin, 0.3 µM pyrene-actin, 0.2 mM ATP, 0.5 mM DTT, and G-buffer (2 mM Tris pH 8.0, 0.2 mM CaCl_2_, 0.5 mM DTT and 0.2 mM ATP (the two latter added < 1 h before use)). Actin and pyrene-labelled actin were produced following the protocols detailed in^35,60^ respectively.

*Las17 dimerisation measurement.* Las17 was purified as described in^5^. Las17 was SNAP tagged using the Monolith 2nd Generation Snap-Tag RED Protein Labelling Kit provided by NanoTemper. Free dye was separated from the labelled protein using a buffer exchange column. The absorbance at 280 nm (protein) was compared against the absorbance at 647 nm (dye) to calculate labelling efficiency with a target efficiency value above 80%. Dimerisation was measured using microscale thermophoresis as described^5^.

*Predicted SH3 binding sites.* The spot array data of Fig. 1b were combined with a screening study^12^ to obtain preliminary predictions. Further details are given in Supplementary Material.

*Peptide titrations by NMR.* Peptides were synthesized by Genscript (UK). The protein was expressed in *E. coli* with^15^N-labelled M9 medium, as a GST tagged protein, purified and cleaved using Prescission protease as described^5^. NMR experiments were carried out on a Bruker DRX-600 with cryoprobe, and shift changes were analysed using Felix NMR 2007 (Felix NMR, Inc., San Diego CA, www.felixnmr.com).

*Ordinary differential equation (ODE) modelling.* The equations were modelled using python 3.7 (python.org) and integrated numerically. ODE simulations for comparisons with ABM outputs were produced by calculating changes in populations of complexes over discrete time steps. Forward rates were calculated as *k*_on_[protein 1][protein 2] while back rates were calculated as *k*_off_[protein complex]. Concentrations of simulated elements were updated following each 100 µs timestep.

*Modelling* A detailed description of the model can be found in Supplementary Material (Supplementary Figures S10-S15; Supplementary Tables S2-S6). The model (in C) together with example XML setup files are available on Github (https://github.com/LewPhan1/Las17ABM.git).

## Supplementary Information

Below is the link to the electronic supplementary material.

Supplementary Material 1

## Data Availability

The data reported here are available from the corresponding author upon reasonable request. NMR shift changes (Supplementary Fig S9) are deposited at BMRB (https://bmrb.io) as BMRbig129.
