# Supplementary material for: Agent-based modelling of the early stages of actin polymerisation required to drive endocytosis in Saccharomyces cerevisiae
Source: Sci Rep. 2025 Aug 7;15:28951. doi: 10.1038/s41598-025-14248-w (PMC12332068; doi:10.1038/s41598-025-14248-w)
Supplement: Supplementary file 1 — Supplementary Material 1 [file 41598_2025_14248_MOESM1_ESM.docx]

Agent-based modelling defines the early stages of endocytosis in *Saccharomyces cerevisiae*

Lewis P. Hancock, Ellen G. Allwood, John Palmer, Kathryn R. Ayscough and Mike P. Williamson

**Supplementary Material**

This Supplementary Material contains: Reasoning behind the binding locations shown in Figure 1c; Table S1 (linker lengths in different actin nucleators); Figure showing the NMR spectrum of Sla1 SH3#1; Discussion of how model parameters were determined; Buffer conditions; Sensitivity analysis of the model; Modelling the addition of F-actin filaments as a source of new mother filaments; Figure showing effect of omitting the pre-equilibration of Las17 and Sla1; Competition of tandem binding by a monomer; Figure modelling effect of cloud SH3 on F-actin nucleation; binding of phosphorylated peptides to Las17; A detailed description of the model (the model itself is deposited on Github).

Reasoning behind the binding locations shown in Figure 1c

Spot array data (Fig 1b) were first used to link SH3 domains to regions of Las17. All core SH3-binding sites of the appropriate class were identified and compared against a large peptide screening study taken from the literature (Tonikian et al. 2009). These comparisons were each given a score using the following criteria: If the binding site residue matches the most frequently observed peptide residue at that position: +4 points. If the binding site residue was identified at that peptide position, although it was not the most frequently observed: +2 points. If the binding site residue was never observed at that peptide position, although one or more residues with a shared biophysical property were (e.g., hydrophobic, same charge type, etc.): -2 points. If the binding site residue was never observed at that peptide position, and neither were any similar residues with shared biophysical properties: -4 points. The scores were then weighted by the importance of that residue position as determined by the screen results. Highly important positions were given a 2x modifier whilst important positions were given a 0.5x modifier. Combining the residue scores then generated an SH3 preference prediction for each site binding to Las17. These predictions were used to estimate which sites they are likely to occupy, rather than to compare SH3 domains. There is an additional polyproline sequence (six consecutive prolines) at residues 185-190, shown in Figure 1aii. It has no arginines nearby and is not required for binding actin, so it was not included in this analysis.

| \| **Tandem nucleator** \| **Length between centre of binding sites 1b and 2b (residues)** \| **Length between centre of binding sites 2b and 3 (residues)** \| **Length between centre of binding sites 3 and 4 (residues)** \| \| --- \| --- \| --- \| --- \| \| Las17 300-422  (Budding Yeast) \| 29.5 ^(1)^ \| 33.5 ^(1)^ \|  \| \| Spire  (Human, Spir1) \| 40 ^(1)^ \| 29.5 ^(1)^ \| 31.5 ^(1)^ \| \| JMY  (Human) \| 28 ^(2)^ \| 31 ^(2)^ \|  \| \| Cobl  (Human, cordon-bleu) \| 40 ^(1)^ \| 88 ^(1)^ \|  \| |
| --- | --- | --- | --- | --- | --- | --- | --- | --- | --- | --- | --- | --- | --- | --- | --- | --- | --- | --- | --- | --- |
| **Table S1. Residue spacing between the centre of actin-binding motifs in tandem nucleators**. The number of residues separating the centre of each actin-binding motif ^1^(Bateman et al. 2023); ^2^(Zuchero et al. 2009). This reveals that the nucleating tracts of Las17 are separated by roughly the same number of residues as the nucleating WH2 domains of Spire and JMY. Also indicated is that the cross-filament nucleator Cobl employs a much larger separation (88 residues) than Las17, suggesting that cross-filament nucleation requires a larger separation between binding sites. |

**
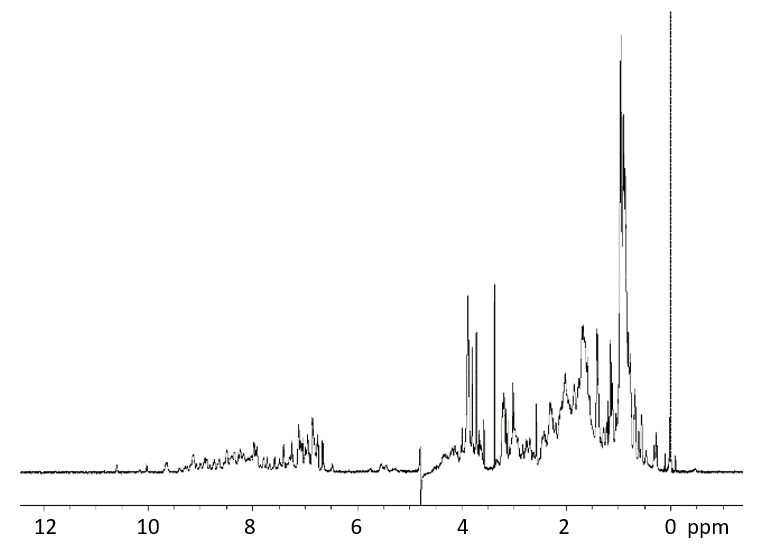
**

**Figure S1.** 1D NMR spectrum of Sla1 SH3#1, indicating correct folding, indicated by sharp signals outside the random coil range.

Discussion of how model parameters were determined

The 300-422 residue region that constitutes the Las17 PP agents includes five binding sites termed PP1a, PP1b, PP2a, PP2b and PP3. PP1a represents an SH3-binding PxxP core motif between residues 314 to 317 whilst PP1b-PP3 represent the four polyproline tracts – known to bind both actin and SH3 domains – of this peptide (Fig 1c).

PP1b can only bind strongly to SH3 domains and not actin agents. This is because it is the only tract in the 300-422 region without an N-terminal arginine pair and is also spaced inconsistently (Table S1). PP2a is directly N-terminal to the PP2b RRGPAPPPPP tract and viewing this region in PyMOL reveals that this separation would be too small for both tracts to bind actin (Hancock et al. 2025). PP2 and PP3 also share a conserved RRGPAPPPPP motif that is hypothesised to be the strongest actin-binding site in the Las17 polyproline region. Furthermore, the separation between PP1b and PP2b is roughly consistent with the separation between PP2b and PP3 (Table S1) heavily suggesting PP2b to be involved with actin binding rather than PP2a. This can easily be managed within the model by giving PP2a a vanishingly small affinity for actin.

Section 3.1 notes that some parameters were obtained by comparing to ODE simulations. As an example, **Figure S2** shows the fitting used to derive the affinity of Sla1-SH3#2 for Las17 polyproline site 2b.


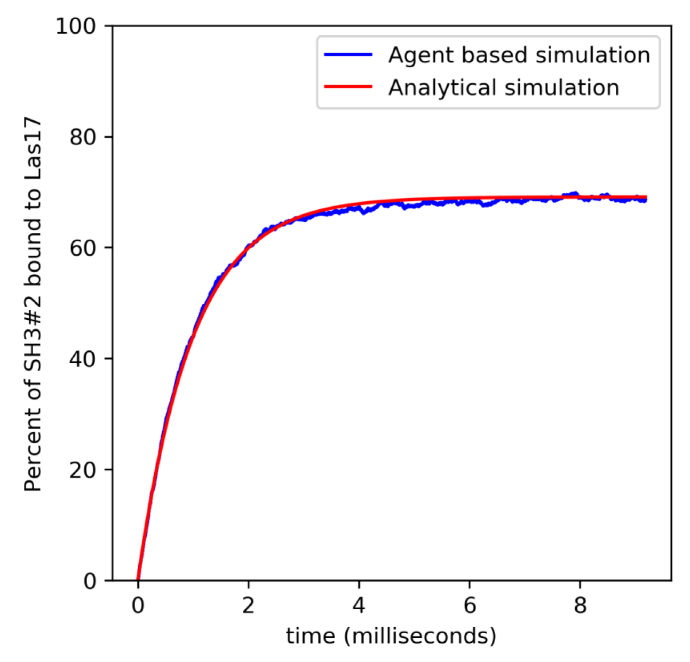


**Figure S2. Using modelling to estimate the affinity of Sla1-SH3#2.** The *k*_on_ and *k*_off_ rates for Sla1-SH3#1-2 were simulated using a simple ODE model to produce an analytical binding curve (red). This was then compared to model output (blue), where SH3#1 affinity was fixed at 7.5 μM and SH3#2 affinity was varied. The SH3#2 affinity required to produce best agreement was 15-20 μM.

Other affinities were determined experimentally. Many of these determinations have been published already (Hancock et al, 2025). In **Figure S3** we show BLI measurements of the affinity of the Ysc84 SH3 domain to Las17(300-422).


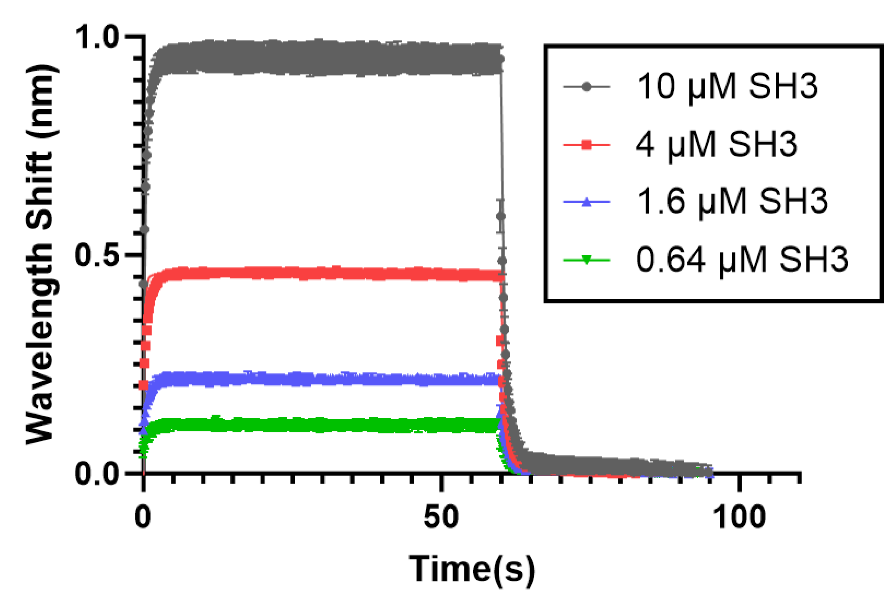


**Figure S3.** BLI binding curves for the binding of Ysc84 SH3 domain to Las17(300-422), using 7.5 μg/ml Las17(300-422) and the SH3 concentrations shown. Ni-NTA probes pre-incubated with Las17-His were placed in Ysc84-containing buffer at t = 0 and moved into a buffer-only well at t = 60. Curve fitting was undertaken using the “Association then dissociation” linear regression fit in GraphPad Prism 10.0.2. Assays were carried out in duplicate. Both the binding site analysis and the spot array data (Figure 1) predicted that Ysc84 SH3 binds more strongly to PP1a than to any of the other PP sequences. Accordingly, the measured affinity (2.2 μM) was assigned to PP1a binding, while the other sites were assigned an affinity half as strong.

Buffer composition (Figure 3)

Experimental investigations of actin nucleation and polymerisation can be carried out in a range of buffers. As noted in the Methods section, a common buffer is G-buffer, which has very low salt (2 mM Tris pH 8.0, 0.2 mM CaCl_2_, 0.5 mM DTT and 0.2 mM ATP), to prevent spontaneous actin polymerisation. A more physiological buffer is KME (10 mM Tris-HCl, pH 8.0, 50 mM KCl, 1 mM MgCl_2,_ 1 mM EGTA). This is still low salt compared to physiological conditions, but generates significant nucleation and polymerisation in the absence of nucleators, and is widely used in studies of actin nucleation. However, it creates problems in studying weak nucleators such as Las17, because the ‘background’ nucleation rate is already high. We have therefore typically used 0.5 x KME in our experimental assays (Allwood et al. 2016), to give much slower spontaneous nucleation, and thus a clearer distinction between catalysed and uncatalyzed nucleation. However, we decided to parametrise the model for 1 x KME, this being a more commonly used set of conditions. The only place where this becomes a problem is in the data for Figure 3c, where we wish to compare the polymerisation rate of actin ± Las17 experimentally *vs* the model. We reasoned that the ratio of the polymerisation rate in the presence of Las17 compared to the rate in the absence of Las17 will be similar in 1 x KME and 0.5 x KME: if anything, it will increase by a larger ratio in 0.5 x KME. Therefore to generate the results shown in Figure 3c, we calculated the uncatalyzed actin polymerisation rate using the model at 1 x KME (black); and then scaled up the experimental rates (gray and magenta lines in Figure 3c) so that the experimental and modelled uncatalyzed polymerisation rates matched (gray and black, respectively), as a reasonable way of estimating an upper limit for the Las17-catalyzed rate in 1 x KME (Figure 3c, magenta).

Sensitivity analysis of the model

*Model reproducibility*

The model was typically run with 0.3 μM Las17 (1000 molecules), 1.2 μM Sla1 (4000 molecules) (4x more Sla1 than Las17, in line with expected concentrations (Ho et al. 2018)), and 5 μM actin monomers (16667 molecules), which took approximately 24 hours on our home-built computer for a 3-second simulation. We expected that the large number of parallel calculations would provide statistical reliability without the need to repeat the calculation multiple times. Repeated simulations under a variety of conditions gave nucleation rates that agreed within 5%, giving us confidence in their reproducibility.

*Changes to rates and affinities*

All binding events were defined by an on-rate and an off-rate, binding affinity being determined by their ratio. As an example we describe here calculations of actin nucleation and polymerisation rates, as a result of Las17 binding. **Figure S4** shows the result of varying either the values of *k*_on_ and *k*_off_, keeping their ratio constant (different rows); or varying the ratio *k*_off_/ *k*_on_ (Table 1, footnote e) to alter the value of *K*_d_ (different columns). The outcomes were as expected. A doubling in rates (top rows) leads to a 2-4-fold increase in actin nucleation and polymerisation rates, while a 2-fold increase in affinity (left columns) has a similar effect. The two effects are roughly additive. **Figure S4c** shows the effect of altering the cooperativity between adjacent actin binding sites (the change in affinity at one site when the second site is occupied). This was set to 10 based on literature (Rasson et al. 2015). The value has very little effect on nucleation rate.


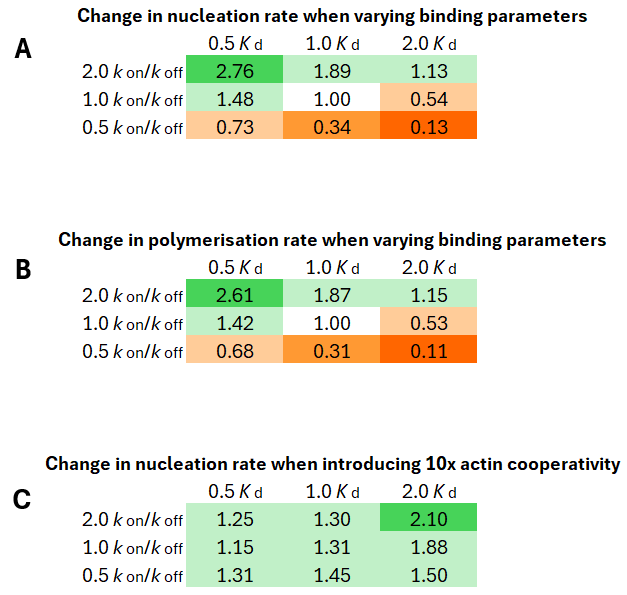


**Figure S4. Actin rate sensitivity analysis.** (**A-B**) The change in indicated rates (nucleation or polymerisation) after applying the indicated *k*_on_/*k*_off_ and/or *K*_d_ change. The rate produced when using the default values given in Table 1 (i.e., the 1x values) serves as the control rate. The displayed numbers are calculated as the fold difference in rate compared to the control. Results are coloured progressively more orange the larger the reduction in rate, and progressively more green the larger the increase in rate. **C**) The change in rate following the introduction of 10x actin cooperativity (i.e., a 10x affinity increase when an actin agent is bound to an adjacent Las17 binding site).

We also tested the effect on the nucleation rate of varying Sla1 and cloud SH3 rates, concentrations, and affinities. Changes to *k*_on_ and *k*_off_ had little effect, while increases in affinity (of Sla1 or SH3 to Las17), or increases in concentration of Sla1 or SH3, reduced the actin nucleation rate, such that a doubling in concentration produced about a 20% reduction in nucleation rate. Changing the diffusion rates had little effect on actin nucleation and polymerisation. We conclude that the model behaves as expected, and that errors in parameter values will have relatively small and predictable effects on actin nucleation and polymerisation rates.

Modelling the effect of F-actin filaments as a source of mother filaments

Section 3.2 presents results demonstrating that new actin mother filaments are nucleated by longitudinal tandem nucleation. An alternative proposal (Chen and Pollard 2013) is that new mother filaments are generated by severing of existing filaments to give short fragments, which then diffuse to new sites of endocytosis. This proposal was tested using the model. The model was initiated using 5 μM G-actin, 0.3 μM Las17 and 0.9 μM Sla1 as normal, except that (following the proposal of Cen & Pollard 2013) 5 μM short actin filaments were also added. The result is shown in Figure S5, indicating virtually all free G-actin becoming polymerised onto the added filaments within 1 s. The experimental result is reported in Figure 5c, showing that polymerisation typically takes several hundred seconds. We therefore conclude that the sever, diffuse and trigger proposal does not match experimental observations.


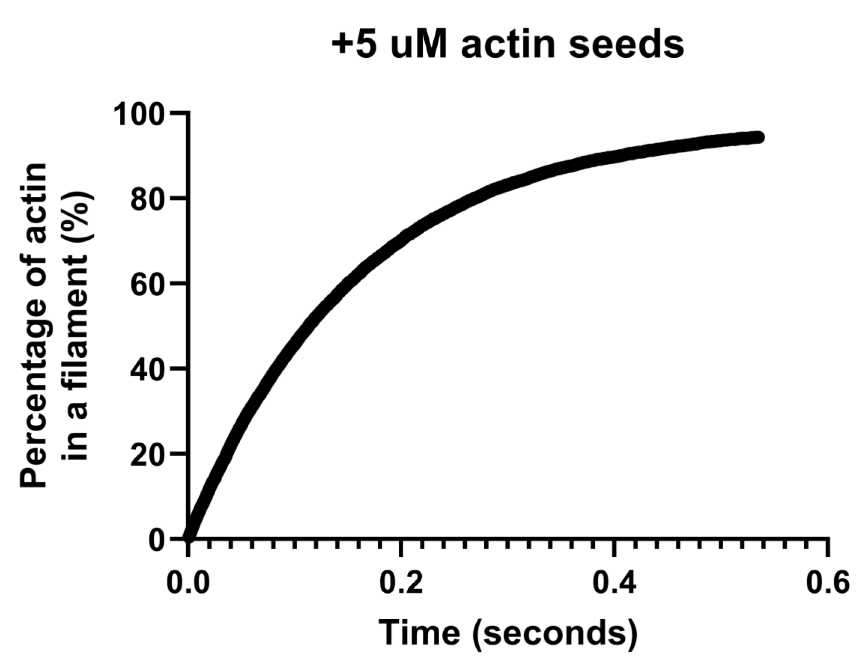


**Figure S5.** Modelled actin polymerisation stimulated by addition of severed actin filaments.


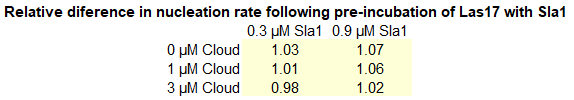


**Figure S6**. The effect of omitting the pre-equilibration of Las17 and Sla1. The difference in values (result X/result Y) when comparing the nucleation rates without Sla1-Las17 equilibration against the nucleation rates with Sla1-Las17 equilibration. Comparisons that gave a <10% difference are coloured in yellow. As can be observed in the figure, no comparison exceeded this 10% threshold indicating no significant difference between the results.


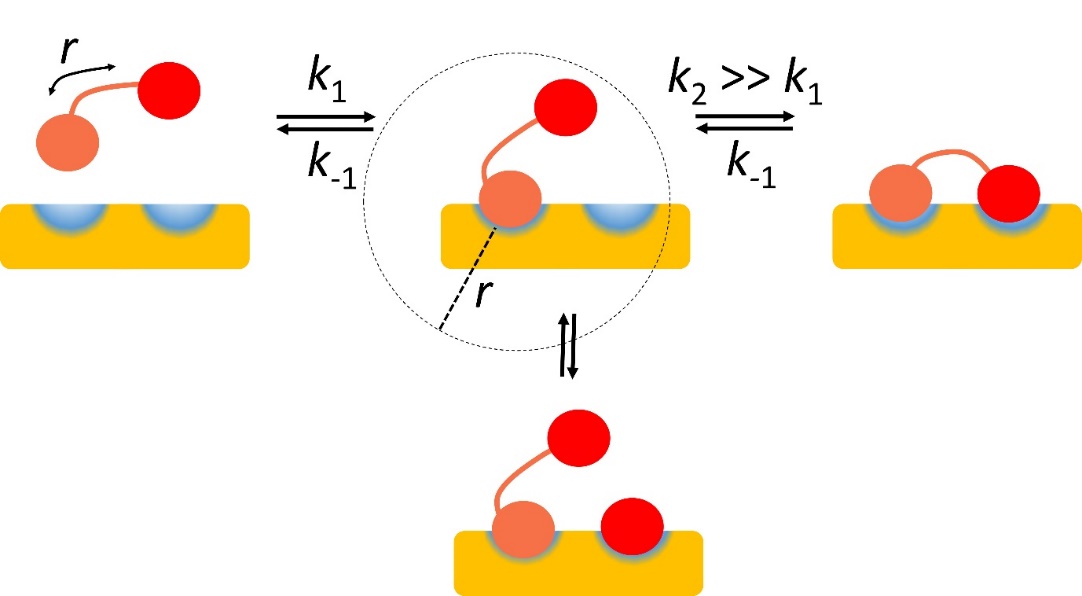


**Figure S7.** Competition of tandem binding by a monomer. The tandem PP sites are in yellow/blue, and the SH3 domains are red and salmon circles. Binding of the first SH3 domain has an on-rate *k*_1_ and an off-rate *k*_-1_, giving an overall affinity for a single domain of *K*_d_ = *k*_-1_/*k*_1_. However, once the first domain has bound, the second is tethered within a sphere of radius *r* (the length of the linker), and therefore has a very high effective concentration. The on-rate for binding of the second domain is therefore much faster, leading to a much stronger overall affinity for tandem binding. Similarly, the off-rates for dissociation of each single domain are roughly equal and are not affected by the tandem nature of the binding. However the off-rate for the tandem as a whole is much slower, because it requires both domains to dissociate at the same time. Counterintuitively, an equivalent monomeric domain that binds in the space left by the temporary dissociation of one monomer (bottom) will prevent its rebinding and will therefore lead to ready dissociation of the tandem dimer, even though its overall affinity is much weaker. Based on (Williamson 2023).


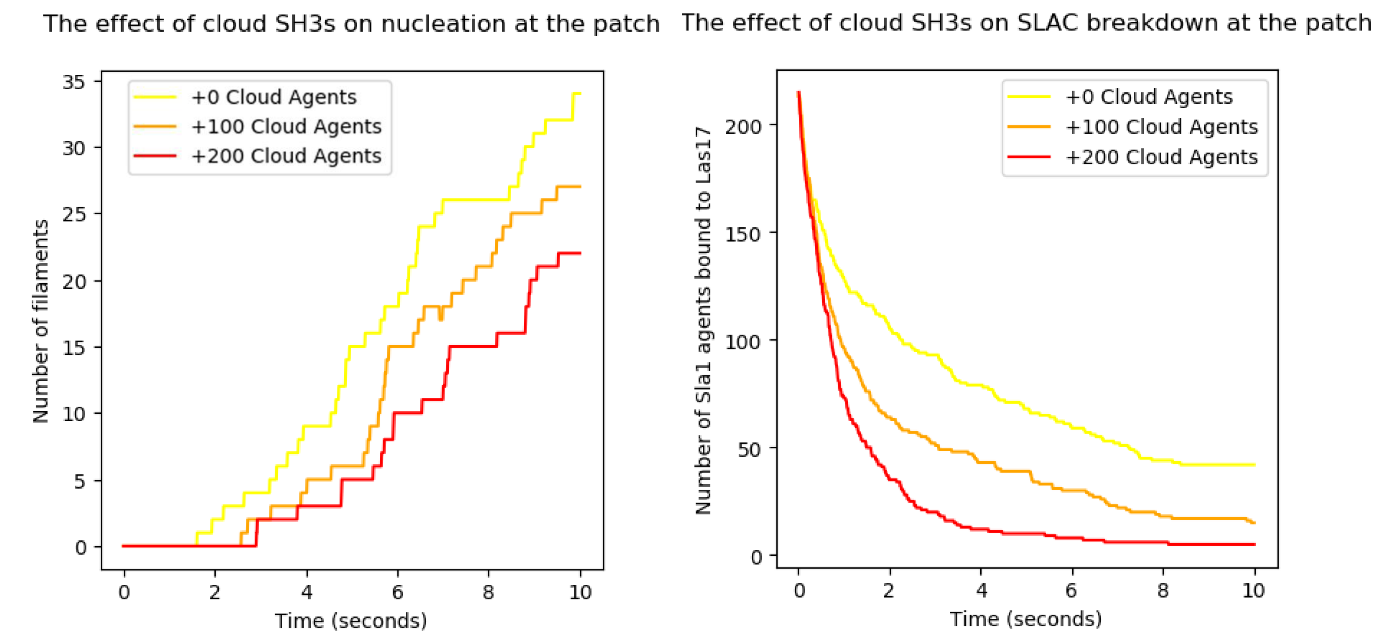


**Figure S8**. The effect of cloud SH3 on F-actin nucleation. All simulations were run for 10 seconds. Las17 and Sla1 were dynamically pre-equilibrated for one second prior to commencing the in vivo simulation (t = 0) to emulate arrival of the SLAC complex to the endocytic patch. (**left**) The nucleation of actin was adversely affected by the number of cloud agents. This figure shows the number of filaments produced over time in the presence of varying levels of cloud SH3s. (**right**) However, increasing the cloud concentration greatly facilitated the loss of Sla1 from Las17 and thereby facilitated breakdown of the SLAC complex.


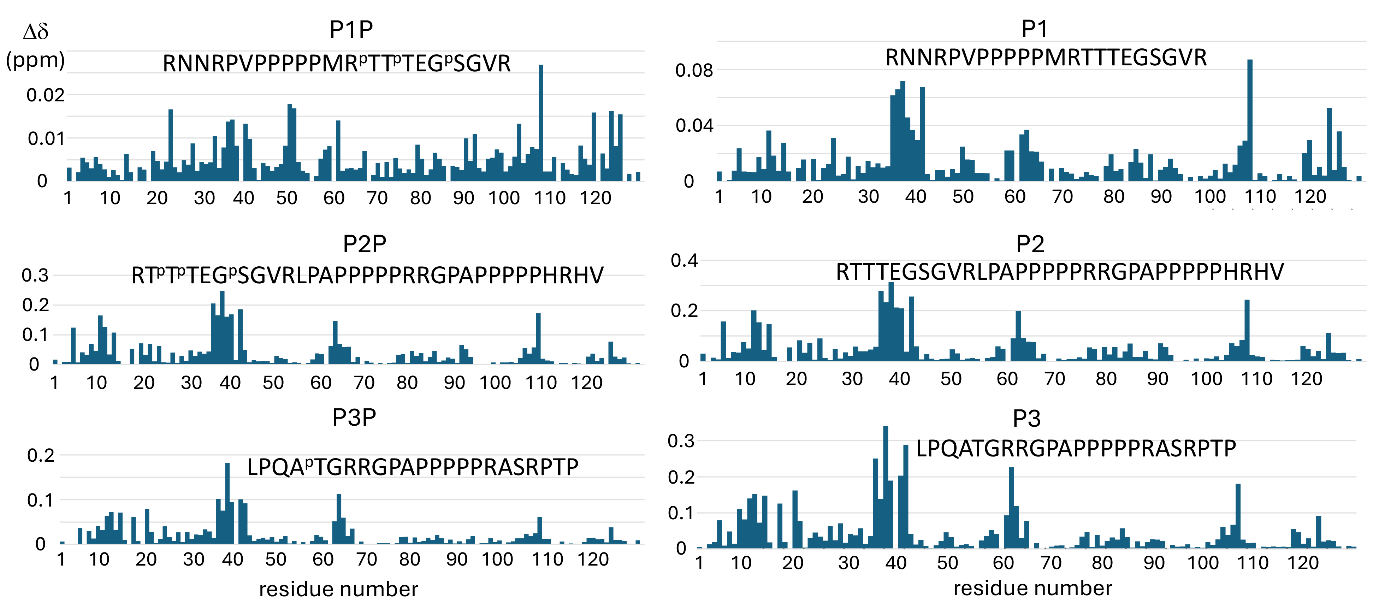


**Figure S9.** **Binding of phosphorylated Las17 peptides to Sla1 SH3 domains 1 and 2.** Phosphorylation sites are indicated by superscript p. The plots show measured NMR chemical shift changes in ^15^N-labelled SH3#12 on addition of phosphorylated peptides corresponding to binding sites PP1, PP3 and PP4, to make 1:1 complexes. The shift changes are much smaller than those with unphosphorylated peptides (shown on the right for comparison), indicating that phosphorylated PP1 binds about 4-fold less; PP3 binds roughly half as strong; and PP2 binds about 20% weaker. They all bind in the same way as their unphosphorylated forms, because the pattern of shift changes is similar. The original data is deposited at BMRB as BMRbig129.

A detailed description of the model

## 1. Outline of the model

In the model, proteins can be envisioned as “beads on a string”. All peptide constructs are described by two agent types: platforms and domains. PlatformAgents hold the position of up to five vectors - the number of vectors updated is dependent upon the peptide it represents. This data class can therefore be envisioned as a string in the “beads on a string” analogy as they hold the object’s spatial position and execute its movement. Platform agents are critical in maintaining the spatial coherence of multi-domain peptides such that the domains do not move apart by a distance greater than their peptide linker. The term “complex” refers to two or more platforms which are bound together. Complex IDs are generated dynamically each time a complex is either formed or changes in composition. No limit is set on how many platforms can exist within a single complex as, theoretically, agents may ‘daisy chain’ together to form larger complexes and this potential spatial organisation should not be disallowed.

DomainAgents hold the interaction-specific variables and govern the binding and unbinding function phases. In the “beads on a string” analogy, domains represent the beads. Each domain is uniquely associated with one of the five vector positions of a PlatformAgent. These domains are referred to as the “child domains” belonging to the “parent” PlatformAgent. This data class updates its position once per timestep by looking up its parent platform from a list of messages. Every functional peptide motif is represented as a domain agent including actin monomers, individual SH3 domains and each of the five binding motifs of Las17.

The third agent type are the FilamentAgents, which keep track of F-actin filaments. Each agent has a pointed and barbed end number, filament size, and end positional vector. Actin domains can bind, and be incorporated into, FilamentAgents.

### 2. Actin nucleation

The model can be set to permit nucleation either by cross-filament nucleation or by tandem linear nucleation.

In cross-filament nucleation, if three actin monomers are bound to Las17 at the same time, the actin monomers automatically form an F-actin seed which can start to polymerise; alternatively, F-actin filaments can dissociate. Each of these binding and dissociation steps has a certain probability (calculated from their rates) that is accessed as a global variable array *domain1_bind_prob*, *domain2_bind_prob*, *domain1_unbind_prob*, *domain2_unbind_prob*.

In tandem linear nucleation, actin nuclei (formed by Las17 after it has bound 3 actins) can only transition into a seed once a fourth and fifth actin is bound. Here, it is assumed that a fourth actin monomer would associate with two of the nuclei subunits via lateral contacts to form a double stranded base to the growing filament. Rates for this reversible interaction were taken from the literature (Sept and McCammon 2001): *k*_on_ = 2.18 µM^-1^ s^-1^, *k*_off_ = 1.30×10^3^ s^-1^) and are consistent with the dominant hypothesis for Spire and Cobl (Sitar et al. 2011). These values are assigned to the *SIDE_PON* and *SIDE_POFF* global variables. These values can be replaced in the initiation file by standard filament polymerisation rates to change Las17 to a cross-filament tandem nucleator, thereby allowing easy changing between the two nucleation mechanisms. Filament agents are not classed as a seed until a fifth monomer binds to “lock in” the fourth subunit. Following this interaction, the oligomer is assumed to occupy the typical structure of a dual stranded, pentameric filament and thus transition to an actin seed is complete. Further gains and losses of actin monomers are processed as typical filament interactions.

### 3. Agent homodimerisation

All platforms have the possibility to form a homodimer. Dimerisation is mediated by a sixth domain agent – termed “dimerisation domain” – which is unique to the other five possible agents which constitute protein functional motifs. The position of this agent is calculated separately to the other domains and is not retained by the parent platform as it simply occupies the platforms’ central vector. Dimerisation domains can bind other dimerisation domains of the same protein type using the global array variables: *DIMER_BIND_RADII*, *DIMER_BIND_PROB*, and *DIMER_UNBIND_PROB*. The binding and unbinding variables can be changed if certain conditions are met (e.g., one of the parent platforms is actin-bound) to *COOP_DIMER_BIND_PROB* and *COOP_DIMER_UNBIND_PROB*. Dimerisation allows all child domains to access alternative binding/binding probabilities.

## 4. Dynamic hierarchy

An overarching design principle allows all agent types to move, interact, and change states in parallel whilst remaining in communication with one another. This linking principle is hierarchy and it had to be embedded in every part of the model’s functionality. A rigidly enforced hierarchy was important to allow agents to freely form multi-peptide complexes of any size. For example, if a binding complex contains three agents, then these agents must move sequentially, rather than in parallel, to maintain spatial coherence of the structure (**Figure S10**). Priority values are used to determine which code iteration the agent is permitted to move within (termed “priority blocks”). This is important when the program is run in a parallel environment. Binding information between two domains is stored in each of domain agents as their position within the priority hierarchy may change as other binding/unbinding events occur within their complex.

| 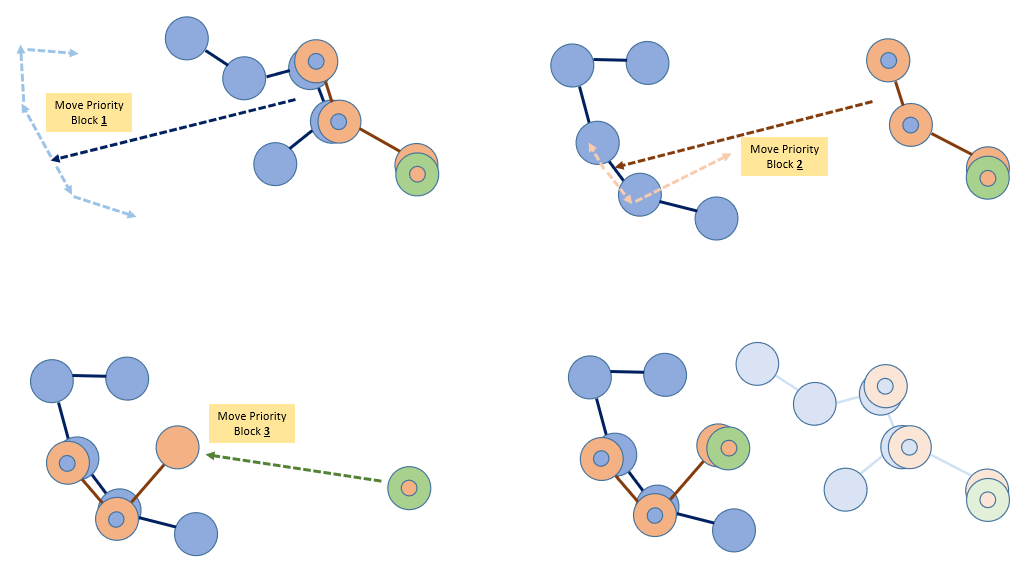  **II**  **I**  **III**  **IV** |
| --- |
| **Figure S10. Simplified model flowchart**. Agent complexes move iteratively via a priority system. This prevents bound agent domains (shown with a small inner circle matching the colour of their bound agent) moving in a manner independent of the rest of the complex. (I) Agents of priority block 1 first move via their central position (dark dashed arrow) and then re-calculate the position of any peptide domains using linker lengths with random orientations (lighter dashed arrows). (II-III) This then repeats for each ascending priority block with the initial movement (dark dashed arrow) being set by any domains bound to higher priority agents. (IV). This continues until all agents within the complex are moved to a new location (old location shown by faded image). |

These movement hierarchies must also be dynamic to reflect the transient nature of such complexes. This is because both binding and unbinding interactions can change which agents are associated with a complex, and where in the hierarchy that agent is located (**Figure S11**).

Of consideration is that some agents (e.g., Las17 and Sla1) contain multiple domains (referred to as a tandem arrangement) and each can form their own interactions. Also, breaking of one domain-domain interaction within a tandem may not cause the agent to dissociate from a complex as another domain from the same agent may still be bound. Consequently, priority values are re-determined following all binding and unbinding events with the agent that initiated the event being set as priority 1. Priority values increase with distance from the priority 1 agent such that a continuous chain of values is maintained (illustrated by the priority values changes shown in **Figure S11**).

| 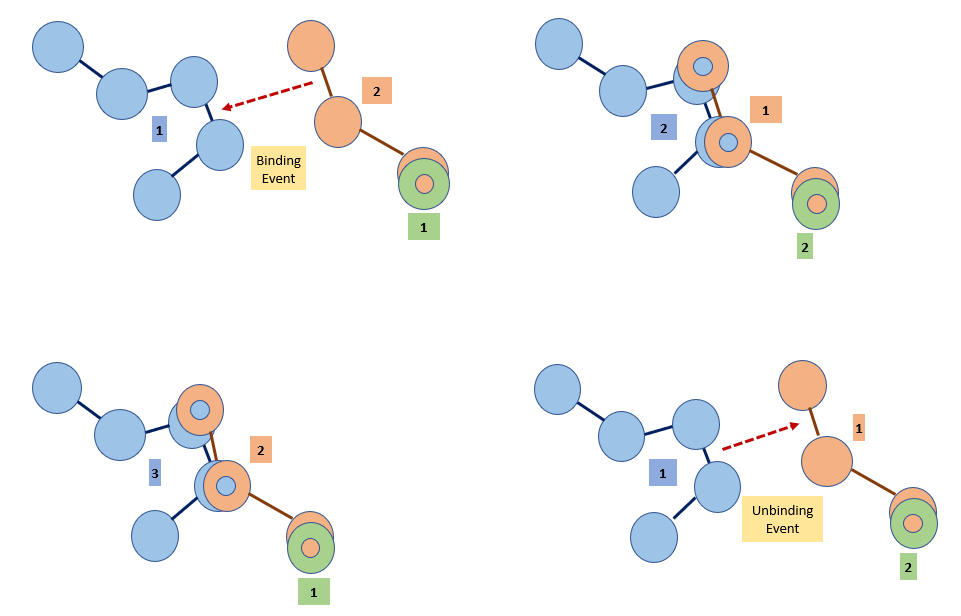  **IV**  **II**  **I**  **III** |
| --- |
| **Figure S11. Simplified model flowchart**. Agents binding and unbinding from one another change the makeup of complexes, which requires a recalculation of priority values. Three agents are shown within these examples: agent X in blue, agent Y in orange, and agent Z in green. Priority values are given in boxes the colour of their respective agents. Binding is shown using a small inner circle matching the colour of the bound partner. (I-II) An example of a binding interaction between agents X and Y (I) necessitating a change in the priority values (II). The method for determining these values was designed to reduce the number of priority blocks (hence the central agent became priority 1). (III-IV) An example of an unbinding interaction between agents X and Y (III) necessitating a change in the priority values (IV). |

### 5. A distinction between simulation iteration and timestep

In FLAME GPU 1, agents can possess multiple “agent states” that act as guard loops for functions. Each function must define a currentState (the state agents must occupy to use the function) and a nextState (the state the agent moves into following the function). All three agent types can exist in one of two states – *resolved* and *unresolved*. Unresolved states can only access the movement phase while resolved states execute the remaining phases, which uncouples the iteration and timestep. Functions were written to ensure that all agents switch states simultaneously with a change to the *resolved* state occurring once all agents have successfully moved and a return to the *unresolved* state occurring after the timestep has concluded.

We enforce hierarchy when moving agents. To overcome the drawback of parallel execution within functions, and to allow sequential agent movement, priority blocks were used. At the start of the timestep, the first priority block is moved within the *top priority movement phase* followed by the second priority block within the *lower priority movement phase*. If there are any agents with a priority value greater than two, then the current iteration ends and a new iteration begins. The term iteration used throughout follows that of FLAME GPU with each run through the function list by the program defining a function (even if many of those functions are not executed).

The next sequential priority block is then moved using the *lower priority movement phase* code. This continues until all agents have successfully moved position. Upon the achievement of this criterion, agents shift from an *unresolved* state to a *resolved* state wherein the rest of the code can be executed. This includes the binding and unbinding functionality. Upon completion of this code, the agents return to an *unresolved* state ready to begin a new timestep. The term timestep used throughout defines each period when agents occupy the *resolved* state as this is when agent interactions occur, and data is recorded. The following figure (**Figure S12**) visually demonstrates this by showing the major code phases and path of programmatic execution.

| 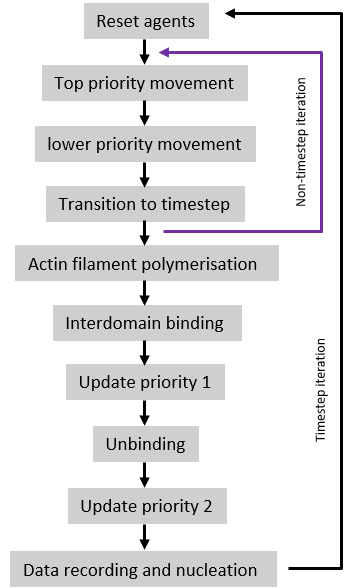 |
| --- |
| **Figure S12. Simplified model flowchart**. The functions can be grouped into ten phases: reset agents, top priority movement, lower priority movement, transition to timestep, polymerisation, interdomain binding, first priority update, unbinding, second priority update, and actin nucleation. These phases are shown in grey boxes whilst the flow of the code is given by the black arrows. An iteration is defined as each time the code repeats a function. The purple arrow shows the only code that can be accessed within a non-timestep loop (*unresolved* state). All functions are accessed during a timestep iteration as agents transition from an *unresolved* state to a *resolved* state before transitioning back to an *unresolved* state at the beginning of the next iteration. |

On average, the number of iterations exceeds the number of timesteps achieved by between two and three times due to this uncoupling of timestep and simulation iteration. When requesting n timesteps, the program must therefore be executed for >n*2 iterations to account for this. The exact number of iterations required depends upon the size that complexes reach during the simulation and so cannot be predicted exactly. Iterations are only repeated as a consequence of how priority blocks are moved and not other conflict. For example, binding conflicts are resolved by prioritising the interaction with the closest distance.

## 6. Flowchart

**Figure S13** is a detailed flowchart of the simulation including functions and message pathways. Agents communicate with each other by writing messages during the execution of a function.


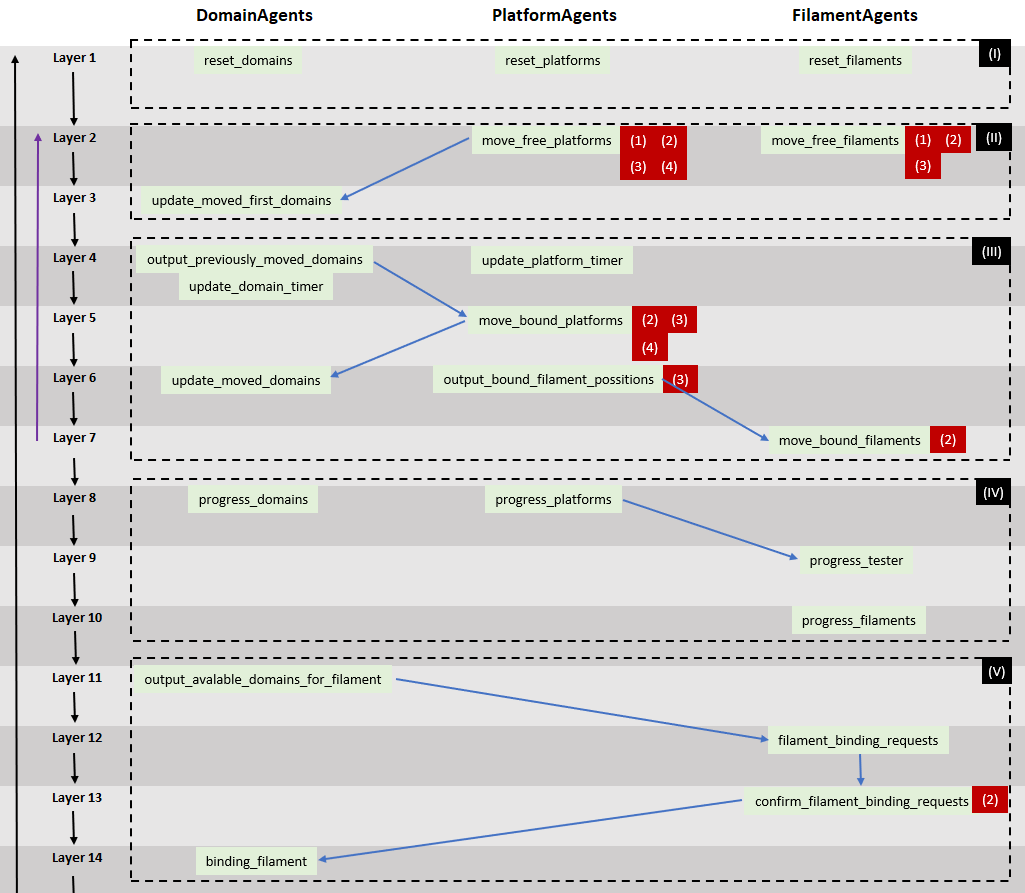
No more than one message can be written per agent. Other agents then access this message list during the execution of a function, although only one message list can be accessed per function. This requires careful planning of messages. For example, some messages may contain variables not intended for the agents that receive them but are instead passed along to later functions.

| **Figure S13. Detailed model flowchart (continued)**  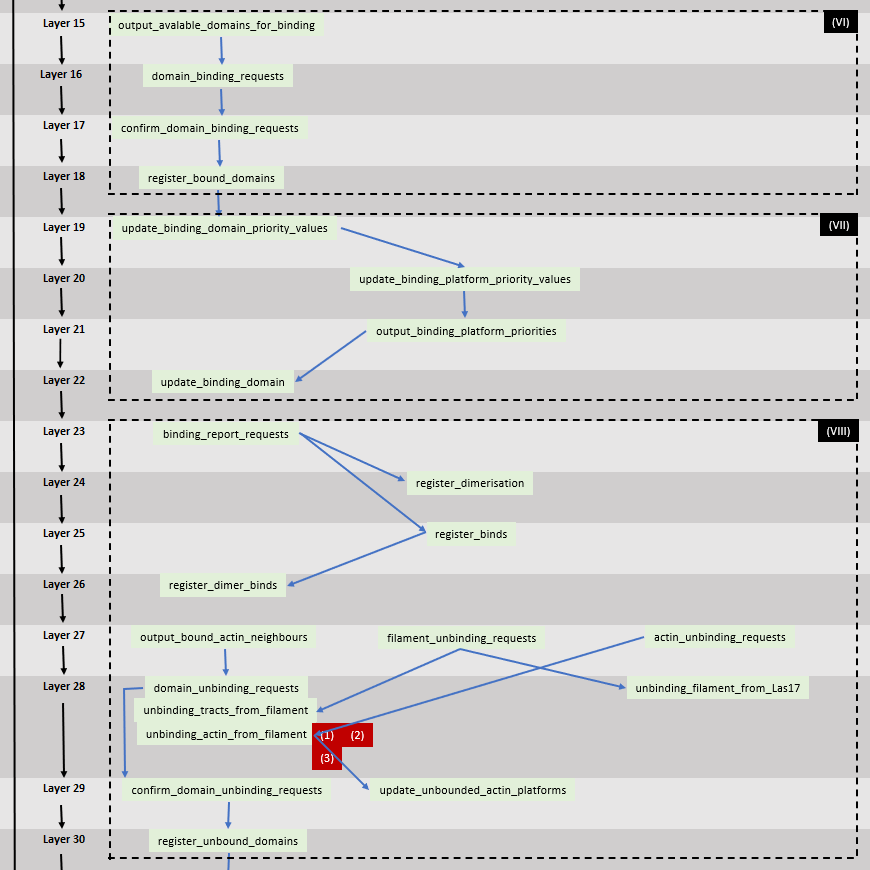 |
| --- |
| 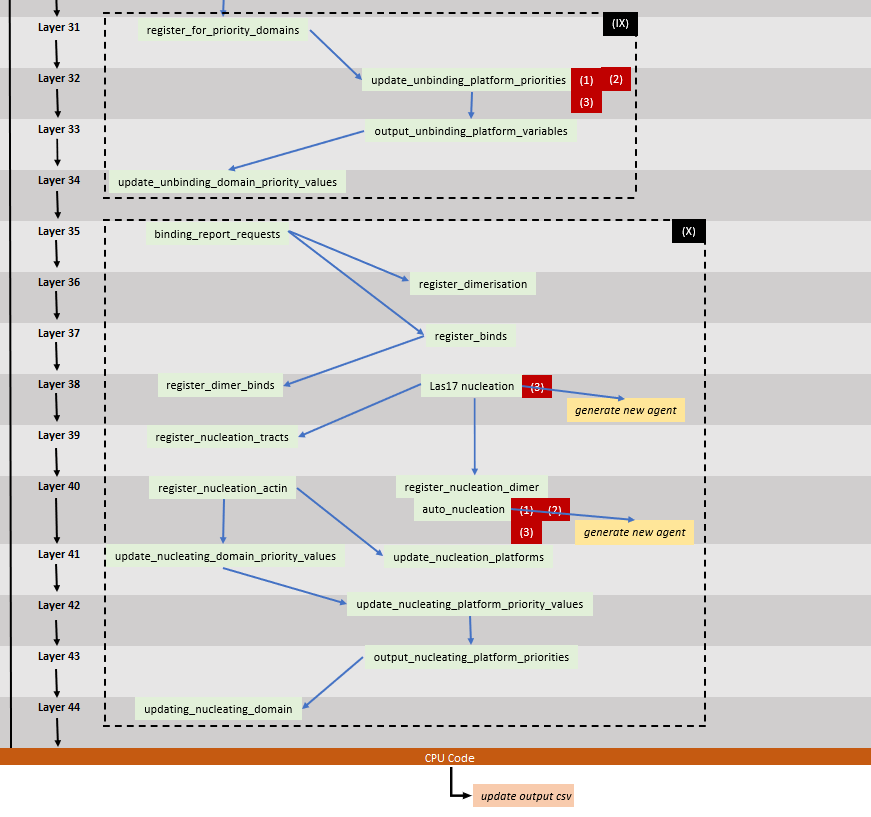 |
| **Figure S13. Detailed model flowchart**. The flow of code during runtime is shown by the black line with a new iteration beginning each time Layer 1 is executed. An iteration can also be considered a timestep if agents are able to transition into a resolved state during phase IV (*Transition to timestep*) and access function layers 11-44. Only layers covered by the purple arrow can be accessed during a non-timestep iteration. All layers (including the CPU code) are technically read during non-timestep iterations, however, functions not covered by the purple arrow cannot be executed due to being in the wrong state. Green boxes in the appropriate agent class column (as shown along the top) show the functions and all functions within a layer are executed simultaneously by all applicable agents. Blue arrows represent message lists with the function at the base of the arrow generating the list and the function(s) at the arrowhead reading the messages. Each function can only produce and accept a single message list. Red boxes show when global functions are accessed (1 = boundryCheck, 2 = movement_calc, 3 = random_angle, and 4 = random_distance). Functions can be broadly grouped into ten phases as first given in **Figure S12**. These include (I) reset agents, (II) top priority movement, (III) lower priority movement, (IV) transition to timestep, (V) polymerisation, (VI) interdomain binding, (VII) first priority update, (VIII) unbinding, (IX) second priority update, and (X) actin nucleation. |

## 7. XML initiation file

An XML file defines the structures of simulation elements along with what components run, in what order and with what conditions. We use three data classes to define the agents: PlatformAgent, DomainAgent, FilamentAgent. Each data class retains a set of agent variables which are updated during the simulation runtime. These agent types are defined throughout the next sections.

### 7.1. Global Variables

There are 31 global variables defined within the XML file. Global variables can only be changed during the CPU code at the end of each time step. They can be accessed – though not edited – by all agent functions where they are called. These variables are used to hold information that is specific to the simulation but not specific agents. This reduces the memory requirement during model construction as shared agent variables can be allocated as a global instead. Below is an overview of all global variables defined in the model (**Table S2**).

| \| **Variable Name** \| **Description** \| **Functions referenced** \| \| --- \| --- \| --- \| \| TIME_COUNTER \| Used to hold the current iteration number of the simulation timestep. \| CPU code \| \| PRINT_COUNTER \| A counter variable that is increased by 1 every iteration and reset to 0 after an output is made to the csv file. This allows lines to be saved every **X** iterations thus saving memory. \| CPU code \| \| ITERATION \| The current iteration following the last timestep conclusion. For example, in the model iteration directly following a timestep conclusion, this value will be 1. \| CPU code \| \| ITERATION_PLUS_ONE \| This holds the value of “ITERATION + 1” so that the calculation does not have to be undertaken every time a function uses it. ITERATION_PLUS_ONE is referenced by agents (along with their priority value) which have not moved during the *lower priority movement phase* in this timestep to determine when the agent can move. ITERATION_PLUS_ONE is saved by agents into agent variables for lookup by function guard loops. \| update_platform_timer  update_domain_timer \| \| XMAX  YMAX  ZMAX \| These define the maximum allowed axis value for their respective axis before a boundary condition will act upon the agent. \| boundryCheck \| \| XMIN  YMIN  ZMIN \| These define the minimum allowed axis value for their respective axis before a boundary condition will act upon the agent. \| boundryCheck \| \| BIND_RADII \| A 196-unit array holding the binding radii for each possible pairwise interaction. \| domain_binding_requests  confirm_domain_  unbinding_requests \| \| BIND_PROB \| A 196-unit array holding the binding probability for each possible pairwise interaction \| domain_binding_requests \| \| UNBIND_PROB \| A 196-unit array holding the unbinding probability for each possible pairwise interaction \| domain_unbinding_requests \| \| DIMER_BIND_RADII \| A 6-unit array holding the binding radii for each possible homodimerisation. \| domain_binding_requests \| \| BIND_PROB_DIMERISED \| A 196-unit array holding the binding probability for each possible pairwise interaction when the domain requesting the bind belongs to a homodimerized platform. \| domain_binding_requests \| \| UNBIND_PROB_DIMERISED \| A 196-unit array holding the unbinding probability for each possible pairwise interaction when the domain requesting the unbind belongs to a homodimerized platform. \| domain_unbinding_requests \| \| DIMER_BIND_PROB \| A 6-unit array holding the probability of homodimerisation for each agent type should two of the agents be separated by a distance less than the binding radii. \| domain_binding_requests \| \| COOP_DIMER_BIND_PROB \| A 6-unit array holding the probability of homodimerisation when a cooperativity criterion is met. In the case of Las17, this is when the agent is bound by actin. \| domain_binding_requests \| \| DIMER_UNBIND_PROB \| A 6-unit array holding the probability of each possible homodimer dissociating \| domain_unbinding_requests \| \| COOP_DIMER_UNBIND_PROB \| A 6-unit array holding the probability of each possible homodimer dissociating when a cooperativity criterion is met. In the case of Las17, this is when the agent is bound by actin. \| domain_unbinding_requests \| \| PP1_PP3_COOPERATIVITY \| The unbinding probability between actin and the first or second actin-binding tracts are divided by this value if both the first and second acting-binding tracts are occupied by actin. \| domain_unbinding_requests \| \| PP3_PP4_COOPERATIVITY \| The unbinding probability between actin and the second or third actin-binding tracts are divided by this value if both the second and third acting-binding tracts are occupied by actin. \| domain_unbinding_requests \| \| ACTIN_BARB_INTERACTION_RADIUS \| The radius of interaction between actin agents and the barbed end of filament agents \| filament_binding_requests  unbinding_actin_  from_filament \| \| ACTIN_POINT_INTERACTION_RADIUS \| The radius of interaction between actin agents and the pointed end of filament agents \| filament_binding_requests  unbinding_actin_  from_filament \| \| SEED_UNBIND_PROB \| The probability of actin filaments unbinding the Las17 agent which nucleated it. \| filament_unbinding_requests \| \| ACTIN_DIAMETER \| Filament length is governed by the number of actin subunits multiplied by ACTIN_DIAMETER. \| move_free_filaments  move_bound_filaments  auto_nucleation \| \| FILAMENT_BASE_K \| A scaled variable used to define the movement distance for a filament during a movement function. \| confirm_filament_  binding_requests  actin_unbinding_requests  register_binds_and_nucleate  auto_nucleation \| \| FILAMENT_ROTATE_BASE_K \| A scaled variable used to define the rotational speed for a filament during a movement function. \| confirm_filament_  binding_requests  actin_unbinding_requests  register_binds_and_nucleate  auto_nucleation \| \| BARB_POFF \| The probability of an actin agent dissociating from the barbed end of a filament during each timestep. \| actin_unbinding_requests \| \| POINT_POFF \| The probability of an actin agent dissociating from the pointed end of a filament during each timestep. \| actin_unbinding_requests \| \| SIDE_POFF \| The probability of an actin agent dissociating from an actin nucleus four subunits in size during each timestep. \| actin_unbinding_requests \| \| BARB_PON \| The probability of an actin agent binding to the barbed end of an actin filament. \| filament_binding_requests \| \| POINT_PON \| The probability of an actin agent binding to the pointed end of an actin filament. \| filament_binding_requests \| \| SIDE_PON \| The probability of an actin agent binding to an actin nucleus three subunits in size. \| filament_binding_requests \| \| INITIAL_ACTIN_CONC \| The initial concentration of actin is used to determine the rate of salt-mediated actin nucleation during each timestep. \| auto_nucleation \| \| FREE_ACTIN_CONC \| The current concentration of actin is used to determine the rate of salt-mediated actin nucleation during each timestep. \| auto_nucleation \| \| NUCLEATION_RATE \| The nucleation rate is used to determine the rate of salt-mediated actin nucleation during each timestep via the following formula.  NUCLEATION_RATE*(FREE_ACTIN_CONC  /INITIAL_ACTIN_CONC) \| auto_nucleation \| \| FILAMENT_PITCH \| This value is multiplied by the number of subunits contained within a filament to determine the length of that filament. The value is derived from the per subunit rise within actin filaments \| move_free_filaments  move_bound_filaments  confirm_filament_  binding_requests \| \| D1MAX  D2MAX  D3MAX  D4MAX \| A set of four, 10-unit arrays describing the minimum separations of each peptide. D1 holds the domain1-domain2 distance, D2 holds the domain2-domain3 distance, etc. \| move_free_platforms  move_bound_platforms \| \| D2_CENVEC2_MAX \| A 10-unit array describing the maximum separation between domain 2 and the centre of each peptide sequence. \| move_free_platforms \| \| D2_CENVEC3_MAX \| A 10-unit array describing the minimum separation between domain 3 and the centre of each peptide sequence. \| move_free_platforms \| \| D1MIN  D2MIN  D3MIN  D4MIN \| A set of four, 10-unit arrays describing the minimum separations of each peptide. D1 holds the domain1-domain2 distance, D2 holds the domain2-domain3 distance, etc. \| move_free_platforms  move_bound_platforms \| \| COMPLEX_BASE_K \| When multiple proteins are bound together, this value (divided by the number of peptides in the complex) is used for the scaling movement variable. \| move_free_platforms \| \| MAX_BOUNDED_ATTEMPTS \| This is the maximum number of attempts a PlatformAgent will make when moving a domain flanked by bound domains. If this value is exceeded, then a position equidistant from the flanking domains is chosen. This variable prevents excessive loop iterations. \| execute_bounded_movement  move_bound_platforms \|   **Table S2. Global variables**. A list of all the global variables used, their descriptions, and which functions they refer to (including global functions, agent functions, and the CPU function). |
| --- | --- | --- | --- | --- | --- | --- | --- | --- | --- | --- | --- | --- | --- | --- | --- | --- | --- | --- | --- | --- | --- | --- | --- | --- | --- | --- | --- | --- | --- | --- | --- | --- | --- | --- | --- | --- | --- | --- | --- | --- | --- | --- | --- | --- | --- | --- | --- | --- | --- | --- | --- | --- | --- | --- | --- | --- | --- | --- | --- | --- | --- | --- | --- | --- | --- | --- | --- | --- | --- | --- | --- | --- | --- | --- | --- | --- | --- | --- | --- | --- | --- | --- | --- | --- | --- | --- | --- | --- | --- | --- | --- | --- | --- | --- | --- | --- | --- | --- | --- | --- | --- | --- | --- | --- | --- | --- | --- | --- | --- | --- | --- | --- | --- | --- | --- | --- | --- | --- | --- | --- | --- | --- | --- |

### 7.2. Data Class: DomainAgent

Each functional motif of a peptide is represented by a domain agent (e.g., each of the binding motifs within the 300-422 peptide, actin monomers, and SH3 domains). DomainAgents are linked with one of the five vectors of a specific PlatformAgent with the latter governing movement and DomainAgents governing interactions. **Table S3** shows the agent variables held in the DomainAgent data class.

|  |
| --- |

| **Variable Name** | **Description** | **Default value** |
| --- | --- | --- |
| Id | A unique identification number used to reference an individual agent. | Unique integer |
| type | The “type” variable identifies the motif/domain that this agent represents. This allows the agent to access the correct binding rates. Up to 14 types can be set by the user with all possible pairwise interactions between types being set in the global arrays. | 0-13 |
| domain_index | An index to identify which of the five PlatformAgent vectors this agent is linked to (0=first domain, 1=second domain etc.) | 0-4 |
| platform_id | The unique identification number of the PlatformAgent this domain is linked to. This allows for the updating of vector variables. | Unique integer of a PlatformAgent |
| platform_priority | The priority value of the peptide this domain belongs to. Lower values move before higher values to maintain spatial coherence. Peptides in a complex are dynamically given a priority value. | 1 if unbound and (possibly) higher if in a complex |
| platform_complex_size | The number of peptides contained within the complex this agent is associated with. | Equal to the number of peptides bound (directly or indirectly) to this peptide |
| platform_complex_id | The unique identification number of the complex this domain is a part of. The complex id is dynamically generated upon the formation of a new complex and allows agents that are part of the same complex to communicate with each other. | Unique integer of the binding complex this agent is associated with |
| platform_species | The “species” variable identifies the peptide that this agent is associated with. It consists of Las17 (0), actin (1), cloud SH3s (2), Ysc84 (3), Sla1 (4), and Bzz1 (5). | 0-5 |
| vector | This 3-unit vector object holds the position of the domain within the 3D simulation space. The value is obtained each timestep from the agent’s linked PlatformAgent. | random |
| awaiting_movement_domain | A Boolean variable used to distinguish agents that have moved this timestep (1) and those that have not (0). This variable is used to maintain synchronicity between agents so that some do not enter the timestep functions before others. | 0 |
| awaiting_confirmation | A Boolean variable used to signify if an agent attempted to bind another agent during the current timestep. This is used as a function guard so that subsequent functions in the binding phase are only accessed by the appropriate agents. | 0 |
| binding_state | A variable to show the current bound state of the agent with 0 being unbound, 1 being bound, 2 representing actin incorporated into a filament (F-actin), and 3 being a Las17 tract bound to a FilamentAgent. | 0 if unbound and (possibly) higher if in a complex |
| dimerised | A variable to show the current homodimerisation state of the parent platform. This variable holds a value of -1 when not dimerised and the id number of the dimerised partner when in a dimer. | -1 if monomeric and higher if dimerised |
| bound_id | The *id* of the DomainAgent bound if there is an ongoing interaction. | -1 if unbound and higher if in a bound state |
| bound_type | The *type* of the DomainAgent bound if there is an ongoing interaction. | -1 if unbound and higher if in a bound state |
| bound_platform | The *platform_id* of the DomainAgent bound if there is an ongoing interaction. | -1 if unbound and higher if in a bound state |
| bound_filament_id | The *id* of the FilamentAgent bound if there is an ongoing interaction. | -1 if unbound and higher if in a bound state |
| bound_domain_index | The *domain_index* of the DomainAgent bound if there is an ongoing interaction. | -1 if unbound and higher if in a bound state |
| requested_id | A temporary variable to hold the *id* of the DomainAgent attempting to bind this agent prior to binding confirmation. | -1 |
| requested_type | A temporary variable to hold the *type* of the DomainAgent attempting to bind this agent prior to binding confirmation. | -1 |
| requested_platform | A temporary variable to hold the *platform_id* of the DomainAgent attempting to bind this agent prior to binding confirmation. | -1 |
| requested_complex | A temporary variable to hold the *platform_complex_id* of the DomainAgent attempting to bind this agent prior to binding confirmation. | -1 |
| requested_domain_index | A temporary variable to hold the *domain_index* of the DomainAgent attempting to bind this agent prior to binding confirmation. | -1 |
| binding_dist | A temporary variable used to hold the distance between this agent and the DomainAgent attempting to bind it, prior to binding confirmation. This is used to prioritise between competing interactions for the same agent. | -1 |
| filament_id | If the agent represents actin and is incorporated into a filament, this variable holds the unique identification number of the FilamentAgent which it is a part of. | -1 if free G-actin and higher if in an F-actin state |
| filament_subunit_number | This variable is only accessed by actin agents. If it is incorporated into a filament, this variable holds its position within the filament. | 0 if free G-actin and different if in an F-actin state |
| probability_request | A random uniform number between 0 and 1 used to identify which agent in a prospective interaction will act as the dominant partner (i.e., having one agent “take the lead” to prevent A binding B if B is also trying to bind A). | -1 |
| update_priority_domain | A reporter Boolean used to indicate when a complex needs to update its priority values. | 0 |
| domain_turn_counter | An agent variable used to hold the current value of the global ITERATION_PLUS_ONE variable for use in a guard loop. | 2 |
| bound_report_domain | During the *report and reset phase*, this agent holds the binding information of the DomainAgent using the equation “xagent->type*100) + (xagent->bound_type+1”. For example, a value of 006 would indicate that this agent is a Las17 bm1 agent (+0*100) currently bound to an actin DomainAgent (+6). This variable is used by the CPU code to analyse global populations of specific interactions. | 0 |
| unbind_dimer_block | A Boolean that when equal to 1, prevents the homodimer from dissociating. This prevents dissociation when bound to an actin filament which is both kinetically extremely unlikely and presents a series of programmatic obstacles. | 0 |
| actin_within_dimer | A Boolean accessed by domains with homodimerized parent platforms. The value equals 1 when an actin agent is bound to the parent platforms’ dimerised partner. | 0 if no actin within its dimerised partner, otherwise 1 |
| cooprative_dimerisation_PP1 | A Las17 variable to indicate if bm2 of parent platform is actin-bound (10), bound to a non-actin domain (1), or completely unbound (0). | 0 if unbound, otherwise either 1 or 10 |
| cooprative_dimerisation_PP3 | A Las17 variable to indicate if bm4 of parent platform is actin-bound (10), bound to a non-actin domain (1), or completely unbound (0). | 0 if unbound, otherwise either 1 or 10 |
| cooprative_dimerisation_PP4 | A Las17 variable to indicate if bm5 of parent platform is actin-bound (10), bound to a non-actin domain (1), or completely unbound (0). | 0 if unbound, otherwise either 1 or 10 |
| dimer_adjacent_bound_type | A variable accessed by domains with homodimerized parent platforms. Its value is the *type* variable of the domain bound to the equivalent binding motif of the parent platforms’ dimerised partner. | 0 if not dimerised, otherwise variable |

| **Table S3. The data class variables of DomainAgents**. A list of all the agent-specific variables found within the DomainAgent data class, their descriptions, and default values given during creation of the initiation XML file. |
| --- |

### 7.3. Data Class: PlatformAgent

Every peptide is represented by a PlatformAgent, which can be associated with up to five DomainAgents (one linked with each vector). PlatformAgents govern the movement of peptides. **Table S4** shows the agent variables held in the PlatformAgent data class.

| \| **Variable Name** \| **Description** \| **Default value** \| \| --- \| --- \| --- \| \| id \| A unique identification number used to reference an individual agent. \| Unique integer \| \| species \| The “species” variable identifies the peptide that this agent represents. It comprises Las17 (0), actin (1), cloud SH3s (2), Ysc84 (3), Sla1 (4), and Bzz1 (5). \| 0-5 \| \| complex_size \| The number of proteins (PlatformAgents) within the same complex as this agent \| 1 if not in a complex \| \| dimerised_partner_id \| The ID variable of the platform homodimerised to this agent. \| 0 if non dimerised, otherwise higher \| \| complex_id \| The unique identification number of the complex this domain is a part of. The complex id is dynamically generated upon the formation of a new complex and allows agents that are part of the same complex to communicate with each other. \| Unique integer \| \| k \| This variable is scaled based on the diffusion coefficient of the peptide it represents and governs the rate of diffusion. \| Value depends upon the peptide size \| \| vector1  vector2  vector3  vector4  vector5 \| Each of these five variables are fvec3 vector variables holding three coordinate values (x, y, and z). They retain the positions of the possible domains that can be associated with the platform. \| Unique integer of the binding complex this agent is associated with \| \| priority \| The priority of this peptide. Lower values move before higher values to maintain spatial coherence. Peptides in a complex are dynamically given a priority value. \| 1 if unbound and (possibly) higher if in a complex \| \| update_priority \| A Boolean variable to indicate if a platform needs to update its priority due to a change in the complex it is associated with (e.g., a binding or unbinding event). \| 0 \| \| awaiting_movement_platform \| A Boolean variable used to distinguish agents that have moved this timestep (1) and those that have not (0). This variable is used to maintain synchronicity between agents so that some do not enter the timestep functions before others. \| 0 \| \| vec1_binding_partner  vec2_binding_partner  vec3_binding_partner  vec4_binding_partner  vec5_binding_partner \| These variables hold the *type* values of any associated domains. Values are used in the *report and reset phase* for both reporting to the output file and nucleating bound actin monomers into actin filaments (if three actin monomers are bound to three adjacent domains simultaneously). \| -1 \| \| vec1_dimer_partner  vec2_dimer_partner  vec3_dimer_partner  vec4_dimer_partner  vec5_dimer_partner \| These variables hold the *type* values of any domains bound to the indicated position on its homodimerised partner. \| -1 \| \| seed_id \| The unique identification number of any filament bound to this platform (only used if this platform is Las17). \| -1 if unbound to an actin filament and bound if higher \| \| factin \| A Boolean variable used by actin platforms to indicate if they have been incorporated into a filament (1) or are free (0). \| 0 if monomeric and 1 if located in a filament \| \| filament_bound \| The ID value of the actin filament bound to this platform if one is bound. \| -1 if not bound to a filament, higher if so \| \| NUCLEATION_EVENTS \| A counter that increases by one every time this platform nucleates a new actin filament. This is used for data recording purposes. \| 0 \| \| NON_LAS17_NUCLEATION_EVENTS \| A counter that increases by one every time an actin filament is nucleated by salt. One Las17 platform can nucleate three random actins if a random number is greater than the nucleation probability of a timestep. \| 0 \| \| platform_turn_counter \| An agent variable used to hold the current value of the global ITERATION_PLUS_ONE variable for use in a guard loop. \| 2 \| \| bound_report_platform \| During the *report and reset phase*, this agent holds the complex information of platforms. Values are calculated using the formula “(xagent->species*100) + (xagent->complex_size”). For example, a value of 401 would be given for a Sla1 protein (+4*100) that is unbound (+1) and thus not in a multi-platform complex. \| 0 \|   **Table S4. The data class variables of PlatformAgents**. A list of all the agent-specific variables found within the PlatformAgent data class, their descriptions, and default values given during creation of the initiation XML file. |
| --- | --- | --- | --- | --- | --- | --- | --- | --- | --- | --- | --- | --- | --- | --- | --- | --- | --- | --- | --- | --- | --- | --- | --- | --- | --- | --- | --- | --- | --- | --- | --- | --- | --- | --- | --- | --- | --- | --- | --- | --- | --- | --- | --- | --- | --- | --- | --- | --- | --- | --- | --- | --- | --- | --- | --- | --- | --- | --- | --- | --- |

### 7.4. Data Class: FilamentAgent

A FilamentAgent is dynamically built during the simulation runtime to represent an actin filament. Upon complete dissolution of the filament, the respective FilamentAgent is “killed” during the timestep when this occurred. **Table S5** shows the agent variables held in the FilamentAgent data class.

| \| **Variable Name** \| **Description** \| **Default value** \| \| --- \| --- \| --- \| \| Id \| A unique identification number used to reference an individual agent. \| Unique integer \| \| Size \| The number of actin subunits in the filament. \| 3 \| \| K \| This variable is scaled based on the diffusion coefficient of the domain it represents and governs the rate of diffusion. \| 1.2 \| \| rotation_k \| This variable scales the rate of rotation for a ScaffoldAgent. \| 2 \| \| axis_phi \| Axis_phi is a polar coordinate for the barb to point axis. \| random \| \| axis_theta \| Axis_theta is a polar coordinate for the barb to point axis. \| random \| \| filament_centre \| An fvec3 vector variable holding three coordinate values (x, y, and z). *filament_centre* marks the centre of the filament is the point of movement and rotation for the overall filament. \| random \| \| Barb \| An fvec3 vector variable holding three coordinate values (x, y, and z). *barb* represents the position of the barbed end. \| random \| \| Point \| An fvec3 vector variable holding three coordinate values (x, y, and z). *point* represents the position of the pointed end. \| random \| \| awaiting_movement_filament \| A Boolean variable used to distinguish agents that have moved this timestep (1) and those that have not (0). This variable is used to maintain synchronicity between agents so that some do not enter the timestep functions before others. \| 0 \| \| execute_procession \| A reporter Boolean that becomes true once all the DomainAgents and PlatformAgents are ready to enter the timestep. This variable is then used to activate *awaiting_movement_filament* and begin the timestep. \| 0 \| \| barb_actin_no \| The current subunit number at the barbed end position. This allows actin agents to identify their position within the filament. \| 2 (if only a 3-subunit seed) \| \| point_actin_no \| The current subunit number at the pointed end position. This allows actin agents to identify their position within the filament. \| 0 (if only a 3-subunit seed) \| \| barb_dist \| A temporary variable holding the distance between the barbed end and its closest binding candidate. This is used to determine which candidate is the closest, to avoid multiple filaments (or ends) attempting to bind the same agent simultaneously. \| -1 \| \| point_dist \| A temporary variable holding the distance between the pointed end and its closest binding candidate. This is used to determine which candidate is the closest, to avoid multiple filaments (or ends) attempting to bind the same agent simultaneously. \| -1 \| \| requested_barb_id \| A temporary variable used to hold the agent ID attempting to bind this agent prior to binding confirmation. \| -1 \| \| requested_point_id \| A temporary variable used to hold the agent ID attempting to bind this agent prior to binding confirmation. \| -1 \| \| awaiting_confirmation \| A Boolean variable used to signify if an agent attempted to bind another agent during the current timestep. This is used as a function guard so that subsequent functions in the binding phase are only accessed by the appropriate agents. \| 0 \| \| elongation_allowed \| A Boolean variable used to signify when the agent represents a nucleus (0) or seed (1). \| 0 (1 if the filament was either salt nucleated or already present at the start of a simulation) \| \| binding_state \| A Boolean variable which shows if the agent is bound (1) or not bound (0) to Las17. \| 0 \|   **Table S5. The data class variables of FilamentAgents**. A list of all the agent-specific variables found within the FilamentAgent data class, their descriptions, and default values given during both creation of the initiation XML file and agent creation during nucleation. |
| --- | --- | --- | --- | --- | --- | --- | --- | --- | --- | --- | --- | --- | --- | --- | --- | --- | --- | --- | --- | --- | --- | --- | --- | --- | --- | --- | --- | --- | --- | --- | --- | --- | --- | --- | --- | --- | --- | --- | --- | --- | --- | --- | --- | --- | --- | --- | --- | --- | --- | --- | --- | --- | --- | --- | --- | --- | --- | --- | --- | --- | --- | --- | --- |

### 7.5. Function order

The order of functions executed during each simulation iteration is detailed at the bottom of the XML file as detailed within **Table S6**. Due to the parallel nature of FLAME GPU, a function acts upon all agents once it is called while numerous functions can be called simultaneously. These rounds of program calls are termed function layers, and each layer is iterated through sequentially (from 1 to 38) once per iteration of the simulation. Agents can also occupy either a resolved or unresolved state and these are given within the first column.

| \| **Layer number and states accessible (<X>)** \| **Functions called** \| **Brief overview of the layer’s function** \| \| --- \| --- \| --- \| \| 1  <resolved> \| reset_domains,  reset_platforms,  reset_filaments \| If the previous iteration was a timestep iteration, then agents return to the unresolved state. \| \| 2  <unresolved> \| move_free_platforms  move_free_filaments \| Peptides in priority block 1 and unbound filaments move. \| \| 3  <unresolved> \| update_moved_first_domains \| DomainAgents belonging to priority block 1 platforms update their vectors. \| \| 4  <unresolved> \| output_previously_moved_domains  update_domain_timer  update_platform_timer \| Moved domains output their locations and agents update their timer variables (using the global *ITERATION_PLUS_ONE* variable) for the guard loop of layer 5. \| \| 5  <unresolved> \| move_bound_platforms \| Peptides in the priority block equal to the current value of *ITERATION_PLUS_ONE* move using the location outputs of layer 4 to constrain their movements. \| \| 6  <unresolved> \| update_moved_domains  output_bound_filament_possitions \| DomainAgents belonging to platforms that moved in layer 5 update their vectors. All platforms which have moved this iteration and are bound to a filament output their location. \| \| 7  <unresolved> \| move_bound_filaments \| FilamentAgents which are bound to Las17 move using the outputs of layer 6. \| \| 8  <unresolved> \| progress_platforms  progress_domains \| A global guard loop will shift all domains and platforms into the resolved state (ready for a timestep) if all agents have moved since the last timestep. \| \| 9  <resolved> \| progress_tester \| FilamentAgents search for a synchronisation message from layer 9. If found, they prepare for a timestep. \| \| 10  <unresolved> \| progress_filaments \| All FilamentAgents prepared for a timestep are moved into the resolved state (ready for a timestep). \| \| 11  <unresolved> \| output_avalable_domains_for_filament \| Unbound DomainAgents of type 5 (actin) output their locations. \| \| 12  <resolved> \| filament_binding_requests \| FilamentAgents identify which type 5 DomainAgents they can bind using the locations given in layer 12. \| \| 13  <resolved> \| confirm_filament_binding_requests \| FilamentAgents communicate between one another to sort any conflicts between binding requests such that the same G-actin is not simultaneously bound by two different filaments. \| \| 14  <resolved> \| binding_filament \| Unbound DomainAgents of type 5 (actin) check if they were bound in layer 14. If so, they update their variables accordingly. \| \| 15  <resolved> \| output_avalable_domains_for_binding \| All unbound DomainAgents output their locations. \| \| 16  <resolved> \| domain_binding_requests \| All unbound DomainAgents identify which other DomainAgents they can bind using the locations given in layer 16. \| \| 17  <resolved> \| confirm_domain_binding_requests \| DomainAgents communicate between one another to sort any conflicts between binding requests to prevent any agent from being simultaneously bound by two or more other agents. \| \| 18  <resolved> \| register_bound_domains \| All unbound DomainAgents check if they were bound in layer 18. If so, they update their variables accordingly. \| \| 19  <resolved> \| update_binding_domain_priority_values \| DomainAgents sharing a platform_complex_id with another agent that interacted in layers 19 and 20 output their binding data. \| \| 20  <resolved> \| update_binding_platform_priority_values \| Binding data from layer 20 is sorted through for each PlatformAgent. This is used to generate new priorities within complexes. \| \| 21  <resolved> \| output_binding_platform_priorities \| Platforms from layer 21 update their complex_size variable and output this along with their ID and new priority. \| \| 22  <resolved> \| updating_binding_domain \| DomainAgents belonging to platforms in layer 22 use the output to update their priority and complex size variables. \| \| 23  <resolved> \| binding_report_requests \| All DomainAgents output their binding data. \| \| 24  <resolved> \| register_dimerisation \| Platforms check if they are now dimerised using the layer 23 message list. \| \| 25  <resolved> \| register_binds \| Platforms update their binding reporter variables and output these as a message. \| \| 26  <resolved> \| register_dimer_binds \| Domains update their binding context variables using the layer 25 message list. \| \| 27  <resolved> \| actin_unbinding_requests  filament_unbinding_requests  output_bound_actin_neighbours \| PlatformAgents and FilamentAgents check whether they can unbind filaments and actin domains respectively. If so, they update their variables. \| \| 28  <resolved> \| unbinding_actin_from_filament  unbinding_tracts_from_filament  unbinding_filament_from_Las17  domain_unbinding_requests \| Actin DomainAgents unbound in layer 27 update their variables. DomainAgents check if their can unbind their interaction partners. Cooperativity is calculated using the *output_bound_actin_neighbours* message list from layer 27. \| \| 29  <resolved> \| update_unbounded_actin_platforms  unbinding_dimers_from_filament  confirm_domain_unbinding_requests \| Parent PlatformAgents of the actin DomainAgents updated in layer 28 update their variables. Domain agents which could unbind during layer 28 communicate between one another so that only one agent/complex can unbind. Successfully unbinding agents update their variables. \| \| 30  <resolved> \| register_unbound_domains \| All bound DomainAgents check if they were unbound in layer 29. If so, they update their variables accordingly. \| \| 31  <resolved> \| register_for_priority_update \| DomainAgents which unbound in layers 29 and 30 output their binding data. \| \| 32  <resolved> \| update_unbinding_platform_priorities \| Binding data from layer 31 is sorted through for each PlatformAgent. This is used to generate new priorities within complexes. \| \| 33  <resolved> \| output_unbinding_platform_variables \| Platforms from layer 32 update their complex_size variable and output this along with their ID and new priority. \| \| 34  <resolved> \| update_unbinding_domain_priority_values \| DomainAgents belonging to platforms in layer 33 use the output to update their priority and complex size variables. \| \| 35  <resolved> \| binding_report_requests \| All DomainAgents output their binding data (same function as layer 23). \| \| 36  <resolved> \| register_dimerisation \| Platforms check if they are now dimerised using the layer 35 message list (same function as layer 24). \| \| 37  <resolved> \| register_binds \| Platforms update their binding reporter variables and output these as a message (same function as layer 25). \| \| 38  <resolved> \| register_dimer_binds  Las17_nucleation \| Domains update their binding context variables using the layer 37 message list (same function as layer 26). Las17 platforms sort through their binding data and if they identify an actin domain bound to PP1, PP3 and PP4 (from either themselves or their dimerised partner), they nucleate a new FilamentAgent. \| \| 39  <resolved> \| register_nucleation_tracts \| DomainAgents from Las17 platforms that nucleated in layer 38 update their variables accordingly. \| \| 40  <resolved> \| register_nucleation_dimer  register_nucleation_actin  auto_nucleation \| Las17 platforms which are dimerised to a platform that nucleated actin in layer 39 update their bound filament ID variable. Actin DomainAgents which nucleated in layer 38 update their variables accordingly. A single PlatformAgent nucleates a new Filament agent if permitted according to the rate of salt-mediated nucleation (mimicking salt nucleation in solution). \| \| 41  <resolved> \| update_nucleation_platforms  update_nucleating_domain_priority_values \| Parent PlatformAgents of actin DomainAgents that nucleated in layer 38 update their variables accordingly. DomainAgents belonging to complexes which nucleated actin in layer 38 output their binding data. \| \| 42  <resolved> \| update_nucleating_platform_priority_values \| Binding data from layer 41 is sorted through for each PlatformAgent. This is used to generate new priorities within complexes. \| \| 43  <resolved> \| output_nucleating_platform_priorities \| Platforms from layer 42 update their complex_size variable and output this along with their ID and new priority. \| \| 44  <resolved> \| updating_nucleating_domain \| DomainAgents belonging to platforms in layer 43 use the output to update their priority and complex size variables. \|   **Table S6. Function layers**. A list of all the function layers detailed in the XML model file, the functions contained within and the general purpose of the layer in the context of the simulation. |
| --- | --- | --- | --- | --- | --- | --- | --- | --- | --- | --- | --- | --- | --- | --- | --- | --- | --- | --- | --- | --- | --- | --- | --- | --- | --- | --- | --- | --- | --- | --- | --- | --- | --- | --- | --- | --- | --- | --- | --- | --- | --- | --- | --- | --- | --- | --- | --- | --- | --- | --- | --- | --- | --- | --- | --- | --- | --- | --- | --- | --- | --- | --- | --- | --- | --- | --- | --- | --- | --- | --- | --- | --- | --- | --- | --- | --- | --- | --- | --- | --- | --- | --- | --- | --- | --- | --- | --- | --- | --- | --- | --- | --- | --- | --- | --- | --- | --- | --- | --- | --- | --- | --- | --- | --- | --- | --- | --- | --- | --- | --- | --- | --- | --- | --- | --- | --- | --- | --- | --- | --- | --- | --- | --- | --- | --- | --- | --- | --- | --- | --- | --- | --- | --- | --- | --- |

## 8. Function file

The code for each function is written using C in the function file. Guard loops for these functions are defined in the XML file, however, to maintain reading coherency, these will be described alongside their respective functions below.

### 8.1 Global Functions

Wherever possible, variables are defined globally. Global variables can only be changed during the CPU code at the end of each time step. They can be accessed, though not edited, by all agent functions where they are called. These variables are used to hold information that is specific to the simulation but not specific agents. This reduces the memory requirement during model construction as shared agent variables can be allocated as a global instead.

boundryCheck

This function accepts an agent’s positional vector as an argument and checks whether it is located outside of the bounds set by the *XMAX*, *XMIN*, *YMAX*, *YMIN*, *ZMAX*, or *ZMIN* global variables. It is a periodic boundary: if any vector values exist outside the bounds set, they are changed to a value at the opposite axis of the simulation space. When running in *In Vivo* mode, the upper and lower *z* axis boundaries are replaced with elastic conditions to reproduce the effect of a membrane.

movement_calc

Movements are calculated in polar coordinates to ensure that agents move at the same rate in each direction. This function accepts a movement distance, theta angle, phi angle, and origin vector and returns the new coordinates as a float3 vector object.

random_angle

The random_angle function multiplies a random uniform number argument between 0 and 1 with 2π to generate a random polar angle. This simple global function was implemented to help visually clean up the code making bug fixing and code modifications easier.

random_distance

Using the same logic as random_angle, this function helps to make code more concise when generating random distances. It accepts the minimum and maximum distance permitted along with a random uniform number between 0 and 1. A simple equation then calculates a random distance value between these bounds.

### 8.2 Layer 1: Timestep reset

| Agent’s subject | >currentState  nextState> | Function guard | >Message input  Message output> |
| --- | --- | --- | --- |
| Function: reset_domains | | | |
| DomainAgent | >Resolved  Unresolved> |  |  |
| Function: reset_platforms | | | |
| PlatformAgent | >Resolved  Unresolved> |  |  |
| Function: reset_filaments | | | |
| FilamentAgent | >Resolved  Unresolved> |  |  |

Layer 1 is only executed following a successful timestep to move all resolved agents into the unresolved state ready for a new timestep preparation. These functions uncouple the next iteration from the next timestep as agents must occupy a resolved state to access timestep-specific functions. State reset occurs at the start of an iteration, rather than the end, so that the data recording CPU code is executed whilst agents are in the resolved state (indicative of a successful timestep).

### 8.3 Layer 2: Moving nondependent agents

| Agent’s subject | >currentState  nextState> | Function guard | >Message input  Message output> |
| --- | --- | --- | --- |
| Function: move_free_platforms | | | |
| PlatformAgent | >Unresolved  Unresolved> | Must be in *priority block 1* and *awaiting_movement_platform* = 1 | moved_domain_first> |
| Function: move_free_filaments | | | |
| FilamentAgent | >Unresolved  Unresolved> | Must be unbound to a platform agent and *awaiting_movement_filament* = 1 |  |

Platform agents and Filament agents move by a Brownian motion random walk, based on the Matplotlib Animated 3D random walk. Platforms first move by generating random phi and theta angles and generating random distances between the five domain vectors that may be associated with a DomainAgent. These agents then use the *random_distance* global function by indexing into the D*(1-4)*MIN and D*(1-4)*MAX global variable arrays using their agent *species* variables. Platforms move translationally using the *centre* vector and then calculate the positions of their five domain vectors (using the random distances calculated earlier) moving outwards. For example, vector2 and vector3 calculate their movement away from the centre vector while vector1 and vector4 calculate their movement away from vector2 and vector3 respectively. Once successfully moved, *awaiting_movement_platform* is set to 0 to prevent repeated access to the function and a message is outputted containing domain locations, the ID of any homodimerised partner platform using the *dimerised_partner_id* variable, and the ID of any bound filament using the *filament_bound* variable (calculated at the end of timesteps). The latter two variables are outputted here as it is the most efficient function to disseminate the information to their child domains.

FilamentAgents first translate using the *centre* vector. They then generate a random barb-point axis and execute a movement step (along this axis) for both the *barb* and *point* vectors with distances equal to half the length of the filament. Finally, *awaiting_movement_filament* is set to 0 to prevent repeated access to the function.

### 8.4 Layer 3: Updating nondependent domain agents

| Agent’s subject | >currentState  nextState> | Function guard | >Message input  Message output> |
| --- | --- | --- | --- |
| Function: update_moved_first_domains | | | |
| DomainAgent | >Unresolved  Unresolved> | Must be in *priority block 1* and *awaiting_movement_domain* = 1 | >moved_domain_first |

Domain agents in *priority block 1* search for their linked PlatformAgent in the message output of layer 2 and update their vector location and dimerisation status (stored using the dimerised variable) from the message according to their *domain_index* variable. For dimerisation domains, if the filament ID number received has a value greater than -1 (i.e., the parent platform is bound to a filament) then *unbind_dimer_block* is set to 1 to prevent the protein dissociating from a dimerised partner. This is important as filaments are often bound by the domains belonging to both parent platforms of a Las17 dimer and navigating this would prove computationally challenging and resource intensive. Instead, we only allow Las17 dimers to dissociate following the dissociation of any bound filaments. Agents finish by updating *awaiting_movement_domain* to 0.

### 8.5 Layer 4: Preparation for dependant movement

| Agent’s subject | >currentState  nextState> | Function guard | >Message input  Message output> |
| --- | --- | --- | --- |
| Function: output_previously_moved_domains | | | |
| DomainAgent | >Unresolved  Unresolved> | *awaiting_movement_domain* = 0 | previously_moved> |
| Function: update_domain_timer | | | |
| DomainAgent | >Unresolved  Unresolved> |  |  |
| Function: update_platform_timer | | | |
| PlatformAgent | >Unresolved  Unresolved> |  |  |

DomainAgents which have moved since the last timestep (*awaiting_movement_domain* = 0) output their location and binding data to help with the movement of lower priority blocks. DomainAgents and PlatformAgents update their agent turn counter variables using the *update_domain_timer* and *update_platform_timer* functions to a value equal to the global *ITERATION_PLUS_ONE* variable. This is for use in the guard loops of subsequent layers.

### 8.6 Layer 5: Moving dependent agents

| Agent’s subject | >currentState  nextState> | Function guard | >Message input  Message output> |
| --- | --- | --- | --- |
| Function: move_bound_platforms | | | |
| PlatformAgent | >Unresolved  Unresolved> | *awaiting_movement_domain* = 1 and *priority* equals the *platform_turn_counter* | >previously_moved  moved_domain> |

Layer 5 focuses on moving platforms with a priority greater than 1. It does this by moving agents with a priority equal to the *iteration number + 1*. This means that priority block 2 will first execute *move_bound_platforms* on the iteration directly following a successful timestep (iteration 1). The subsequent iterations will execute iteratively higher priority blocks (e.g., block 3, block 4 etc.) until all PlatformAgents have successfully moved and the timestep can begin.

This function is one of the longest and most complex. It starts similar to *move_free_platforms* by generating random angles and distances. However, the function also begins by generating an array of five, float3 vector objects with all coordinates set at zero. Agents then run through all the message outputs from layer 4 to check if any moved domains (those which have successfully moved since the timestep) are bound to any of their domain locations. If so, the zeroed coordinate for that vector is set at the same value as the bound domain.

The function enters a complex series of nested “if” statements which check which domain vectors in the coordinate array are zeroed to determine how the movement of the agent is constrained. For example, if vector2 and vector4 are both bound to a higher priority agent, then the position of vector3 is physically constrained by its neighbours as movement may break spatial coherence.

In summary of the process, movements are performed using one neighbour as the movement origin. The distance between the new position and the second neighbour is measured to ensure that this distance is not greater than allowed. This occurs inside a “while loop” until either a position is found which satisfies all spatial constraints, or the number of attempts is greater than the *MAX_BOUNDED_ATTEMPTS* global variable. In the case of the latter, the position of the constrained domain is set equidistant from both neighbours to prevent excessive/infinite looping. The code below shows how this scenario is solved (**code #1**).

| **Code #1** |
| --- |
| 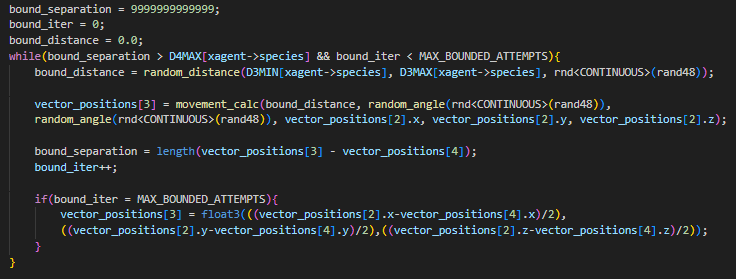 |

In the case of bound domains flanking several vectors (e.g., vector2 and vector5 bound, constraining both vector3 and vector4), an iterative approach is taken with multiple “while loops”. This code differs only slightly from what is explained above and is executed until the requirements for complete spatial coherence are achieved.

FLAME GPU 1 does not allow the calling of random numbers from within global functions. This unfortunate drawback resulted in a large degree of code repetition throughout this function. However, all possible binding scenarios were independently tested, and the function performs as expected for each of them. Following completion of the function a message is outputted containing domain locations, the ID of any homodimerised partner platform using the *dimerised_partner_id* variable, and the ID of any bound filament using the *filament_bound* variable (calculated at the end of timesteps). As with layer 2, the latter two variables are outputted here as it is the most efficient function for higher priority value platforms to disseminate the information to their child domains.

### 8.7 Layer 6: Updating dependent domain agents

| Agent’s subject | >currentState  nextState> | Function guard | >Message input  Message output> |
| --- | --- | --- | --- |
| Function: update_moved_domains | | | |
| DomainAgent | >Unresolved  Unresolved> | *platform_priority* equals the *platform_turn_counter* | >moved_domain |
| Function: output_bound_filament_possitions | | | |
| PlatformAgent | >Unresolved  Unresolved> | Las17 platform that must be bound to an actin filament | bound_filament_vectors> |

DomainAgents belonging to platforms that moved in layer 5 update their vectors and dimerisation status (stored using the dimerised variable). Using the same code as described in *update_moved_first_domains* (layer 3), dimerisation domains also set the value of *unbind_dimer_block* to 1 should the filament ID received have a value greater than -1

PlatformAgents bound to actin filaments (in a manner only achievable by Las17 objects that nucleated said filament) output their vectors. This is because bound FilamentAgents always default to the vector position of their bound Las17 platform.

### 8.8 Layer 7: Updating bound filament locations

| Agent’s subject | >currentState  nextState> | Function guard | >Message input  Message output> |
| --- | --- | --- | --- |
| Function: move_bound_filaments | | | |
| FilamentAgent | >Unresolved  Unresolved> | Agent must be bound to a nucleating platform | >bound_filament_vectors |

The vector positions given in layer 6 are iterated through by bound actin filaments. If their id number matches, then the filament will move to the coordinates of its bound Las17 platform.

### 8.9 Layer 8: Timestep progression: Peptides

| Agent’s subject | >currentState  nextState> | Function guard | >Message input  Message output> |
| --- | --- | --- | --- |
| Function: progress_platforms | | | |
| PlatformAgent | >Unresolved  Resolved> | **All** PlatformAgents must have moved since the last timestep (awaiting_movement_platform = 0) | record_ticket> |
| Function: progress_domains | | | |
| DomainAgent | >Unresolved  Resolved> | **All** DomainAgents must have moved since the last timestep (awaiting_movement_domain = 0) |  |

FLAME GPU 1 allows the use of global guard loops that only allow the function to be executed if all agents of the same type satisfy the same condition. In the case of *progress_platforms*, all PlatformAgents must have *awaiting_movement_platform* value of “0” indicating that they have all completed the movement phase. The function releases a message containing the value of “1” before moving all agents into the resolved state ready for a new timestep. *progress_domains* is executed at the same time because, if all platforms have moved, all domains must also have moved (every domain is linked to a platform). This function moves DomainAgents into a resolved state.

### 8.10 Layer 9: Timestep progression: Filaments 1

| Agent’s subject | >currentState  nextState> | Function guard | >Message input  Message output> |
| --- | --- | --- | --- |
| Function: progress_tester | | | |
| FilamentAgent | >Unresolved  Unresolved> | *platform_priority* equals the *platform_turn_counter* | >record_ticket |

It is critical that all agent types enter the timestep phase together to maintain synchronicity. Therefore, *progress_tester* searches the layer 8 message list for values of “1” and, if so, sets the value of the FilamentAgent variable *execute_procession* to “1”. Finding this value in the layer 8 output indicates that PlatformAgents have executed the progress_platforms function and have thus entered a resolved state.

### 8.11 Layer 10: Timestep progression: Filaments 2

| Agent’s subject | >currentState  nextState> | Function guard | >Message input  Message output> |
| --- | --- | --- | --- |
| Function: progress_filaments | | | |
| FilamentAgent | >Unresolved  Resolved> | **All** FilamentAgents must an *execute_procession* value of “1” |  |

FilamentAgents enter a resolved state once all agent classes are synchronised ready to begin the timestep phase (via holding *execute_procession* values of “1”).

### 8.12 Layer 11: Outputting actin monomers for filaments

| Agent’s subject | >currentState  nextState> | Function guard | >Message input  Message output> |
| --- | --- | --- | --- |
| Function: output_avalable_domains_for_filament | | | |
| DomainAgent | >Resolved  Resolved> | Agents must be of type 5 (actin) and not part of a filament | avalable_actin_location> |

Type 5 DomainAgents that are not incorporated into a filament (G-actin) which are either unbound (monomeric) or bound to a type 8 DomainAgent (bound to a Ysc84 YAB domain) are possible binding candidates for actin filaments. Therefore, in preparation for the polymerisation phase, these agents output their location as a message.

### 8.13 Layer 12: Actin polymerisation: requests

| Agent’s subject | >currentState  nextState> | Function guard | >Message input  Message output> |
| --- | --- | --- | --- |
| Function: filament_binding_requests | | | |
| FilamentAgent | >Resolved  Resolved> |  | >avalable_actin_location  priority_actin_ticket> |

FilamentAgents search the layer 11 message list for G-actin DomainAgents close enough to bind, at either barbed or pointed end. It iterates through all the messages and identifies the closest agents within the interaction radius (if there are any) to both the barbed and pointed end. Binding data is then saved in the temporary *requested* agent variables for sorting. If the FilamentAgent is still classified as a nucleus and not a seed (using the *elongation_allowed* agent variable), then binding of a fourth subunit will use the *SIDE_PON* variable to account for the possible linear tandem nucleating mechanism.

### 8.14 Layer 13: Actin polymerisation: confirmation

| Agent’s subject | >currentState  nextState> | Function guard | >Message input  Message output> |
| --- | --- | --- | --- |
| Function: confirm_filament_binding_requests | | | |
| FilamentAgent | >Resolved  Resolved> | The agent must have found a prospective binding partner in layer 12 | >priority_actin_ticket  actin_ticket> |

This function identifies which interactions are approved should any DomainAgents be the simultaneous target of multiple filaments. If two agents are attempting to bind to the same target, the closer one is approved. Interactions approved for binding then occur and the appropriate variables are updated. FilamentAgents that are defined as nuclei can update their *elongation_allowed* variable to redefine themselves as a seed should the size of the complex be five or greater.

### 8.15 Layer 14: Update polymerised monomers

| Agent’s subject | >currentState  nextState> | Function guard | >Message input  Message output> |
| --- | --- | --- | --- |
| Function: binding_filament | | | |
| DomainAgent | >Resolved  Resolved> | Agents must be of type 5 (actin) and not part of a filament | >actin_ticket |

All unbound actin DomainAgents iterate through the messages of layer 14. If their ID is identified, then the agent will update to a bound status and rewrite its variables accordingly.

### 8.16 Layer 15: Outputting unbound domains

| Agent’s subject | >currentState  nextState> | Function guard | >Message input  Message output> |
| --- | --- | --- | --- |
| Function: output_avalable_domains_for_binding | | | |
| DomainAgent | >Resolved  Resolved> | Agents must be unbound | avalable_domain_location> |

All unbound DomainAgents output their location and relevant dimerisation variables (e.g., cooprative_dimerisation_PP1, etc.) ready for the binding phase. They also send a random number within the message for use within the binding probability checks.

### 8.17 Layer 16: Domain binding: requests

| Agent’s subject | >currentState  nextState> | Function guard | >Message input  Message output> |
| --- | --- | --- | --- |
| Function: domain_binding_requests | | | |
| DomainAgent | >Resolved  Resolved> | Agents must be unbound | >avalable_domain_location  priority_domain_ticket> |

Each unbound domain agent then assesses the binding potential for all other unbound domains. This code is derived from layer 12 and likewise searches for DomainAgents that are within the interaction radius and with a random number (submitted in the layer 15 message list) less than the binding probability. Binding probabilities for specific interactions can be obtained from multiple possible global arrays depending upon the dimerisation status of their parent platform and whether the agent executing the function is a dimerisation domain. Non-dimerised, protein motif domains use the *BIND_PROB* global array.

FLAME GPU 1 does not support 2D arrays. However, the *BIND_PROB* global variable (along with the other 196-unit arrays) reproduce the effect in a 1D array by combining both prospective interaction partners to get a unique index.

Dimerised protein motif domains use the *BIND_PROB_DIMERISED* global array, which may contain alternative probabilities that reflect the dimerisation status (e.g., actin and Las17 interactions have a higher probability in the *BIND_PROB_DIMERISED* array than the *BIND_PROB* array). On the other hand, dimerisation domains obtain their binding probability from the DIMER_BIND_PROB global array using their *platform_species* variable. This array holds the probability of homodimerisation for each simulated protein when their binding radii overlap.

The model allows for dimerisation cooperativity such that actin being bound at one tract may increase the dimerisation affinity assuming that the equivalent tract of the prospective dimerisation partner is free to interact with the actin. Theoretically, if the other tract is bound by another agent, then it would be unavailable to actin and thus unable to receive the cooperative benefit when dimerising. This process was regulated by making the dimerisation domains of Las17 sum their *cooprative_dimerisation* variables with those of the prospective binding partner. These summations are undertaken for each actin-binding polyproline tract individually (i.e. (*xagent->cooprative_dimerisation_PP1* + *message->cooprative_dimerisation_PP1*), (*xagent->cooprative_dimerisation_PP3* + *message->cooprative_dimerisation_PP3*), and (*xagent->cooprative_dimerisation_PP4* + *message->cooprative_dimerisation_PP4*)). Because *cooprative_dimerisation* variables have a value of 0 if unbound, 1 if SH3-bound and 10 if actin-bound, then if any summations equal to 10, then at least one polyproline is tract is actin bound whilst its equivalent tract on the other Las17 is unbound. In this case, the dimerisation domain searches the COOP_DIMER_BIND_PROB global array using its *platform_species* variable to obtain a binding probability.

### 8.18 Layer 17: Domain binding: confirmation

| Agent’s subject | >currentState  nextState> | Function guard | >Message input  Message output> |
| --- | --- | --- | --- |
| Function: confirm_domain_binding_requests | | | |
| DomainAgent | >Resolved  Resolved> | Agents must have identified a possible binding partner in layer 17 | >priority_domain_ticket  confirmed_domain_ticket> |

This function is derived from layer 13 and checks to identify which interactions are approved should more than one DomainAgent attempt to bind the same target agent simultaneously.

An important consideration for using functions that employ parallel binding within the same agent class is that both agents may attempt to simultaneously bind each other. To avoid this and maintain unidirectional binding, agents can only attempt to bind domains that generated a smaller random number during layer 16 for calculating the binding probability (stored using the *probability_request* variable). To allow for efficient priority recalculation, only one DomainAgent per complex is allowed to bind during this layer.

### 8.19 Layer 18: Update bound domains

| Agent’s subject | >currentState  nextState> | Function guard | >Message input  Message output> |
| --- | --- | --- | --- |
| Function: register_bound_domains | | | |
| DomainAgent | >Resolved  Resolved> | Agents must be unbound | >confirmed_domain_ticket  binding_domain_update_priority> |

All unbound DomainAgents iterate through the messages of layer 17. If their ID is identified, then the agent will update to a bound status and rewrite its variables accordingly. Following a binding event, the network of platform interactions and thus composition of a complex may change. Therefore, priority values must be redetermined and this begins by outputting messages containing the *complex_id* value for both interaction partners.

### 8.20 Layer 19: Binding priority update: interaction network

| Agent’s subject | >currentState  nextState> | Function guard | >Message input  Message output> |
| --- | --- | --- | --- |
| Function: update_binding_domain_priority_values | | | |
| DomainAgent | >Resolved  Resolved> |  | >binding_domain_update_priority binding_platform_update_priority> |

The first stage of redetermining priority values is formulating a comprehensive view of all interactions within a complex. Therefore, during *update_binding_domain_priority_values* all DomainAgents iterate through the messages of layer 18 and search for their *complex_id*. Domains which successfully match their variables with either of the two *complex_id* values set their *complex_id* to that of the dominant binding partner within the layer 17 interaction. They then output their binding information and both *complex_id* values from their input message as an outgoing message. DomainAgents which initiated the bind in layers 16/17 are given the “priority_prime” message value of 1.

### 8.21 Layer 20: Binding priority update: determining priority

| Agent’s subject | >currentState  nextState> | Function guard | >Message input  Message output> |
| --- | --- | --- | --- |
| Function: update_binding_platform_priority_values | | | |
| PlatformAgent | >Resolved  Resolved> |  | >binding_platform_update_priority binding_complex_id_counter> |

Redetermining priority values is one of the most complex and lengthy functions of this simulation. A flowchart detailing the steps of this function is shown in **Figure S14**. It begins by iterating through the message list of layer 19. Entries with complex ID values matching the *complex_id* of the agent (either of the two given in the message) are identified. Functions in FLAME GPU 1 cannot read a message more than once. Therefore, the platform ids and their bound PlatformAgents are saved for each matching message in a local variable list. This first message loop also initiates the *past_list* variable with the *platform_id* entry of the dominant binding partner within the layer 17 interaction involving this complex. If this ID matches the ID of the agent executing the function, then the function is ended, and *priority* value set to 1. Agents which never identified their complex_id within the message list also end the function at this point.

All agents continuing the function (via a local variable value of *update_required == 1*) are platforms associated with one of the binding partners. To identify their ultimate priority values, a “while loop” is executed which walks through the complex by identifying all platforms bound to agents within the *past_list*. These newly identified agents are saved in the *current_list*. At the end of the while loop iteration, *past_list* is cleared and repopulated by the contents of *current_list* (*current_list* is subsequently cleared). This platform-by-platform walking continues until the agent executing the function identifies its own ID number in the *current_list*. The number of “while loop” iterations required to reach this conclusion (+ 1) defines the priority value.

| 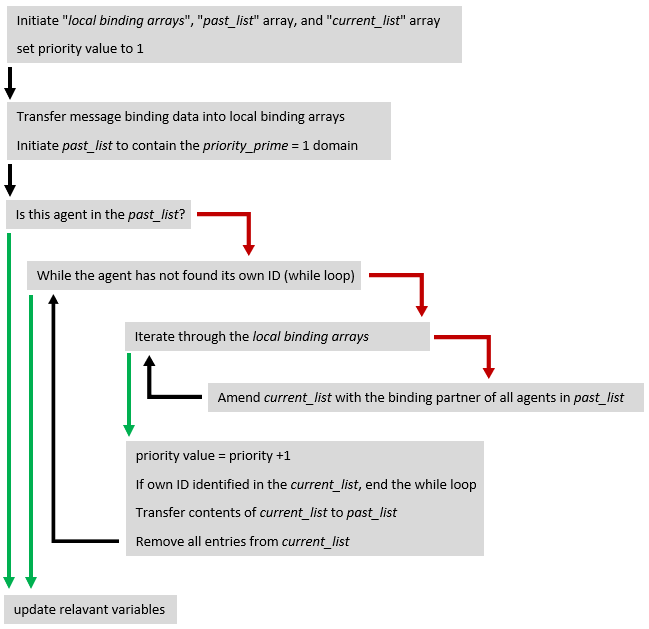 |
| --- |
| **Figure S14. Simplified flowchart of priority recalculation following the binding phase**. Arrows show the direction of code with red paths being followed when a condition of a statement is not met, green when the condition is met, and black being followed regardless. Each agent essentially “walks” along the complex (starting from priority prime which is described in layer 19) until its ID number is identified. The agent priority is incremented every time the while loop is completed which results in the agent identifying its priority position relative to priority prime. |

### 8.22 Layer 21: Binding priority update: disseminating priority values

| Agent’s subject | >currentState  nextState> | Function guard | >Message input  Message output> |
| --- | --- | --- | --- |
| Function: output_binding_platform_priorities | | | |
| PlatformAgent | >Resolved  Resolved> | Agents must have updated their priority values in layer 20 | >binding_complex_id_counter binding_final_update> |

PlatformAgents that redetermined their priority values this timestep iterate through the output of layer 20 to count how many PlatformAgents are located within their complex. This value is then saved in the *complex_size* variable. Agents then output a message containing information regarding the complex, including size and priority.

### 8.23 Layer 22: Binding priority update: updating domains

| Agent’s subject | >currentState  nextState> | Function guard | >Message input  Message output> |
| --- | --- | --- | --- |
| Function: updating_binding_domain | | | |
| DomainAgent | >Resolved  Resolved> | Agents must have output a message during layer 19 | >binding_final_update |

DomainAgents that belong to PlatformAgents that redetermined priority and size values iterate through the message list of layer 21 until they identify a message from their parent platform. *platform_complex_size* and *platform_priority* variables are updated accordingly.

**8.24 Layer 23: Updating DomainAgent context 1: uploading data**

| Agent’s subject | >currentState  nextState> | Function guard | >Message input  Message output> |
| --- | --- | --- | --- |
| Function: binding_report_requests | | | |
| DomainAgent | >Resolved  Resolved> |  | upload_partners> |

The context of DomainAgents, including the *cooprative_dimerisation* and *actin_within_dimer* variables may change following the binding stage and so must be updated. This begins by every DomainAgent outputting a message containing its binding data.

### 8.25 Layer 24: Updating DomainAgent context 1: reassessing dimerisation status

| Agent’s subject | >currentState  nextState> | Function guard | >Message input  Message output> |
| --- | --- | --- | --- |
| Function: register_dimerisation | | | |
| PlatformAgent | >Resolved  Resolved> | Only proteins which have the possibility to form homodimers | >upload_partners |

PlatformAgents must first identify whether they are now homodimerised before sorting through the binding data from layer 23. This is because the data processing requires input from both the agents’ child domains and the child domains of any dimerised partner. Therefore, platforms must identify any newly dimerised partner platform prior to the sorting function. PlatformAgents read the messages from their child dimerisation domains and update their *dimerised_partner_id* to the *bound_platform* variable of the child.

### 8.26 Layer 25: Updating DomainAgent context 1: data processing

| Agent’s subject | >currentState  nextState> | Function guard | >Message input  Message output> |
| --- | --- | --- | --- |
| Function: register_binds | | | |
| PlatformAgent | >Resolved  Resolved> |  | >upload_partners  dimerisation_updator_list> |

PlatformAgents run through the binding data messages from layer 23 having updated their dimerisation status via the previous layer. This allows platforms to extract the binding data of their child domains along with the child domains of any dimerised partner. This is then used to set the *vec_binding_partner* and *vec_dimer_partner* reporter variables representing each of the five possible binding motifs that can be linked to the platform.

### 8.27 Layer 26: Updating DomainAgent context 1: informing the relevant domains

| Agent’s subject | >currentState  nextState> | Function guard | >Message input  Message output> |
| --- | --- | --- | --- |
| Function: register_dimer_binds | | | |
| DomainAgent | >Resolved  Resolved> | Only proteins which have the possibility to form homodimers | >dimerisation_updator_list |

DomainAgents read the output messages of layer 25. Variables which allow domains to better understand their environmental context (e.g., what is bound at their equivalent position by any dimerised platforms etc.) by updating the dimer_adjacent_bound_type, actin_within_dimer, and cooprative_dimerisation variables.

### 8.28 Layer 27: Unbinding: filament requests

| Agent’s subject | >currentState  nextState> | Function guard | >Message input  Message output> |
| --- | --- | --- | --- |
| Function: actin_unbinding_requests | | | |
| FilamentAgent | >Resolved  Resolved> |  | freed_actins> |
| Function: filament_unbinding_requests | | | |
| PlatformAgent | >Resolved  Resolved> | Platform must be bound to an actin filament which it nucleated | freed_seeds> |
| Function: output_bound_actin_neighbours | | | |
| DomainAgent | >Resolved  Resolved> | Must be bound to a type 5 domain whilst itself being type 0-4 (Las17) | outputting_cooprativity> |

The barbed and pointed end of FilamentAgents check whether they can release an actin agent. If so, a message is written which contains the released subunit numbers. Subunit unbinding probabilities for filaments defined as nuclei rather than seeds (*elongation allowed* = 0) take their subunit unbind probability from the *SIDE_POFF* global variable. If the number of subunits (size variable) drops below “3”, then the function returns “death” which removes the agent from the simulation. The remaining subunits are then also released in the output message.

In *filament_unbinding_requests*, platforms bound to actin filaments (nucleating Las17s) compare a random number against the probability of unbinding. If the random number is lower, the filament’s ID is given as a message and *seed_id* is set to “-1”.

DomainAgents representing the Las17 motifs (types 0-4) which are also bound to actin agents release their binding details and *domain_index* value as a message for later use in calculating cooperativity.

### 5.6.29 Layer 28: Unbinding: domain requests and actin unbinding

| Agent’s subject | >currentState  nextState> | Function guard | >Message input  Message output> |
| --- | --- | --- | --- |
| Function: unbinding_actin_from_filament | | | |
| DomainAgent | >Resolved  Resolved> | Must be incorporated into an actin filament (i.e., F-actin) | >freed_actins  f_to_g_ticket> |
| Function: unbinding_tracts_from_filament | | | |
| DomainAgent | >Resolved  Resolved> | Domain must be bound to an actin filament which it nucleated | >freed_seeds |
| Function: unbinding_filament_from_Las17 | | | |
| FilamentAgent | >Resolved  Resolved> |  | >freed_seeds |
| Function: domain_unbinding_requests | | | |
| DomainAgent | >Resolved  Resolved> | Agent must be bound | >outputting_cooprativity  freed_domains> |

DomainAgents representing F-actin check the *freed_actins* message list of layer 27. Agents that identify themselves unbind from the filament, and make a random movement with a distance equal to the interaction radius.

Filament-bound DomainAgents belonging to the nucleating tracts of Las17 (bm2, bm4, and bm5) read the *freed_actins* message list in search of themselves. If successfully located, the domains unbind the filament and update their variables accordingly.

FilamentAgents search the *freed_seeds* message list for themselves. If successfully located, filaments unbind from the nucleating platform and update their variables accordingly.

DomainAgents in *domain_unbinding_requests* check whether they can release their bound agent. The “probability of unbinding” is taken from the appropriate 196-unit array. Non-dimerised binding motif domains use the UNBIND_PROB array while dimerised binding motif domains use the UNBIND_PROB_DIMERISED. Dimerisation domains access the appropriate 6-unit global array using their platform_species variable as an index. Las17 homodimers which have bound an actin agent are assumed to dissociate with a lower rate because of cooperativity. Dimerisation domains which have an actin present within either the parent platform or the platform of the dimerised partner use the COOP_DIMER_UNBIND_PROB. Otherwise, DIMER_UNBIND_PROB is accessed to obtain the unbinding probability of the dimer.

The chosen unbinding probability is then divided by the appropriate cooperativity global variable if iteration through the layer 27 message list identifies an adjacent actin-binding motif bound to an actin agent. bm2 and bm4 bound use *PP1_PP3_COOPERATIVITY* whilst bm4 and bm5 bound use *PP3_PP4_COOPERATIVITY*. Agents that are capable of unbinding do not do so immediately. The reasoning for this is that multiple simultaneous changes to complexes can disrupt the method of priority redetermination. Therefore, these agents only update a single variable (*probability_request*) to hold the random number value generated during the function. A message is output containing this number along with identifiable features of the agent.

### 8.30 Layer 29: Unbinding: confirmation

| Agent’s subject | >currentState  nextState> | Function guard | >Message input  Message output> |
| --- | --- | --- | --- |
| Function: update_unbounded_actin_platforms | | | |
| PlatformAgent | >Resolved  Resolved> | Agents must be of species 1 (Las17) | >f_to_g_ticket |
| Function: unbinding_dimers_from_filament | | | |
| PlatformAgent | >Resolved  Resolved> | The platform is bound to a filament | >freed_seeds |
| Function: confirm_domain_unbinding_requests | | | |
| DomainAgent | >Resolved  Resolved> | The agent must have been in layer 28 | >freed_domains  confirmed_freed_domains> |

The parent PlatformAgents of actin agents which were released from filaments during layer 28 identify themselves using the *f_to_g_ticket* layer 28 message list and update their vector locations accordingly.

PlatformAgents check the *freed_seeds* message list for references to their bound filament. If there is a match, the *filament_bound* variable is set to -1. This function exists to update the dimerisation partners of a Las17 agent which unbound their actin filament during layer 27.

DomainAgents that are successfully capable of unbinding this timestep iterate through the message list of layer 28. If no other agents within the same complex (and with a lower random number probability) are identified, then the agent will continue with the unbind, update the appropriate variables, and output a message. Agents which identified a lower probability random number within the same complex do not unbind. This ensures that more than one unbinding event never occurs within the timestep for the sample complex. The probability of unbinding (even for weak interactions) is sufficiently low that the chance of two unbinding events being simultaneously attempted within the same complex is extremely low.

### 8.31 Layer 30: Unbinding: domain unbinding

| Agent’s subject | >currentState  nextState> | Function guard | >Message input  Message output> |
| --- | --- | --- | --- |
| Function: register_unbound_domains | | | |
| DomainAgent | >Resolved  Resolved> | Agent must be bound | >confirmed_freed_domains  priority_registration> |

Bound DomainAgents read the layer 29 unbind message list for references to themselves. Agents which succeed in identifying themselves, unbind and change their variables accordingly.

### 8.32 Layer 31: Unbinding priority update: interaction network

| Agent’s subject | >currentState  nextState> | Function guard | >Message input  Message output> |
| --- | --- | --- | --- |
| Function: register_for_priority_update | | | |
| DomainAgent | >Resolved  Resolved> |  | >priority_registration  platform_priority_update> |

The unbinding phase then enters a series of priority determining functions similar to the binding phase. This begins with the *register_for_priority_update* function in which DomainAgents identify whether they are within the same complex as agents that have unbound this timestep. Agents that can identify themselves as one of the unbinding domains additionally identify themselves with a “partner number” (“1” for the dominant agent which initiated the unbind and “2” for the recipient of the unbind request in layer 30). Two new complex IDs are chosen using the ID of the dominant agent for *new_complexOne_id* and the ID of the recipient as *new_complexTwo_id*. Agents package this information, along with their binding data, as a message.

### 8.33 Layer 32: Unbinding priority update: determining priority

| Agent’s subject | >currentState  nextState> | Function guard | >Message input  Message output> |
| --- | --- | --- | --- |
| Function: update_unbinding_platform_priorities | | | |
| PlatformAgent | >Resolved  Resolved> |  | >platform_priority_update  complex_size_count > |

This code is highly derivative of the function that redetermined priorities following binding. However, it contains the additional complexity of three distinct outcomes that cannot be pre-determined. The first is that the complex may not break into two as additional interlinking domains may be present. Furthermore, each platform involved has two potential complexes (and thus priority values) they may be located within (**Figure S15**).

Like during the binding phase, dynamic lists are constructed that “walk” through the complex from an origin. However, two lists are developed during this function with one “walking out” from the dominant unbind partner whilst the second “walks out” from the unbind recipient. These two list constructions are also linked to their own priority counter. Three possible outcomes may result from a PlatformAgent executing this function.

First, is that both walking lists “meet up” and connect via the same platform. This implies that additional interdomain interactions are maintaining the structural complex. During this case, priority values taken from the binding recipient walk are applied to all platforms within the complex.

Second, both lists continue until completion without “meeting up” and the platform identifies itself within the binding recipient list. Here, the agent accepts the binding priority value from the binding recipient walk and complex ID of *new_complexTwo_id* (layer 31 message).

| 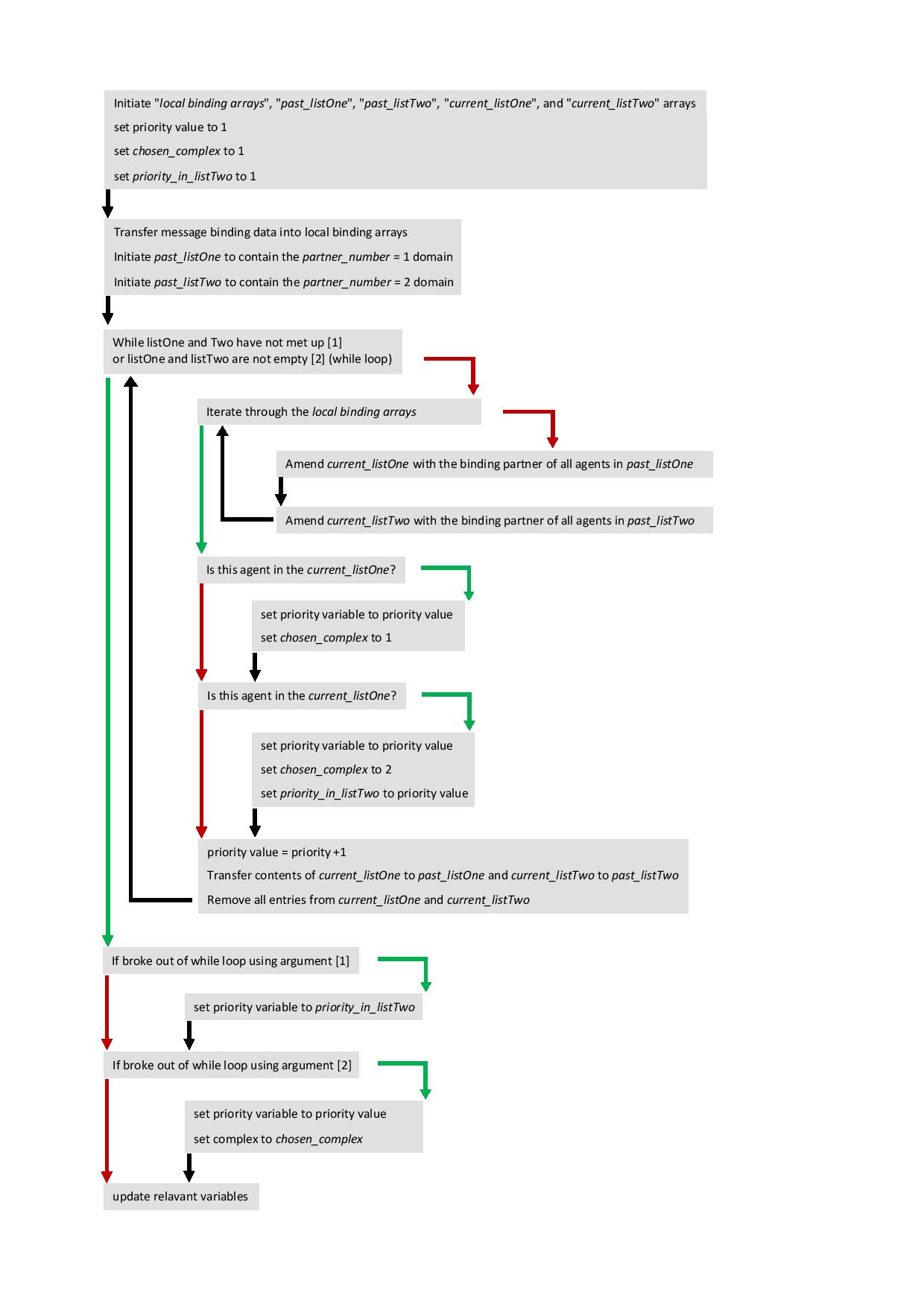 |
| --- |
| **Figure S15. Simplified flowchart of priority recalculation following the unbinding phase**. Arrows show the direction of code with red paths being followed when a condition of a statement is not met, green when the condition is met, and black being followed regardless. Each agent essentially “walks” along both possible complexes (one starting from each of the domains involved in the unbinding interaction) until either [1] the same platform is identified in both complex walks, or [2] both walks finish without sharing a platform. These complexes are termed “1” and “2” in the figure, although their values in the code are derived from the layer 31 message list. The case of [1] signifies that both lists have “met up” via another connection and thus all proteins associated with the unbinding event are still part of the same complex. Here, each platform takes its priority value from the second domain involved in the unbind interaction. The case of [2] signifies that both lists have exhausted all possible elements and yet never met up. Here, both the proteins that unbound now belong to different complexes. The agent takes the priority calculated in the while loop and the complex ID associated with the list that identified the agent. |

Third, both lists continue until completion without “meeting up” and the platform identifies itself within the dominant binder list. Here, the agent accepts the binding priority value from the dominant binder walk and complex ID of *new_complexOne_id* (layer 31 message).

Regardless of what outcome was reached, a message is sent containing the complex_id value in preparation for a recalculation of the size variable.

### 8.34 Layer 33: Unbinding priority update: disseminating priority values

| Agent’s subject | >currentState  nextState> | Function guard | >Message input  Message output> |
| --- | --- | --- | --- |
| Function: output_unbinding_platform_variables | | | |
| PlatformAgent | >Resolved  Resolved> | Agent identified its complex in layer 32 | >complex_size_count  platform_variable_update> |

Agents that identified their *complex_id* at the start of layer 32 may be within a complex that is a different size than at the start of the unbinding phase. Therefore, agents update their complex_size variable with how many other PlatformAgents share the same complex_id as themselves. Platforms also output priority and size information as a message.

### 8.35 Layer 34: Unbinding priority update: updating domains

| Agent’s subject | >currentState  nextState> | Function guard | >Message input  Message output> |
| --- | --- | --- | --- |
| Function: update_unbinding_domain_priority_values | | | |
| DomainAgent | >Resolved  Resolved> | Domain with a complex size greater than 1 | >platform_variable_update |

All DomainAgents contained within multi-platform complexes search for the IDs of their parent platform in the layer 33 message list. If found, they update their complex ID, priority, and complex size accordingly.

### 8.36 Layer 35: Updating DomainAgent context 2: uploading data

| Agent’s subject | >currentState  nextState> | Function guard | >Message input  Message output> |
| --- | --- | --- | --- |
| Function: binding_report_requests | | | |
| DomainAgent | >Resolved  Resolved> |  | upload_partners> |

The context of DomainAgents must be refreshed after any possible unbinding events. This uses the same function as described in layer 23.

### 8.37 Layer 36: Updating DomainAgent context 2: reassessing dimerisation status

| Agent’s subject | >currentState  nextState> | Function guard | >Message input  Message output> |
| --- | --- | --- | --- |
| Function: register_dimerisation | | | |
| PlatformAgent | >Resolved  Resolved> | Only proteins which have the possibility to form homodimers | >upload_partners |

PlatformAgents must identify whether they are now homodimerised before sorting through the binding data. This uses the same function as described in layer 24.

### 8.38 Layer 37: Updating DomainAgent context 2: data processing

| Agent’s subject | >currentState  nextState> | Function guard | >Message input  Message output> |
| --- | --- | --- | --- |
| Function: register_binds | | | |
| PlatformAgent | >Resolved  Resolved> |  | >upload_partners  dimerisation_updator_list> |

PlatformAgents run through the binding data messages from layer 35 using the same function as described in layer 25.

### 8.39 Layer 38: Informing the relevant domains and Las17-mediated nucleation

| Agent’s subject | >currentState  nextState> | Function guard | >Message input  Message output> |
| --- | --- | --- | --- |
| Function: register_dimer_binds | | | |
| DomainAgent | >Resolved  Resolved> | Only proteins which have the possibility to form homodimers | >dimerisation_updator_list |
|  |  | Function: **Las17_nucleation** |  |
| PlatformAgent | >Resolved  Resolved> | Platform must be Las17 (*species* = 0) | nucleation_ticket> |

DomainAgents read the output messages of layer 37 to update their environmental context variables. This uses the same function as described in layer 26.

If three actin agents are found to simultaneously bind the same Las17 monomer/dimer, then a new actin FilamentAgent is generated and the appropriate variables for a newly nucleating filament are set. A *nucleation_ticket* message is outputted containing the domain index of the actin-bound tracts and ID of the new filament. The three actin agents which constitute the nucleus must occupy a PP position. However, they can be bound to either the Las17 agent executing the function, the homodimerisation partner of the Las17, or a combination of the two. To prevent both Las17 agents within a dimer simultaneously triggering a nucleation event, only the platform with the highest ID number can execute the function code. If both Las17 agents in a dimer have bound actin at the same tract, then the actin which is bound to the Las17 with the highest ID number is chosen to constitute the seed.

### 8.40 Layer 39: Register nucleation: Las17 domains

| Agent’s subject | >currentState  nextState> | Function guard | >Message input  Message output> |
| --- | --- | --- | --- |
| Function: register_nucleation_tracts | | | |
| DomainAgent | >Resolved  Resolved> | Must be a species 0 platform (Las17) | >nucleation_ticket  g_to_f_ticket> |

Las17 DomainAgents search the layer 38 message list. If they successfully identify themselves, the agent(s) compare their domain_index variable(s) against the tract indexes of the message. If these values match, then the tract unbinds the monomeric actin agent and changes its binding_state variable to 3, indicating the domain is bound to an actin filament. A message is also output containing information for the actin platform including the leapfrog variable, *filament_id*.

### 8.41 Layer 40: Register nucleation: actin

| Agent’s subject | >currentState  nextState> | Function guard | >Message input  Message output> |
| --- | --- | --- | --- |
| Function: register_nucleation_dimer | | | |
| PlatformAgent | >Resolved  Resolved> |  | >nucleation_ticket |
| Function: register_nucleation_actin | | | |
| DomainAgent | >Resolved  Resolved> | Agents must be of type 5 (actin) | >g_to_f_ticket  nucleated_platform_ticket> |
| Function: auto_nucleation | | | |
| PlatformAgent | >Resolved  Resolved> | ID value of 1 |  |

It is critical to update the homodimerisation partner of a Las17 agent that has nucleated Las17. Therefore, PlatformAgents search the layer 38 message list for dimerised partners. If identified, the filament_bound variable is updated accordingly.

Actin domain agents search the layer 39 message list. If they can identify themselves, the agent(s) will update their variables accordingly to reflect their incorporation into a newly nucleated actin seed. This includes using the leapfrog *filament_id* variable to link themselves to the correct filament. A message is then sent to update their parent platforms in the subsequent layer

The PlatformAgent possessing an ID value of “1” generates a random number variable and compares this against the probability of salt-mediated actin nucleation per timestep. If the random number is lower than this probability value, then a new FilamentAgent will be generated. This reflects the process of salt-mediated nucleation in solution whereby actin monomers can directly interact to form a nucleus. *auto_nucleation* functions to reproduce this process whilst avoiding the extreme rates associated with actin dimerisation.

### 8.42 Layer 41: Register nucleation: Las17 platforms

| Agent’s subject | >currentState  nextState> | Function guard | >Message input  Message output> |
| --- | --- | --- | --- |
| Function: update_nucleation_platforms | | | |
| PlatformAgent | >Resolved  Resolved> | Must be a species 1 platform (actin) | >nucleated_platform_ticket |
| Function: update_nucleating_domain_priority_values | | | |
| DomainAgent | >Resolved  Resolved> |  | >nucleation_ticket  nucleating_platform_update_priority> |

The parent platforms of actin DomainAgents that were incorporated into an actin filament during layer 40 identify themselves using the *nucleated_platform_ticket* message list and update their variable accordingly. This includes setting the *factin* variable to “1”.

Nucleation changes the platform makeup of complexes involved. Therefore, priorities and complex sizes need to be redetermined. Domain agents executing the function *update_nucleating_domain_priority_values* search for complex IDs. If located, they output their binding information. Domains with platform ID numbers matching the Las17 agent that nucleated a filament are identified as “*priority_prime*”.

### 8.43 Layer 42: Nucleation priority update: determining priority

| Agent’s subject | >currentState  nextState> | Function guard | >Message input  Message output> |
| --- | --- | --- | --- |
| Function: update_nucleating_platform_priority_values | | | |
| PlatformAgent | >Resolved  Resolved> |  | >nucleating_platform_update_priority  nucleating_complex_id_counter> |

Platform agents redetermine their priority values using code derived from the binding phase. This time, the list walk begins from the parent platform of the “*priority_prime*” agent identified in layer 41 and a message is output containing the *complex_id*.

### 8.44 Layer 43: Nucleation priority update: disseminating priority

| Agent’s subject | >currentState  nextState> | Function guard | >Message input  Message output> |
| --- | --- | --- | --- |
| Function: output_nucleating_platform_priorities | | | |
| PlatformAgent | >Resolved  Resolved> | Agents must have updated their priority values in layer 40 | >nucleating_complex_id_counter  nucleating_final_update> |

Platforms that updated their priorities in layer 42 use the message input to count the number of PlatformAgents with matching *complex_id* values to recalculate the *complex_size* variable. They then output the priority and size for their child DomainAgents.

### 5.6.45 Layer 44: Nucleation priority update: updating domains

| Agent’s subject | >currentState  nextState> | Function guard | >Message input  Message output> |
| --- | --- | --- | --- |
| Function: updating_nucleating_domain | | | |
| DomainAgent | >Resolved  Resolved> | Agents must belong to a complex which nucleated an actin filament this timestep | >nucleating_final_update |

Agents search the output messages from layer 43 and update their priority and complex size variables to those given by their parent PlatformAgents.

Allwood EG, Tyler JJ, Urbanek AN, Smaczynska-de Rooij II, Ayscough KR (2016) Elucidating key motifs required for Arp2/3-dependent and independent actin nucleation by Las17/WASP. Plos One 11:e0163177 doi: 10.1371/journal.pone.0163177

Bateman A, Martin M-J, Orchard S, Magrane M, Ahmad S, Alpi E, Bowler-Barnett EH, Britto R, Cukura A, Denny P, Dogan T, Ebenezer T, Fan J, Garmiri P, Gonzales LJdC, Hatton-Ellis E, Hussein A, Ignatchenko A, Insana G, Ishtiaq R, Joshi V, Jyothi D, Kandasaamy S, Lock A, Luciani A, Lugaric M, Luo J, Lussi Y, MacDougall A, Madeira F, Mahmoudy M, Mishra A, Moulang K, Nightingale A, Pundir S, Qi G, Raj S, Raposo P, Rice DL, Saidi R, Santos R, Speretta E, Stephenson J, Totoo P, Turner E, Tyagi N, Vasudev P, Warner K, Watkins X, Zellner H, Bridge AJ, Aimo L, Argoud-Puy G-l, Auchincloss AH, Axelsen KB, Bansal P, Baratin D, Neto TMB, Blatter M-C, Bolleman JT, Boutet E, Breuza L, Gil BC, Casals-Casas C, Echioukh KC, Coudert E, Cuche B, de Castro E, Estreicher A, Famiglietti ML, Feuermann M, Gasteiger E, Gaudet P, Gehant S, Gerritsen V, Gos A, Gruaz N, Hulo C, Hyka-Nouspikel N, Jungo F, Kerhornou A, Le Mercier P, Lieberherr D, Masson P, Morgat A, Muthukrishnan V, Paesano S, Pedruzzi I, Pilbout S, Pourcel L, Poux S, Pozzato M, Pruess M, Redaschi N, Rivoire C, Sigrist CJA, Sonesson K, Arighi CN, Armin-ski L, Chen C, Chen Y, Huang H, Laiho K, McGarvey P, Natale DA, Ross K, Vinayaka CR, Wang Q, Wang Y, Zhang J, Bye-A-Jee H, Zaru R, Sundaram S, Wu CH, UniProt C (2023) UniProt: the Universal Protein Knowledgebase in 2023. Nucleic Acids Research 51:D523-D31 doi: 10.1093/nar/gkac1052

Chen Q, Pollard TD (2013) Actin filament severing by cofilin dismantles actin patches and produces mother filaments for new patches. Current Biology 23:1154-62 doi: 10.1016/j.cub.2013.05.005

Hancock LP, Palmer J, Allwood EG, Smaczynska-de Rooij II, Rowe ML, Williamson MP, Ayscough KR (2025) Regulation of actin nucleation by competitive binding of actin and SH3 domains at proline-rich regions in yeast Las17/WASp. Communications Biology in pressdoi:

Ho B, Baryshnikova A, Brown GW (2018) Unification of protein abundance datasets yields a quantitative *Saccharomyces cerevisiae* proteome. Cell Systems 6:192-205 doi: 10.1016/j.cels.2017.12.004

Rasson AS, Bois JS, Pham DSL, Yoo H, Quinlan ME (2015) Filament assembly by Spire: Key residues and concerted actin binding. Journal of Molecular Biology 427:824-39 doi: 10.1016/j.jmb.2014.09.002

Sept D, McCammon JA (2001) Thermodynamics and kinetics of actin filament nucleation. Biophysical Journal 81:667-74 doi: 10.1016/s0006-3495(01)75731-1

Sitar T, Gallinger J, Ducka AM, Ikonen TP, Wohlhoefler M, Schmoller KM, Bausch AR, Joel P, Trybus KM, Noegel AA, Schleicher M, Huber R, Holak TA (2011) Molecular architecture of the Spire-actin nucleus and its implication for actin filament assembly. Proceedings of the National Academy of Sciences of the United States of America 108:19575-80 doi: 10.1073/pnas.1115465108

Tonikian R, Xin X, Toret CP, Gfeller D, Landgraf C, Panni S, Paoluzi S, Castagnoli L, Currell B, Seshagiri S, Yu H, Winsor B, Vidal M, Gerstein MB, Bader GD, Volkmer R, Cesareni G, Drubin DG, Kim PM, Sidhu SS, Boone C (2009) Bayesian modeling of the yeast SH3 domain interactome predicts spatiotemporal dynamics of endocytosis proteins. Plos Biology 7:e1000218 doi: 10.1371/journal.pbio.1000218

Williamson MP (2023) Protein binding: A fuzzy concept. Life-Basel 13:855 doi: 10.3390/life13040855

Zuchero JB, Coutts AS, Quinlan ME, La Thangue NB, Mullins RD (2009) p53-cofactor JMY is a multifunctional actin nucleation factor. Nature Cell Biology 11:451-59 doi: 10.1038/ncb1852
